# Three-component synthesis of C_2_F_5_-substituted pyrazoles from C_2_F_5_CH_2_NH_2_·HCl, NaNO_2_ and electron-deficient alkynes

**DOI:** 10.3762/bjoc.11.3

**Published:** 2015-01-06

**Authors:** Pavel K Mykhailiuk

**Affiliations:** 1 Enamine Ltd., Vul. Oleksandra Matrosova 23, 01103 Kyiv, Ukraine; and Department of Chemistry, Taras Shevchenko National University of Kyiv, Volodymyrska Street, 64, Kyiv 01601, Ukraine

**Keywords:** cycloaddition, fluorine, pentafluoroethyl group, pentafluoroethyldiazomethane, pyrazole

## Abstract

A one-pot reaction between C_2_F_5_CH_2_NH_2_·HCl, NaNO_2_ and electron-deficient alkynes gives C_2_F_5_-substituted pyrazoles in excellent yields. The transformation smoothly proceeds in dichloromethane/water, tolerates the presence of air, and requires no purification of products by column chromatography. Mechanistically, C_2_F_5_CH_2_NH_2_·HCl and NaNO_2_ react first in water to generate C_2_F_5_CHN_2_, that participates in a [3 + 2] cycloaddition with electron-deficient alkynes in dichloromethane.

## Introduction

**Figure 1:**
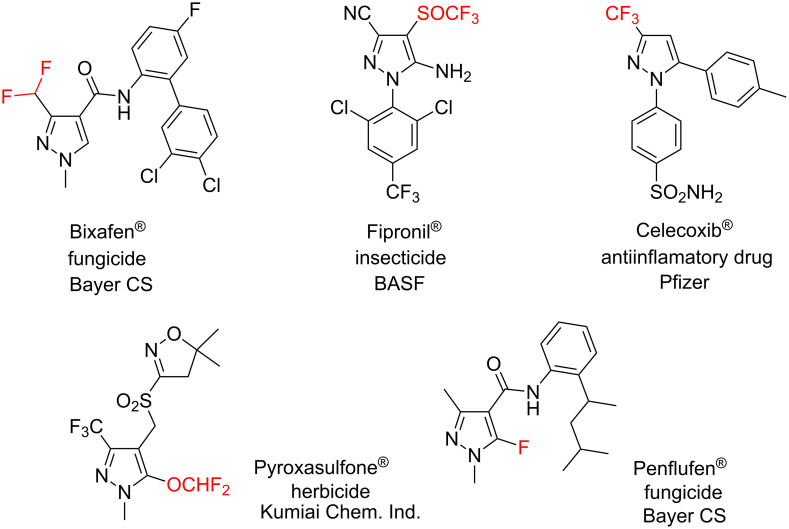
Bioactive compounds and agrochemicals with fluorinated pyrazoles.

Incorporation of fluorinated fragments into organic compounds might affect their physicochemical and biological properties [1–8]. It is not surprising, therefore, that up to 20% of all modern drugs contain at least one fluorine atom [9–11]. Fluorinated pyrazoles, for example, do play a role in medicinal chemistry: among them are inhibitors of the measles virus RNA polymerase complex [12], CRAC channel, cyclogenases [13–15], 5-lipoxygenase [16], heat shock protein 90 [17]; inducers of G0–G1 phase arrest [18], modulators of the AMPA receptor [19], activators of the Kv7/KCNQ potassium channel [20], regulators of the NFAT transcription factor [21], etc. Approved drugs and agrochemicals – Bixafen, Fipronyl, Celecoxib, Pyroxasulfone and Penflufen – are also pyrazoles with diverse fluorine-containing substituents (Figure 1) [22–23]. Moreover, fluorinated NH-pyrazoles have also found an application as ligands in coordinational chemistry [24–27].

**Figure 2:**
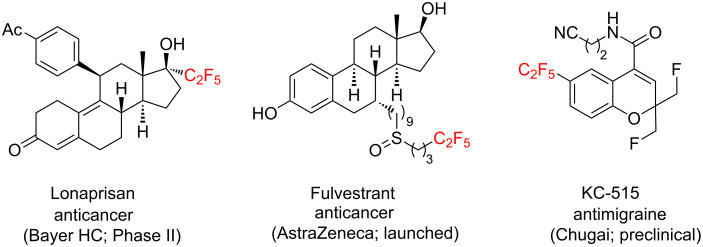
Bioactive compounds with C_2_F_5_ group [34–36].

While the trifluoromethylated derivatives play a major role in chemistry [28], their more lipophilic analogues – C_2_F_5_ [1,29–30] and SF_5_ [1,31] – only gain popularity. The C_2_F_5_-substituted derivatives, for example, often have higher activity compared with the CF_3_-counterparts [32–33]. Therefore, some bioactive compounds including several drugs contain a C_2_F_5_ group (Figure 2) [34–36].

The conceptually attractive C_2_F_5_-pyrazoles [37], however, still remain somewhat in the shadow [38], probably because of the lack of the corresponding chemical approaches. Predominantly, C_2_F_5_-pyrazoles are synthesized by a reaction of 1,3-dicarbonyl compounds (or their synthons) with hydrazines [39–44]. In this context, novel practical methods to C_2_F_5_-pyrazoles are needed.

Last year, Ma and colleagues synthesized CF_3_-pyrazoles by [3 + 2] cycloaddition between in situ generated CF_3_CHN_2_ and alkynes [45]. This method, however, required a) the preliminary preparation of a dry solution of toxic CF_3_CHN_2_; b) the use of inert atmosphere; c) an addition of a catalyst (Ag_2_O); and d) purification of products by column chromatography. Also, the reaction worked only for mono-substituted alkynes. In parallel, an alternative practical approach to CF_3_-pyrazoles by a three-component reaction between electron-deficient alkynes, sodium nitrite and trifluoroethylamine hydrochloride was developed [46]. This method worked for both the mono- and disubstituted alkynes. Because of similar electronic properties of CF_3_ and C_2_F_5_ groups [1], it was supposed that a reaction between in situ generated C_2_F_5_CHN_2_ [47], and electron-deficient alkynes would lead to C_2_F_5_-pyrazoles. In this work, this hypothesis was proven; the scope and selectivity of this transformation was studied and the high practical potential of the developed method is shown.

## Results and Discussion

### Validation and optimization

To challenge the putative transformation, the simple mono-substituted alkyne **1** with one electron-withdrawing CO_2_Me-group (EWG) was selected. A mixture of alkyne **1**, C_2_F_5_CH_2_NH_2_·HCl (1.0 equiv) [48] and NaNO_2_ (2.0 equiv) in water/dichloromethane was stirred at room temperature. After 10 min the organic layer became yellow indicating the formation of C_2_F_5_CHN_2_. After 24 h the reaction conversion was 55%, but no side products were observed. Optimization of the reaction conditions – C_2_F_5_CH_2_NH_2_·HCl (3.0 equiv), NaNO_2_ (5.0 equiv), 72 h – allowed achieving the full reaction conversion (Table 1, entry 6). The standard work-up afforded pyrazole **1a** as a white crystalline solid in 99% yield without any purification (neither recrystallization, nor column chromatography). The reaction required no inert gas atmosphere, and was performed in air.

**Table 1:** Optimization of the reaction conditions.

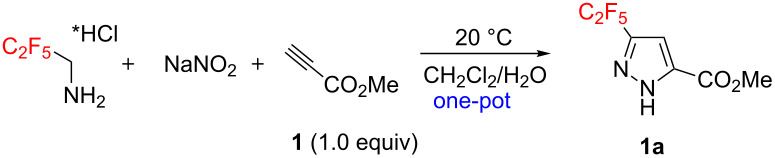				

Entry	C_2_F_5_CH_2_NH_2_·HCl (equiv)	NaNO_2_ (equiv)	Time (h)	Conversion (%)

1	1.0	2.0	24	55
2	1.0	2.0	72	76
3	2.0	3.0	24	75
4	2.0	3.0	72	95
5	3.0	5.0	24	96
6	3.0	5.0	72	100

### Reaction scope

Having these encouraging results on pyrazole **1a** at hand, the reaction scope was studied. First, various mono-substituted alkynes **2**–**14** were tested under the already optimized reaction conditions (Table 2).

**Table 2:** Synthesis of C_2_F_5_-substituted pyrazoles from mono-substituted alkynes.

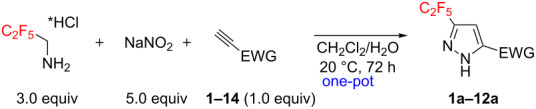						

Entry	Alkyne		Product		Yield (%)^a^	

1	**1**	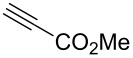	**1a**	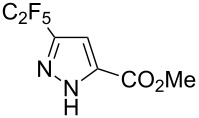	99	
2	**2**	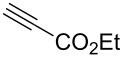	**2a**	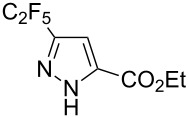	98	
3	**3**	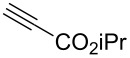	**3a**	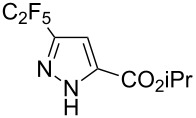	97	
4	**4**	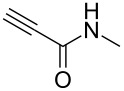	**4a**	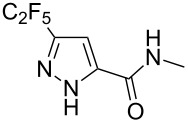	59 (76)^b^ 78^c^	
5	**5**	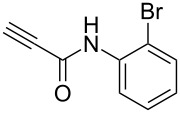	**5a**	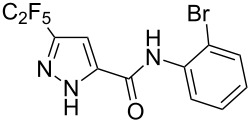	22 (55)^b^ 88^c^	
6	**6**	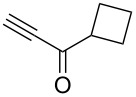	**6a**	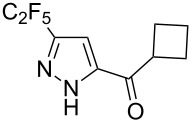	99	
7	**7**	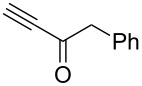	**7a**	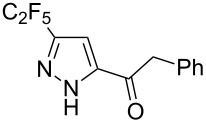	98	
8	**8**	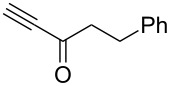	**8a**	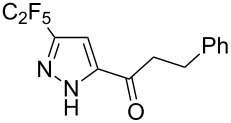	97	
9	**9**	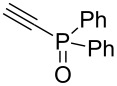	**9a**	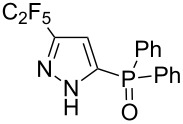	95	
10	**10**	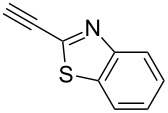	**10a**	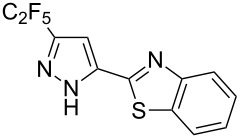	23 (33)^b^ 45^c^ X-ray	
11	**11**	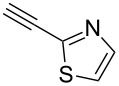	**11a**	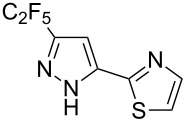	57^d^	
12	**12**	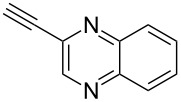	**12a**	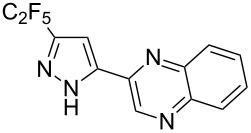	38^d^	

13	**13**	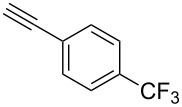	no reaction		–^b,c^	
14	**14**	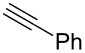	no reaction		–^b,c^	

^a^Isolated yields. ^b^rt, 168 h, dichloromethane/water. The reaction conversion is in brackets: ( ). ^c^45 °C, 72 h, toluene/water. ^d^40 °C, 72 h, dichloromethane/water.

Substrates **2**, **3**, **6–9** with strong EWGs smoothly reacted with C_2_F_5_CHN_2_ at room temperature to afford products **2a**, **3a**, **6a**–**9a** in excellent yields of 95–99% without any purification. Substrates **4**, **5** and **10–12** with weak EWG reacted slowly, and even after one week the reaction was not complete (33–76% conversion). The pure products **4a**, **5a**, and **10a–12a** (Table 2) were obtained in bad yields after crystallization of the reaction mixtures. For these compounds, however, the yields were improved to 38–88% by performing the reaction at increased temperature: 40–45 °C.

This method, however, did not work for aromatic alkynes with either weak EWG (**13**) or electron-donating group (EDG, **14**) – all attempts to react substrates **13** and **14** failed. These results suggest that the reaction between C_2_F_5_CHN_2_ and alkynes belongs to type I of [3 + 2] cycloadditions [49]: It is accelerated by the alkyne`s EWGs and decelerated by EDGs.

Next diverse disubstituted alkynes **15**–**22** (Table 3) with at least one strong EWG (–CO_2_Alk or –COAlk) were studied. Substrates **15**–**18** with the second EWG smoothly reacted with C_2_F_5_CHN_2_ to afford pyrazoles in almost quantitative yield. Substrate **19** with the second EDG (SiMe_3_), however, reacted slowly. The reaction conversion was 52% after 7 days leading to the sole regioisomer **19b** without side products. The crystalline product **19b** was purified from the liquid starting material by washing with cyclohexane. The yield of **19b** was improved to 73% by performing the reaction at 40 °C (Table 3, entry 5). SiMe_3_-substituted substrates **20**, **21** also reacted slowly, but again performing the reaction at 40 °C allowed to obtain the target products **20b**, **21a**/**b** in good yields. Substrate **22** with the second EDG – aryl – did not react, however.

**Table 3:** Synthesis of C_2_F_5_-substituted pyrazoles from disubstituted alkynes.

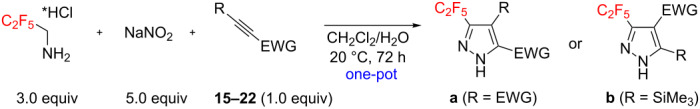						

Entry	Alkyne		Product			Yield (%)^a^

1	**15**	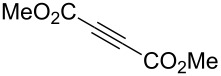	**15a**	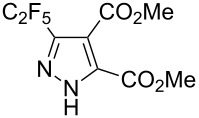		99
2	**16**	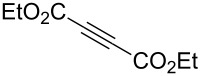	**16a**	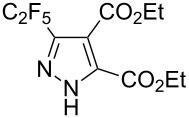		98
3	**17**	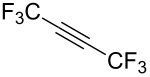	**17a**	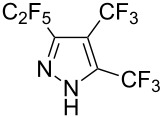		65
4	**18**	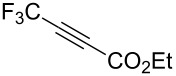	**18a**/**b** (2.6/1.0)	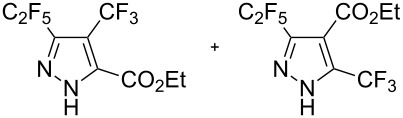		99^b^
5	**19**	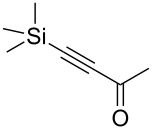	**19b**	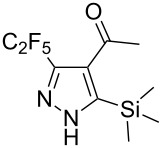		43 (52)^c^ 73^d^ X-ray
6	**20**	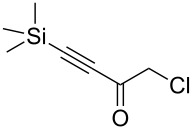	**20b/a** (4.0/1.0)	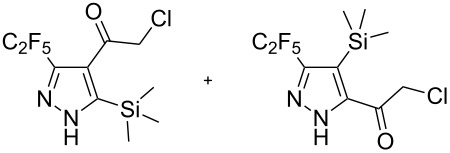		62^e^ X-ray
7	**21**	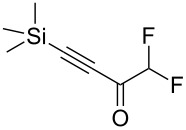	**21b/a** (2.6/1.0)	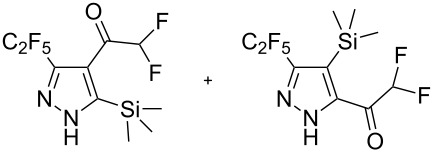		89^b^
8	**22**	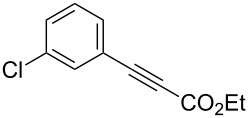	no reaction^c,d^			

^a^Isolated yields. ^b^Yield of the inseparable mixture of pyrazoles. ^c^rt, 168 h, dichloromethane/water. Conversion of the reaction is in brackets: ( ). ^d^40 °C, 72 h, dichloromethane/water. ^e^A mixture of **20b**/**20a** (4/1) is formed, from which pure isomer **20b** is isolated by crystallization in 62% yield.

**Figure 3:**
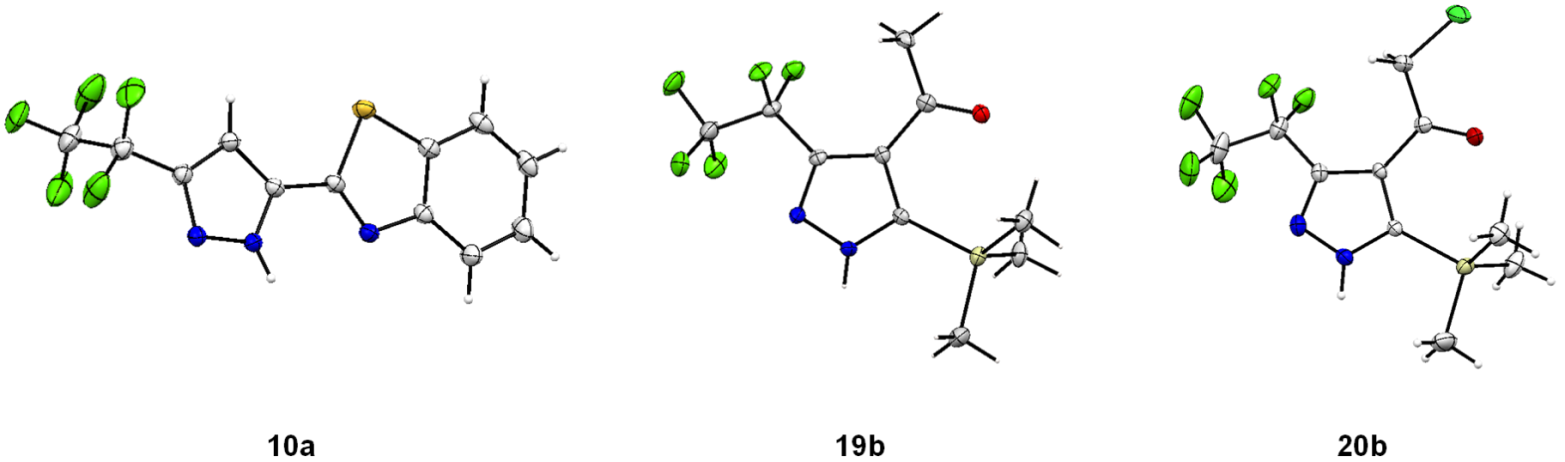
X-ray crystal structure of pyrazoles **10a**, **19b** and **20b** [50].

Structures of compounds **10a**, **19b**, **20b** were confirmed by X-ray crystal structure analysis (Figure 3) [50].

### Reaction regioselectivity

Monosubstituted alkynes **1**–**12** regioselectively reacted with C_2_F_5_CHN_2_ to give only 3,5-disubstituted pyrazoles. Formation of 3,4-disubstituted isomers was observed.

**Figure 4:**
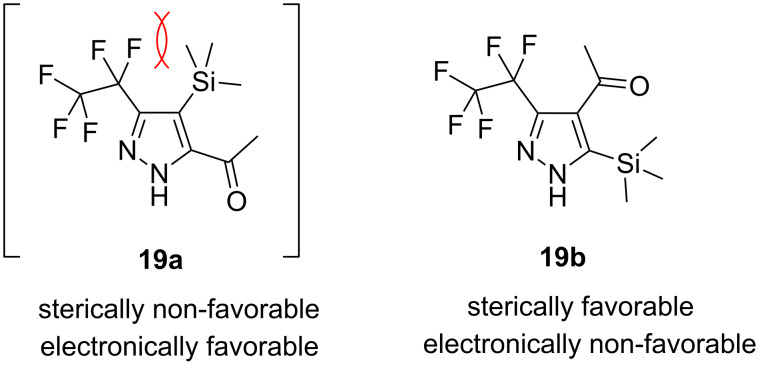
Structure of the expected product **19a**, and the observed isomeric one – **19b**.

Disubstituted alkynes behaved differently, because of controversial electronic and steric effects. According to the orbital symmetry rules, the [3 + 2] cycloaddition must lead to pyrazoles with the C_2_F_5_ group and EWG at 3,5-positions [49]. Product **18a/b** was obtained as a mixture of isomers because the C_2_F_5_ and CO_2_Et had similar electron-withdrawing nature. On the contrary, reaction of alkyne **19** having both the electron-withdrawing (–COMe) and electron-donating (–SiMe_3_) substituents afforded the isomer **19b** with EWG at the 4^th^ position, violating the orbital symmetry rules (Figure 3 and Figure 4). Presumably, the steric repulsion between bulky C_2_F_5_ and SiMe_3_ groups forced the reaction to follow the reversed regioselectivity – the steric effect has overcompensated the electronic one (Figure 4) [51–52].

Replacing one hydrogen atom in alkyne **19** by a bulkier chlorine atom – alkyne **20** – led to mixture of products **20b**/**a** = 4.0/1.0 (the pure product **20b** was obtained by crystallization). Replacing two hydrogen atoms in **20** by bulkier fluorine atoms – alkyne **21** – led to mixture **21b**/**a** = 2.6/1.0. In compounds **13**–**15** the steric effect overcompensated the electronic one, but the impact of steric effect decreased with increasing the size of the second substituent: CH_3_CO (**19**), ClCH_2_CO (**20**), HF_2_CCO (**21**) increasing the percentage of α-isomers (0% in **19b**, 20% in **20a**/**b**, 28% in **21a**/**b**).

### Reactivity of C_2_F_5_CHN_2_ vs CF_3_CHN_2_

**Scheme 1:**
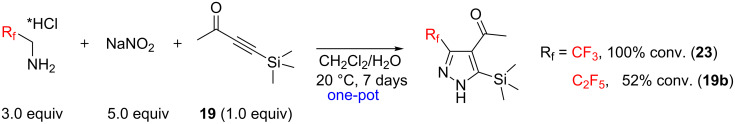
Comparison of CF_3_CHN_2_ *vs* C_2_F_5_CHN_2_ in the reaction with alkyne **19**.

To compare the reactivities of C_2_F_5_CHN_2_ and CF_3_CHN_2_ in [3 + 2] cycloadditions with alkynes, one must take into account two factors: steric and electronic [49]. On one hand, the C_2_F_5_ substituent is bulkier than the CF_3_ one, decreasing thereby the reactivity of C_2_F_5_CHN_2_ compared with CF_3_CHN_2_. On the other hand, C_2_F_5_ and CF_3_ groups have similar electron-withdrawing abilities [1], and hence the both diazoalkanes might have similar reactivities. In reality, under the same conditions, after seven days at room temperature alkyne **19** reacted with CF_3_CHN_2_ completely, while the corresponding reaction with C_2_F_5_CHN_2_ reached only 52% conversion. Obviously, the steric effect overcompensated the electronic one; and C_2_F_5_CHN_2_ was less active than CF_3_CHN_2_ (Scheme 1).

Another factor, however, must be kept in mind while comparing the reactivities of C_2_F_5_CHN_2_ and CF_3_CHN_2_: the reactions of C_2_F_5_CHN_2_ can be safely heated up to 40–45 °C without evaporation of the reagent while those with CF_3_CHN_2_ cannot (bp = 13 °C) [53]. Therefore, the yield of product **19b** was improved to 73% by performing the reaction at 40 °C.

### Selected chemical transformations

**Scheme 2:**
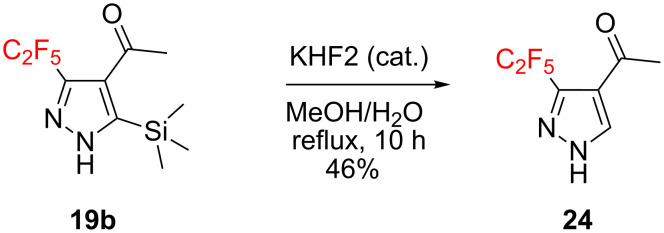
Synthesis of 3,4-disubstituted pyrazole **24**.

Having developed a robust practical method to C_2_F_5_-pyrazoles, the true practical potential of the obtained compounds was demonstrated. First, the standard cleavage of TMS group in **19b** led to ketone **24** (Scheme 2). This strategy gives an opportunity for preparing the 3,4-disubstituted pyrazoles from TMS-derivatives, while the direct reaction of monosubstituted alkynes **1**–**12** with C_2_F_5_CHN_2_ gives 3,5-disubstituted ones.

**Scheme 3:**
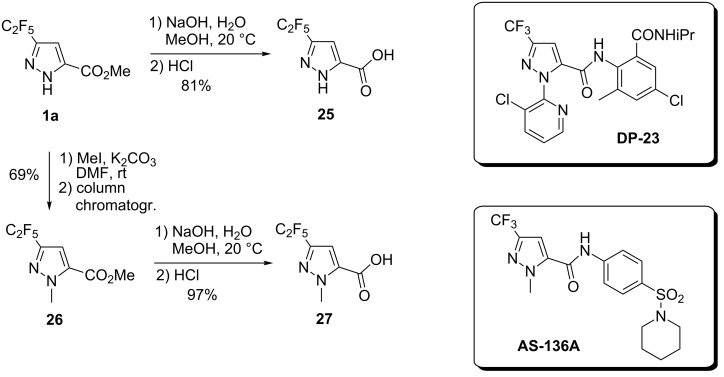
Synthesis of C_2_F_5_-substituted acids **25** and **27**. In brackets are the known bioactive compounds **DP-23**, **AS-136A** (CF_3_-analogues of **25** and **27**).

Also, alkaline hydrolysis of the ester group in **1a** gave acid **25** – potential building blocks for medicinal chemistry and drug discovery: many bioactive compounds, including the insecticidal agent **DP**-**23**, contain the residue of the CF_3_-analogue of **25** (Scheme 3) [54].

Finally, alkylation of pyrazole **1a** with MeI afforded the product **26** in 69% yield after column chromatography. Basic hydrolysis of the ester group in **26** gave acid **27** – another potential building block for drug discovery (the corresponding CF_3_-acid constitutes to the known antiviral agent **AS-136A** [55]) (Scheme 3).

## Conclusion

In summary, a novel approach to C_2_F_5_-substituted pyrazoles has been elaborated by a three-component reaction between C_2_F_5_CH_2_NH_2_·HCl, NaNO_2_ and electron-deficient alkynes. This method is highly practical: it does not require a) the pre-isolation of toxic diazo intermediates; b) inert gas atmosphere (the reaction is performed in air); c) any catalysts; d) purification of the products by column chromatography. Also, the reaction works for both the mono- and disubstituted alkynes; and allows preparing of 3,5- and 3,4-disubstituted pyrazoles (via SiMe_3_-alkynes).

Therefore, given the importance of fluorinated pyrazoles, it is desirable that scientists will soon use this extremely useful practical reaction in synthetic organic chemistry, drug discovery and agrochemistry (where the need for robust reactions is high).

## Experimental

**General procedure**: To a stirred suspension of C_2_F_5_CH_2_NH_2_·HCl (90 mg, 0.48 mmol, 3.0 equiv) in CH_2_Cl_2_ (4.0 mL)/water (0.2 mL), sodium nitrite (54 mg, 0.78 mmol, 5.0 equiv) and alkyne (0.16 mmol, 1.0 equiv) were added. The reaction mixture was vigorously stirred at 20 °C for 72 h. Water (1.0 mL) and CH_2_Cl_2_ (3 mL) were added. The organic layer was separated. The aqueous layer was washed with CH_2_Cl_2_ (2 × 3 mL). The combined organic layers were dried over Na_2_SO_4_ and evaporated under vacuum to provide the pure product.

## Supporting Information

File 1 Experimental procedures and copies of NMR spectra for all new compounds.
