# Supplementary material for: Three-component synthesis of C2F5-substituted pyrazoles from C2F5CH2NH2·HCl, NaNO2 and electron-deficient alkynes
Source: Beilstein J Org Chem. 2015 Jan 6;11:16–24. doi: 10.3762/bjoc.11.3 (PMC4311722; doi:10.3762/bjoc.11.3)
Supplement: File 1 — Experimental procedures and copies of NMR spectra for all new compounds. [file Beilstein_J_Org_Chem-11-16-s001.pdf]

Supporting Information
for

Three-component synthesis of C_2F_5-substituted pyrazoles from $C_2F_5CH_2NH_2 \cdot HCl$, $NaNO_2$ and electron-deficient alkynes

Pavel K. Mykhailiuk

Address: Enamine Ltd., Vul. Oleksandra Matrosova 23, 01103 Kyiv, Ukraine; and Department of Chemistry, Taras Shevchenko National University of Kyiv, Volodymyrska Street, 64, Kyiv 01601, Ukraine

Email: Pavel K. Mykhailiuk - Pavel.Mykhailiuk@gmail.com

Experimental procedures and copies of NMR spectra for all new compounds

Experimental part

Dichloromethane was purified by distillation. All reagents were available from Enamine Ltd. Melting points are uncorrected. ^{1}H- and ^{13}C-NMR spectra were recorded on a Bruker Avance 500 spectrometer (at 499.9 MHz and 124.9 MHz, respectively). ^{19}F-NMR spectra were recorded on a Varian Unity Plus 400 spectrometer (at 376.7 Hz). Chemical shifts are reported in ppm downfield from Me_4Si (^{1}H, ^{13}C) or upfield from $CFCl_3$ (^{19}F) using conventional deuterium lock referencing as internal standards. MS analysis was performed on an LCMS instrument with chemical ionization.

General procedure: To a stirred suspension of $C_2F_5CH_2NH_2 \cdot HCl$ (90 mg, 0.48 mmol, 3.0 eq.) in CH_2Cl_2 (4.0 mL) / water (0.2 mL), sodium nitrite (54 mg, 0.78 mmol, 5.0 eq.) and alkyne (0.16 mmol, 1.0 eq.) was added. The reaction mixture was vigorously stirred 72 h at 20 ºC. Water (1.0 mL) and CH_2Cl_2 (3 mL) were added. The organic layer was separated. The aqueous layer was washed with CH_2Cl_2 (2 × 3 mL). The combined organic layers were dried over Na_2SO_4 and evaporated under vacuum to provide the pure product.

Methyl 3-(pentafluoroethyl)-1*H*-pyrazole-5-carboxylate (1a)

Compound **1a** was obtained as a white solid (39 mg, 99%) following the general procedure. M.p. = 79-80 ºC.

^{1}H NMR (500 MHz; $CDCl_3$; Me_4Si), δ: 11.81 (broad s, 1H, N*H*), 7.11 (s, 1H, C*H*), 3.96 (s, 3H, C*H*$_3$).

^{13}C NMR (125 MHz; $CDCl_3$; Me_4Si), δ: 159.0 (s, CO), 142.8 (broad s, *C*), 135.5 (broad s, *C*), 118.6 (qt, $^1J_{C\text{-}F}$ = 285.0 Hz, $^2J_{C\text{-}F}$ = 36.3 Hz, $CF_2\mathit{C}F_3$), 110.2 (tq, $^1J_{C\text{-}F}$ = 250.0 Hz, $^2J_{C\text{-}F}$ = 38.9 Hz, $\mathit{C}F_2CF_3$), 108.6 (s, *C*H), 52.7 (s, $\mathit{C}H_3$).

^{19}F NMR (375 MHz; $CDCl_3$; $CFCl_3$), δ: -85.1 (s, 3F, $CF_2C\mathit{F}_3$), -113.8 (s, 2F, $C\mathit{F}_2CF_3$).

MS (CI): *m/z* (%) = 245 $[M+1]^+$.

Anal. calcd for $C_7H_5F_5N_2O_2$: C, 34.44; H, 2.06; N, 11.48. Found: C, 34.14; H, 2.34; N, 11.68.

Ethyl 3-(pentafluoroethyl)-1*H*-pyrazole-5-carboxylate (2a)

Compound **2a** was obtained as a white solid (41 mg, 98%) following the general procedure. M.p. = 90 ºC.

^{1}H NMR (500 MHz; $CDCl_3$; Me_4Si), δ: 11.89 (broad s, 1H, N*H*), 7.13 (s, 1H, C*H*), 4.45 (q, *J* = 7.0 Hz, 2H, $C\mathit{H}_2CH_3$), 1.42 (t, *J* = 7.0 Hz, 3H, $CH_2C\mathit{H}_3$).

^{13}C NMR (125 MHz; $CDCl_3$; Me_4Si), δ: 158.3 (s, CO), 142.2 (broad s, *C*), 135.4 (broad s, *C*), 118.3 (qt, $^1J_{C\text{-}F}$ = 285.3 Hz, $^2J_{C\text{-}F}$ = 36.3 Hz, $CF_2\mathit{C}F_3$), 109.8 (tq, $^1J_{C\text{-}F}$ = 250.0 Hz, $^2J_{C\text{-}F}$ = 38.0 Hz, $\mathit{C}F_2CF_3$), 108.2 (s, *C*H), 61.8 (s. $O\mathit{C}H_2$), 13.7 (s, $\mathit{C}H_3$).

^{19}F NMR (375 MHz; $CDCl_3$; $CFCl_3$), δ: -85.1 (t, ^3J(F,F) = 3.8 Hz, 3F, $CF_2C\mathit{F}_3$), -113.8 (q, ^3J(F,F) = 3.8 Hz, 2F, $C\mathit{F}_2CF_3$).

MS (CI): *m/z* (%) = 259 $[M+1]^+$.

Anal. calcd for $C_8H_7F_5N_2O_2$: C, 37.22; H, 2.73; N, 10.85. Found: C, 37.51; H, 3.02; N, 10.74.

Isopropyl 3-(pentafluoroethyl)-1*H*-pyrazole-5-carboxylate (3a)

Compound **3a** was obtained as a white solid (42 mg, 97%) following the general procedure. M.p. = 85-86 ºC.

^{1}H NMR (500 MHz; $CDCl_3$; Me_4Si), δ: 7.12 (s, 1H, C*H*), 5.31 (m, J = 6.5 Hz, 1H, C*H*CH_3), 1.38 (6, J = 6.5 Hz, 6H, CHCH_3).

^{13}C NMR (125 MHz; $CDCl_3$; Me_4Si), δ: 158.0 (s, CO), 141.8 (t, $^2J_{C-F}$ = 30.0 Hz, *C*CF_2), 135.7 (broad s, *C*), 118.2 (qt, $^1J_{C-F}$ = 285.3 Hz, $^2J_{C-F}$ = 36.3 Hz, CF_2*C*F_3), 109.9 (tq, $^1J_{C-F}$ = 250.0 Hz, $^2J_{C-F}$ = 38.0 Hz, *C*F_2CF_3), 108.1 (s, *C*H), 70.1 (s. O*C*H), 21.3 (s, *C*H_3).

^{19}F NMR (375 MHz; $CDCl_3$; $CFCl_3$), δ: -85.1 (s, 3F, CF_2C*F_3*), -113.8 (s, 2F, C*F_2*CF_3).

MS (CI): *m/z* (%) = 273 $[M+1]^+$.

Anal. calcd for $C_9H_9F_5N_2O_2$: C, 39.72; H, 3.33; N, 10.29. Found: C, 39.59; H, 3.61; N, 10.14.

_N_-Methyl-3-(pentafluoroethyl)-1_H_-pyrazole-5-carboxamide (4a)

The reaction was performed in toluene (4.0 mL) /water (0.2 mL) at 45 ºC following the general procedure. The crude product was washed with cyclohexane (0.3 mL) to give the pure compound **4a** as a white solid (30 mg, 78%). M.p. = 172-173 ºC.

^{1}H NMR (500 MHz; CD_3OD; Me_4Si), δ: 7.09 (s, 1H, C*H*), 2.90 (s, 3H, C*H_3*).

^{13}C NMR (125 MHz; CD_3OD; Me_4Si), δ: 158.9 (s, CO), 144.3 (s, *C*), 140.8 (broad s, *C*), signals of CF_2CF_3 are not seen, 103.4 (s, *C*H), 24.5 (s, *C*H_3).

^{19}F NMR (375 MHz; CD_3OD; $CFCl_3$), δ: -85.4 (broad s, 3F, CF_2C*F_3*), -113.3 (broad s, 2F, C*F_2*CF_3).

MS (CI): *m/z* (%) = 244 $[M+1]^+$.

Anal. calcd for $C_7H_6F_5N_3O$: C, 34.58; H, 2.49; N, 17.28. Found: C, 34.25; H, 2.77; N, 17.51.

_N_-(2-bromophenyl)-3-(pentafluoroethyl)-1_H_-pyrazole-5-carboxamide (5a)

The reaction was performed in in toluene (4.0 mL) /water (0.2 mL) at 45 ºC following the general procedure. The crude product was washed with chloroform (0.3 mL) to give the pure compound **5a** as a white solid (54 mg, 78%). M.p. = 152-153 ºC.

^{1}H NMR (500 MHz; DMSO-d_6; Me_4Si), δ: 14.81 (s, 1H, N*H*), 10.37 (s, 1H, N*H*), 7.74 (d, ^3J(H,H) = 10.5 Hz, 1H, *Ph*), 7.57 (s, 1H, *CH*), 7.52 (broad s, 1H, *Ph*), 7.46 (t, $^3J_{H-H}$ = 9.0 Hz, 1H, *Ph*), 7.27 (t, ^3J(H,H) = 9.0 Hz, 1H, *Ph*).

^{13}C NMR (125 MHz; DMSO-d_6; Me_4Si), δ: 156.7 (s, CO), 139.9 (t, $^2J_{C-F}$ = 31.1 Hz, *C*CF_2), 138.7 (s), signals of CF_2CF_3 are not seen, 135.2 (s), 133.0 (s), 129.4 (s), 128.8 (s), 128.4 (s), 120.9 (s), 105.5 (s, *C*H).

^{19}F NMR (375 MHz; DMSO-d_6; $CFCl_3$), δ: -83.2 (broad s, 3F, CF_2C*F_3*), -110.6 (broad s, 2F, C*F_2*CF_3).

MS (CI): *m/z* (%) = 383, 385 $[M+1]^+$.

Anal. calcd for $C_{12}H_7BrF_5N_3O$: C, 37.52; H, 1.84; N, 10.94. Found: C, 37.73; H, 2.07; N, 10.58.

Cyclobutyl[3-(pentafluoroethyl)-1_H_-pyrazol-5-yl]methanone (6a)

Compound **6a** was obtained as a yellow solid (43 mg, 99%) following the general procedure. M.p. = 67-68 ºC.

^{1}H NMR (500 MHz; $CDCl_3$; Me_4Si), δ: 11.78 (broad s, 1H, N*H*), 6.56 (s, 1H, C*H*), 3.82 (qv, J = 7.0 Hz, 1H, C*H*), 2.46 (m, 2H), 2.33 (m, 2H), 2.14 (m, 1H), 1.98 (m, 1H).

^{13}C NMR (125 MHz; $CDCl_3$; Me_4Si), δ: 191.5 (s, CO), 142.2 ($^2J_{C-F}$ = 31.3 Hz, *C*CF_2), 140.4 (broad s, *C*), 118.3 (qt, $^1J_{C-F}$ = 285.3 Hz, $^2J_{C-F}$ = 36.3 Hz, CF_2*C*F_3), 109.8 (tq, $^1J_{C-F}$ = 250.0 Hz, $^2J_{C-F}$ = 38.0 Hz, *C*F_2CF_3), 107.2 (s, *C*H), 42.5 (s. *C*H), 24.3 (s, *C*H_2), 17.8 (s, *C*H_2).

^{19}F NMR (375 MHz; $CDCl_3$; $CFCl_3$), δ: -85.0 (broad s, 3F, CF_2CF_3), -113.5 (broad s, 2F, CF_2CF_3).

MS (CI): *m/z* (%) = 269 $[M+1]^+$.

Anal. calcd for $C_{10}H_9F_5N_2O$: C, 44.79; H, 3.38; N, 10.45. Found: C, 45.02; H, 3.61; N, 10.16.

1-[3-(Pentafluoroethyl)-1*H*-pyrazol-5-yl]-2-phenylethanone (7a)

Compound **7a** was obtained as a white solid (49 mg, 98%) following the general procedure. M.p. = 75-76 ºC.

^{1}H NMR (500 MHz; $CDCl_3$; Me_4Si), δ: 11.67 (broad s, 1H, N*H*), 7.36-7.26 (m, 5H, *Ph*), 7.04 (s, 1H, C*H*), 4.16 (s, 2H, C*H*$_2$).

^{13}C NMR (125 MHz; $CDCl_3$; Me_4Si), δ: 187.8 (s, CO), 142.0 (t, *J* = 28.7 Hz, *C*CF_2), 141.3 (s, *C*), 132.0 (s, Ph), 129.1 (s, Ph), 128.6 (s, Ph), 127.3 (s, Ph), 118.3 (qt, $^1J_{C-F}$ = 285.0 Hz, $^2J_{C-F}$ = 36.3 Hz, CF_2*C*F_3), 109.7 (tq, $^1J_{C-F}$ = 250.0 Hz, $^2J_{C-F}$ = 38.9 Hz, *C*F_2CF_3), 108.9 (s, *C*H), 46.1 (s, *C*H_2).

^{19}F NMR (375 MHz; $CDCl_3$; $CFCl_3$), δ: -85.0 (t, ^3J(F,F) = 3.8 Hz, 3F, CF_2CF_3), -113.5 (q, ^3J(F,F) = 3.8 Hz, 2F, CF_2CF_3).

MS (CI): *m/z* (%) = 305 $[M+1]^+$.

Anal. calcd for $C_{13}H_9F_5N_2O$: C, 51.33; H, 2.98; N, 9.21. Found: C, 51.61; H, 3.32; N, 9.07.

1-[3-(Pentafluoroethyl)-1*H*-pyrazol-5-yl]-3-phenyl-1-propanone (8a)

Compound **8a** was obtained as a white solid (49 mg, 97%) following the general procedure. M.p. = 57-58 ºC.

^{1}H NMR (500 MHz; $CDCl_3$; Me_4Si), δ: 7.33-7.21 (m, 5H, *Ph*), 7.04 (s, 1H, C*H*), 4.16 (s, 2H, C*H*$_2$), 3.25 (t, J = 7.5 Hz, 2H, CH_2), 3.09 (t, J = 7.5 Hz, 2H, CH_2).

^{13}C NMR (125 MHz; $CDCl_3$; Me_4Si), δ: 189.7 (s, CO), 142.1 (t, *J* = 28.5 Hz, *C*CF_2), 141.5 (s, *C*), 139.6 (s, Ph), 128.3 (s, Ph), 128.0 (s, Ph), 126.2 (s, Ph), 118.2 (qt, $^1J_{C-F}$ = 281.0 Hz, $^2J_{C-F}$ = 36.3 Hz, CF_2*C*F_3), 109.5 (tq, $^1J_{C-F}$ = 250.0 Hz, $^2J_{C-F}$ = 38.9 Hz, *C*F_2CF_3), 107.4 (s, *C*H), 41.0 (s, *C*H_2), 29.1 (s, *C*H_2).

^{19}F NMR (375 MHz; $CDCl_3$; $CFCl_3$), δ: -85.1 (s, 3F, CF_2CF_3), -113.6 (s, CF_2CF_3).

MS (CI): *m/z* (%) = 319 $[M+1]^+$.

Anal. calcd for $C_{13}H_9F_5N_2O$: C, 52.84; H, 3.48; N, 8.80. Found: C, 52.51; H, 3.12; N, 8.92.

5-(Diphenylphosphoryl)-3-(pentafluoroethyl)-1*H*-pyrazole (9a)

The synthesis was performed following the general procedure. During the reaction the product partially precipitated from the reaction mixture. After 3 days water (1.0 mL) and CH_2Cl_2 (10 mL) were added. Water phase was separated and discarded. The organic suspension was evaporated under vacuum to afford the pure product **9a** as a white solid (59 mg, 95%). M.p. > 200 ºC.

^{1}H NMR (500 MHz; DMSO-d6; Me_4Si), δ: 14.81 (broad s, NH), 7.67 (broad s, 6H, *Ph*+*Ph*), 7.59 (broad s, 4H, *Ph*+*Ph*), 6.82 (s, 1H, C*H*).

^{13}C NMR (125 MHz; DMSO-d6; Me_4Si), δ: 140.0 (broad s, *C*), the signal N*C*P is not seen, 132.7 (d, ^{4}J(C,P) = 2.5 Hz, *C*H, Ph), 131.4 (d, ^1J(C,P) = 97.5 Hz, *C*, Ph), 131.2 (d, ^{3}J(C,P) = 11.3 Hz, *C*H,

Ph), 128.9 (d, ^{2}J(C,P) = 12.5 Hz, *C*H, Ph), 118.5 (qt, $^1J_{C-F}$ = 285.0 Hz, $^2J_{C-F}$ = 36.3 Hz, $CF_2\mathit{C}F_3$), 112.0 (d, ^{2}J(C,P) = 16.3 Hz, *C*H), 110.6 (tq, $^1J_{C-F}$ = 250.0 Hz, $^2J_{C-F}$ = 38.9 Hz, $\mathit{C}F_2CF_3$).

^{19}F NMR (375 MHz DMSO-d6; $CFCl_3$), δ: -83.7 (broad s, 3F, $CF_2C\mathit{F}_3$), -110.6 (broad s, 2F, $C\mathit{F}_2CF_3$).

^{31}P NMR (202 MHz DMSO-d6; H_3PO_4), δ: 8.8 (broad s, P).

MS (CI): *m/z* (%) = 387 $[M+1]^+$.

Anal. calcd for $C_{17}H_{12}F_5N_2OP$: C, 52.86; H, 3.13; N, 7.25. Found: C, 53.02; H, 3.41; N, 7.02.

2-[3-(pentafluoroethyl)-1*H*-pyrazol-5-yl]-1,3-benzothiazole (10a)

The reaction was performed in in toluene (4.0 mL) /water (0.2 mL) at 45 ºC following the general procedure. The crude product was crystallized from hexane (0.5 mL) to give the pure compound **10a** as a grey solid (24 mg, 45%). M.p. = 142-143 ºC.

^{1}H NMR (500 MHz; $CDCl_3$; Me_4Si), δ: 12.07 (broad s, 1H, N*H*), 8.11 (d, *J* = 8.0 Hz, 1H, C*H*), 7.96 (d, *J* = 8.0 Hz, 1H, C*H*), 7.56 (t, *J* = 8.0 Hz, 1H, C*H*), 7.48 (t, *J* = 8.0 Hz, 1H, C*H*), 7.13 (s, 1H, C*H*).

^{13}C NMR (125 MHz; $CDCl_3$; Me_4Si), δ: 152.7 (s, N*C*S), 139.0 (broad s, *C*), 134.2 (s, *C*H), 126.6 (s, *C*H), 125.8 (s, *C*H), 123.1 (s, *C*H), 121.5 (s, *C*H), *C_2F_5 is not seen*, 105.4 (s, *C*H).

^{19}F NMR (375 MHz; $CDCl_3$; $CFCl_3$), δ: -84.6 (s, 3F, $CF_2C\mathit{F}_3$), -113.4 (s, 2F, $C\mathit{F}_2CF_3$).

MS (CI): *m/z* (%) = 320 $[M+1]^+$.

Anal. calcd for $C_{12}H_6F_5N_3S$: C, 45.15; H, 1.89; N, 13.16; S, 10.04. Found: C, 45.47; H, 2.01; N, 12.88; S, 9.77.

2-[3-(Pentafluoroethyl)-1*H*-pyrazol-5-yl]-thiazole (11a)

The reaction was performed in dichloromethane/water at 40 ºC for 72 h following the general procedure. The crude product was washed with cyclohexane (0.2 mL) to give the pure compound **11a** as a grey solid (24 mg, 57%). M.p. = 127-128 ºC.

^{1}H NMR (500 MHz; DMSO-d_6; Me_4Si), δ: 14.77 (broad s, 1H, N*H*), 8.01 (s, 1H, C*H*), 7.92 (s, 1H, C*H*), 7.34 (s, 1H, C*H*).

^{13}C NMR (125 MHz; DMSO-d_6; Me_4Si), δ: 155.2 (broad s, N*C*S), 143.7 (s, *C*H), 140.5 (broad s, *C*), 138.4 (broad s, *C*), 121.9 (s, *C*H), 104.4 (s, *C*H), *C_2F_5 is not seen*.

^{19}F NMR (375 MHz; DMSO-d_6; $CFCl_3$), δ: -83.2 (s, 3F, $CF_2C\mathit{F}_3$), -110.6 (s, 2F, $C\mathit{F}_2CF_3$).

MS (CI): *m/z* (%) = 270 $[M+1]^+$.

Anal. calcd for $C_8H_4F_5N_3S$: C, 35.69; H, 1.50; N, 15.61; S, 11.91. Found: C, 35.21; H, 1.38; N, 15.29; S, 12.08.

2-[3-((Pentafluoroethyl)-1*H*-pyrazol-5-yl]- quinoxaline (12a)

The reaction was performed in dichloromethane/water at 40 ºC for 72 h following the general procedure. The formed white precipitate was filtered, and dried on air to give the pure product **12a** as a white solid (18 mg, 38%). M.p. > 200 ºC.

^{1}H NMR (500 MHz; DMSO-d_6; Me_4Si), δ: 15.10-14.65 (broad s, 1H, N*H*), 9.56 (s, 1H, C*H*), 8.14 (d, *J* = 8.0 Hz, 2H, C*H*), 7.91 (m, 2H, C*H*), 7.99 (s, 1H, C*H*).

^{13}C NMR (125 MHz; DMSO-d_6; Me_4Si), δ: 143.7 (s), 142.5 (s), 141.5 (s), 141.1 (s), 131.2 (s), 130.6 (s), 129.2 (s), 128.9 (s), 105.6 (s), *two tert-C atoms and C_2F_5 are not seen*.

^{19}F NMR (375 MHz; DMSO-d_6; $CFCl_3$), δ: -83.1 (s, 3F, CF_2CF_3), -110.5 (s, 2F, CF_2CF_3).

MS (CI): *m/z* (%) = 315 $[M+1]^+$.

Anal. calcd for $C_{13}H_7F_5N_4$: C, 49.69; H, 2.25; N, 17.83. Found: C, 49.33; H, 2.53; N, 17.99.

Dimethyl 3-(pentafluoroethyl)-1*H*-pyrazole-4,5-dicarboxylate (15a)

Compound **15a** was obtained as a white solid (48 mg, 99%) following the general procedure. M.p. = 59-60 ºC.

^1H NMR (500 MHz; $CDCl_3$; Me_4Si), δ: 3.97 (s, 3H, CH_3), 3.95 (s, 3H, CH_3).

^{13}C NMR (125 MHz; $CDCl_3$; Me_4Si), δ: 162.0 (s, CO), 158.0 (s, CO), 139.9 (t, J = 28.7 Hz, CCF_2), 134.1 (s, *C*), 118.4 (qt, $^1J_{C-F}$ = 285.0 Hz, $^2J_{C-F}$ = 36.3 Hz, CF_2CF_3), 117.1 (s, *C*), 110.1 (tq, $^1J_{C-F}$ = 250.0 Hz, $^2J_{C-F}$ = 38.9 Hz, CF_2CF_3), 53.3 (s, CH_3), 53.2 (s, CH_3).

^{19}F NMR (375 MHz; $CDCl_3$; $CFCl_3$), δ: -84.3 (t, ^3J(F,F) = 3.8 Hz, 3F, CF_2CF_3), -112.3 (q, ^3J(F,F) = 3.8 Hz, 2F, CF_2CF_3).

MS (CI): *m/z* (%) = 303 $[M+1]^+$.

Anal. calcd for $C_9H_7F_5N_2O_4$: C, 35.78; H, 2.34; N, 9.27. Found: C, 35.49; H, 2.57; N, 9.10.

Diethyl 3-(pentafluoroethyl)-1*H*-pyrazole-4,5-dicarboxylate (16a)

Compound **16a** was obtained as a colorless oil (52 mg, 98%) following the general procedure.

^1H NMR (500 MHz; $CDCl_3$; Me_4Si), δ: 12.05 (broad s, 1H, N*H*), 4.41 (m, 4H, CH_2+CH_2), 1.37 (m, 6H, CH_3+CH_3).

^{13}C NMR (125 MHz; $CDCl_3$; Me_4Si), δ: 161.2 (s, CO), 157.2 (s, CO), 139.4 (t, J = 28.7 Hz, CCF_2), 133.7 (s, *C*), 117.9 (qt, $^1J_{C-F}$ = 285.0 Hz, $^2J_{C-F}$ = 36.3 Hz, CF_2CF_3), 117.1 (s, *C*), 109.9 (tq, $^1J_{C-F}$ = 250.0 Hz, $^2J_{C-F}$ = 38.9 Hz, CF_2CF_3), 62.3 (s, OCH_2), 62.0 (s, OCH_2), 13.5 (s, CH_3), 13.4 (s, CH_3).

^{19}F NMR (375 MHz; $CDCl_3$; $CFCl_3$), δ: -84.3 (s, 3F, CF_2CF_3), -112.2 (s, 2F, CF_2CF_3).

MS (CI): *m/z* (%) = 331 $[M+1]^+$.

Anal. calcd for $C_{11}H_{11}F_5N_2O_4$: C, 40.01; H, 3.26; N, 8.48. Found: C, 39.88; H, 3.59; N, 8.35.

3-(Pentafluoroethyl)-4,5-bis(trifluoromethyl)- 1*H*-pyrazole (17a)

A solution of *bis*-trifluoromethylacethylene (**17**) in dry CH_2Cl_2 was prepared at -30 ˚C (Flask 1). An aliquot of a solution of **17** (100 mg, 0.62 mmol) in dry CH_2Cl_2 (ca. 5 mL) was added to a previously generated solution (flask 2) of $C_2F_5CHN_2$ (obtained from $C_2F_5CH_2NH_2$•HCl (115 mg, 0.62 mmol, 1.0 eq)) in dichloromethane at -10 ˚C. The reaction mixture was allowed to warm slowly to a room temperature and was left for 12 h. The organic layer was separated, dried over sodium sulfate and gently concentrated under vacuum (20 mm) without external heating to give pyrazole **17a** (129 mg, 0.40 mmol, 65% yield) as a yellowish liquid. *The product is volatile!*

^{13}C NMR (125 MHz; DMSO-d_6; Me_4Si), δ: 137.8-136.0 (broad s, CF_3-*C*=N+C_2F_5-*C*=N), 119.4 (q, $^1J_{C-F}$ = 267.5 Hz, CF_3), 118.2 (q, $^1J_{C-F}$ = 268.8 Hz, CF_3), 117.7 (qt, $^1J_{C-F}$ = 285.0 Hz, $^2J_{C-F}$ = 36.3 Hz, CF_2CF_3), 112.3 (q, $^2J_{C-F}$ = 41.3 Hz, CCF_3), 109.0 (tq, $^1J_{C-F}$ = 250.0 Hz, $^2J_{C-F}$ = 38.9 Hz, CF_2CF_3).

^{19}F NMR (375 MHz; DMSO-d_6; $CFCl_3$), δ: -55.7 (s, 3F, CF_3), -61.4 (s, 3F, CF_3), -84.2 (s, 3F, CF_2CF_3), -111.7 (s, 2F, CF_2CF_3).

MS (CI): *m/z* (%) = 321 $[M-1]^+$.

Anal. calcd for $C_7HF_{11}N_2$: C, 26.10; H, 0.31; N, 8.70. Found: C, 26.47; H, 0.65; N, 9.03.

Ethyl 3-(pentafluoroethyl)-4-(trifluoromethyl)-1*H*-pyrazole-5-carboxylate (18a),

Ethyl 3-(pentafluoroethyl)-5-(trifluoromethyl)-1*H*-pyrazole-4-carboxylate (18b)

Compound **18** was obtained as two inseparable regioisomers (2.6/1) as colorless oil (51 mg, 99%) following the general procedure.

^{1}H NMR (500 MHz; $CDCl_3$; Me_4Si), δ: 4.49, 4.39 (2 q, J = 7.0 Hz, OCH_2), 1.43, 1.37 (2 t, J = 7.0 Hz, CH_3).

^{13}C NMR (125 MHz; $CDCl_3$; Me_4Si; signals of major isomer **18a**), δ: 156.7 (s, CO) 139.8 (t, J = 31.5 Hz, *C*CF_2), 134.8 (s, *C*), 120.0 (q, $^{1}J_{C-F}$ = 267.5 Hz, *C*F_3), CF_2*C*F_3 and *C*CF_3 are not seen, 109.9 (tq, $^{1}J_{C-F}$ = 250.0 Hz, $^{2}J_{C-F}$ = 38.9 Hz, *C*F_2CF_3), 62.9 (s, O*C*H_2), 13.4 (s, *C*H_3).

^{19}F NMR (375 MHz; $CDCl_3$; $CFCl_3$), δ: -55.1, -61.6 (2 s, 3F, CF_3), -83.1, -83.6 (2 s, 3F, CF_2CF_3), -109.5, -111.0 (2 s, 2F, CF_2CF_3).

MS (CI): *m/z* (%) = 327 $[M+1]^+$.

Anal. calcd for $C_9H_6F_8N_2O_2$: C, 33.14; H, 1.85; N, 8.59. Found: C, 33.45; H, 1.98; N, 8.31.

1-[3-(Pentafluoroethyl)-5-(trimethylsilyl)-1*H*-pyrazol-4-yl]ethanone (19b)

The reaction was performed at 40 ºC following the general procedure. The crude product was washed with cold cyclohexane (0.1 mL) to give the pure compound **19b** as a white solid (35 mg, 73%). M.p. = 102-103 ºC.

^{1}H NMR (500 MHz; $CDCl_3$; Me_4Si), δ: 10.51 (broad s, 1H, N*H*), 2.56 (s, 3H, CH_3), 0.38 (s, 9H, Si$(CH_3)_3$).

^{13}C NMR (125 MHz; $CDCl_3$; Me_4Si), δ: 193.4 (s, *C*O), 150.4 (s, *C*), 139.5 (t, J = 28.7 Hz, *C*CF_2), 128.8 (s, *C*), 118.2 (qt, $^{1}J_{C-F}$ = 285.0 Hz, $^{2}J_{C-F}$ = 36.3 Hz, CF_2*C*F_3), 111.1 (tq, $^{1}J_{C-F}$ = 250.0 Hz, $^{2}J_{C-F}$ = 38.9 Hz, *C*F_2CF_3), 29.9 (t, $^{5}J_{CF}$ = 7.5 Hz, *C*H_3), -2.3 (s, Si$(CH_3)_3$).

^{19}F NMR (375 MHz; $CDCl_3$; $CFCl_3$), δ: -82.4 (s, 3F, CF_2CF_3), -104.9 (s, 2F, CF_2CF_3).

MS (CI): *m/z* (%) = 301 $[M+1]^+$.
Anal. calcd for $C_{10}H_{13}F_5N_2OSi$: C, 40.00; H, 4.36; N, 9.33. Found: C, 39.76; H, 4.45; N, 9.61.

2-Chloro-1-[3-(pentafluoroethyl)-5-(trimethylsilyl)-1*H*-pyrazol-4-yl]-ethanone (20b)

The reaction was performed at 40 ºC following the general procedure. The crude product (a 4/1 mixture of diastereomers **20b/a**) was washed with cold hexane (0.2 mL) to give the pure compound **20b** as a white solid (29 mg, 55%). M.p. = 107-108 ºC.

^{1}H NMR (500 MHz; $CDCl_3$; Me_4Si), δ: 10.60 (broad s, 1H, N*H*), 4.68 (s, 2H, CH_2), 0.40 (s, 9H, Si$(CH_3)_3$).

^{13}C NMR (125 MHz; $CDCl_3$; Me_4Si), δ: 187.1 (s, *C*O), 151.7 (s, *C*), 139.2 (t, J = 31.5 Hz, *C*CF_2), 125.6 (s, *C*), *C_2F_5 is not seen*, 48.3 (t, $^{5}J_{CF}$ = 7.5 Hz, *C*H_2), -2.5 (s, Si$(CH_3)_3$).

^{19}F NMR (375 MHz; $CDCl_3$; $CFCl_3$), δ: -82.4 (s, 3F, CF_2CF_3), -105.2 (s, 2F, CF_2CF_3).

MS (CI): *m/z* (%) = 336 $[M+1]^+$.
Anal. calcd for $C_{10}H_{12}ClF_5N_2OSi$: C, 35.88; H, 3.61; N, 8.37. Found: C, 36.16; H, 3.33; N, 8.05.

2,2-Difluoro-1-[3-(pentafluoroethyl)-4-(trimethylsilyl)-1*H*-pyrazol-5-yl]-ethanone (21a),
2,2-Difluoro-1-[3-(pentafluoroethyl)-5-(trimethylsilyl)-1*H*-pyrazol-4-yl]-ethanone (21b)

Compound **21b/a** was obtained as two inseparable regioisomers (2.6/1) as colorless oil (48 mg, 89%) following the general procedure.

^{1}H NMR (500 MHz; $CDCl_3$; Me_4Si), δ: 10.79 (broad s, 1H, N*H*), 6.56, 6.32 (2 t, $^{2}J_{HF}$ = 53.6 Hz, 1H, C*H*F_2), 0.38, 0.30 (2 s, 9H, Si(C*H*$_3$)$_3$).

^{13}C NMR (125 MHz; $CDCl_3$; Me_4Si; major isomer **21b**), δ: 185.3 (t, $^{2}J_{C\text{-}F}$ = 26.3 Hz, *C*OCHF_2), 151.8 (s, *C*), 139.9 (t, $^{2}J_{C\text{-}F}$ = 31.3 Hz, *C*CF_2), 123.2 (s, *C*), 118.4 (qt, $^{1}J_{C\text{-}F}$ = 285.0 Hz, $^{2}J_{C\text{-}F}$ = 36.3 Hz, CF_2*C*F_3), 110.4 (tq, $^{1}J_{C\text{-}F}$ = 250.0 Hz, $^{2}J_{C\text{-}F}$ = 38.9 Hz, *C*F_2CF_3), 107.t (tt, $^{1}J_{C\text{-}F}$ = 247.5 Hz, $^{5}J_{C\text{-}F}$ = 7.5 Hz, *C*HF_2), -2.6 (s, Si(*C*H_3)$_3$).

^{19}F NMR (375 MHz; $CDCl_3$; $CFCl_3$), δ: -83.1, -83.8 (s + broad s, 3F, CF_2C*F*$_3$), -107.2, -108.4 (s + broad s, 2F, C*F*$_2CF_3$), -127.3, 127.0 (broad s + dt, $^{2}J_{FH}$ = 52.6 Hz, $^{6}J_{FF}$ = 7.5 Hz, 2F, CH*F*$_2$),

MS (CI): *m/z* (%) = 337 $[M+1]^+$.
Anal. calcd for $C_{10}H_{11}F_7N_2OSi$: C, 35.72; H, 3.30; N, 8.33. Found: C, 35.45; H, 3.13; N, 8.54.

1-[3-(Trifluoromethyl)-5-(trimethylsilyl)-1*H*-pyrazol-4-yl]-ethanone (23)

To a stirred suspension of $CF_3CH_2NH_2$*HCl (65 mg, 0.48 mmol, 3.0 eq.) in CH_2Cl_2 (4.0 mL) / water (0.2 mL), sodium nitrite (54 mg, 0.78 mmol, 5.0 eq.) and alkyne **19** (22 mg, 0.16 mmol, 1.0 eq.) was added. The reaction mixture was vigorously stirred 168 h at RT. Water (1.0 mL) and CH_2Cl_2 (3 mL) were added. The organic layer was separated. The aqueous layer was washed with CH_2Cl_2 (2 × 3 mL). The combined organic layers were dried over Na_2SO_4 and evaporated under vacuum to provide the crude material (*no starting material was left*). The tarry solid was washed with cold cyclohexane (0.1 mL) to give the pure product **23** (30 mg, 0.12 mmol, 75% yield) as a white solid. M.p.= 120-122 °C.
^{1}H NMR (500 MHz; $CDCl_3$; Me_4Si), δ: 11.16 (broad s, 1H, N*H*), 2.57 (s, 3H, C*H*$_3$), 0.39 (s, 9H, Si(C*H*$_3$)$_3$).
^{13}C NMR (125 MHz; $CDCl_3$; Me_4Si), δ: 192.6 (s, *C*O), 151.3 (s, *C*), 141.0 (q, ^{2}J(C,F) = 36.1 Hz, *C*CF_3), 127.1 (s), 120.8 (q, ^{1}J(C,F) = 270.0 Hz, *C*F_3,), 29.2 (s, *C*H_3), 2.6 (s, Si(*C*H_3)$_3$).
^{19}F NMR (375 MHz; $CDCl_3$; $CFCl_3$), δ: -58.1 (s, C*F*$_3$).
Anal. calcd for $C_9H_{13}F_3N_2OSi$: C, 43.19; H, 5.24; N, 11.19. Found: C, 43.42; H, 5.53; N, 10.94.

MS (CI): *m/z* (%) = 251 $[M+1]^+$.

1-[3-(Pentafluoroethyl)-1*H*-pyrazol-4-yl]-ethanone (24)

Compound **19b** (50 mg, 0.17 mmol) was dissolved in methanol (3 mL). Water (0.5 mL), and KHF_2 (5 mg) were added. The reaction mixture was heated at reflux for 10h. After cooling to a room temperature, the methanol was evaporated under vacuum. Water (1 mL) was added and the mixture was extracted with dichloromethane (3×2 mL). The organic fractions were combined, dried over Na_2SO_4 and evaporated under vacuum to afford pyrazole **24** (17 mg, 0.08 mmol, 46% yield) as a white solid. M.p. = 152-153 ºC.

^{1}H NMR (500 MHz; DMSO-d_6; Me_4Si), δ: 8.75 (s, 1H, C*H*), 2.44 (s, 3H, C*H*$_3$).

^{13}C NMR (125 MHz DMSO-d_6; Me_4Si), δ: 190.2 (s, *C*O), 137.9 (t, *J* = 28.7 Hz, *C*CF_2), 136.7 (s), 121.5 (s), 119.1 (qt, $^{1}J_{C\text{-}F}$ = 285.0 Hz, $^{2}J_{C\text{-}F}$ = 36.3 Hz, CF_2*C*F_3), 110.9 (tq, $^{1}J_{C\text{-}F}$ = 250.0 Hz, $^{2}J_{C\text{-}F}$ = 38.9 Hz, *C*F_2CF_3), 29.0 (s, *C*H_3).

^{19}F NMR (375 MHz; DMSO-d_6; $CFCl_3$), δ: -80.9 (s, 3F, CF_2C*F*$_3$), -107.4 (s, 2F, C*F*$_2CF_3$).

MS (CI): *m/z* (%) = 229 $[M+1]^+$.
Anal. calcd for $C_7H_5F_5N_2O$: C, 36.86; H, 2.21; N, 12.38. Found: C, 36.51; H, 2.44; N, 12.17.

3-(Pentafluoroethyl)-1*H*-pyrazole-5-carboxylic acid (25)

To a solution of pyrazole **1a** (93 mg, 0.38 mmol) in MeOH (0.5 mL) was added 1N NaOH (1.6 mL, 1.60 mmol). The reaction mixture was vigorously stirred at room temperature for 20 h. The solvent

was evaporated under vacuum, and water (5 mL) was added. The water phase was washed with CH_2Cl_2 (2 * 1mL). Organic layer was discarded. Water phase was acidified with conc. HCl to pH = 1. White precipitate was formed. The suspension was extracted EtOAc (3 * 5mL). The combined organic layer was dried over sodium sulfate, and evaporated under vacuum to afford the pure acid **25** (71 mg, 0.31 mmol, 81% yield) as a white solid. M.p.= 134-135 °C.

^{1}H NMR (500 MHz; DMSO-d_6; Me_4Si), δ: 14.78 (s, 1H, N*H*), 7.20 (s, 1H, C*H*).

^{13}C NMR (125 MHz; DMSO-d_6; Me_4Si), δ: 159.6 (s, *C*O_2H), 140.2 (t, ^2J(C,F) = 28.8 Hz, *C*CF_2), 136.8 (s, *C*), 118.3 (qt, $^1J_{C\text{-}F}$ = 285.0 Hz, $^2J_{C\text{-}F}$ = 36.3 Hz, CF_2CF_3), 112.4 (tq, $^1J_{C\text{-}F}$ = 250.0 Hz, $^2J_{C\text{-}F}$ = 38.9 Hz, *C*F_2CF_3), 108.1 (s, *C*H).

^{19}F NMR (375 MHz; DMSO-d_6; $CFCl_3$), δ: -85.2 (s, 3F, CF_2CF_3), -113.8 (s, 2F, CF_2CF_3).

MS (CI): *m/z* (%) = 231 $[M+1]^+$.

Anal. calcd for $C_6H_3F_5N_2O_2$: C, 31.32; H, 1.31; N, 12.17. Found: C, 31.63; H, 1.02; N, 12.48.

1-Methyl-3-(pentafluoroethyl)-1*H*-pyrazole-5-carboxylic acid, methyl ester (26)

Pyrazole **1a** (450 mg, 1.8 mmol) was dissolved in dry DMF (5 mL). Dry K_2CO_3 (472 mg, 3.6 mmol), and MeI (523 mg, 3.6 mmol) were added. The reaction mixture was vigorously stirred at room temperature for 20 h. The reaction mixture was than subjected to column chromatography on silicagel using hexane/EtOAc = 4/1 as an eluent. R_f = 0.6. Colorless liquid **26** (328 mg, 1.2 mmol, 69% yield).

^{1}H NMR (500 MHz; $CDCl_3$; Me_4Si), δ: 7.10 (s, 1H, C*H*), 4.25 (s, 3H, C*H*$_3$), 3.92 (s, 3H, C*H*$_3$).

^{13}C NMR (125 MHz; $CDCl_3$; Me_4Si), δ: 158.9 (s, CO), 139.0 (t, ^2J(C,F) = 28.8 Hz, *C*CF_2), 133.5 (s, *C*), 118.3 (qt, $^1J_{C\text{-}F}$ = 285.0 Hz, $^2J_{C\text{-}F}$ = 36.3 Hz, CF_2CF_3), 108.6 (s, *C*H), 110.4 (tq, $^1J_{C\text{-}F}$ = 250.0 Hz, $^2J_{C\text{-}F}$ = 38.9 Hz, *C*F_2CF_3), 52.0 (s, *C*H_3), 40.0 (s, *C*H_3).

^{19}F NMR (375 MHz; $CDCl_3$; $CFCl_3$), δ: -85.2 (s, 3F, CF_2CF_3), -113.8 (s, 2F, CF_2CF_3).

MS (CI): *m/z* (%) = 259 $[M+1]^+$.

Anal. calcd for $C_8H_7F_5N_2O_2$: C, 37.22; H, 2.73; N, 10.85. Found: C, 37.48; H, 2.91; N, 10.56.

1-Methyl-3-(pentafluoroethyl)-1*H*-pyrazole-5-carboxylic acid (27)

To a solution of pyrazole **26** (100 mg, 0.38 mmol) in MeOH (0.5 mL) was added 1N NaOH (0.8 mL, 0.80 mmol). The reaction mixture was vigorously stirred at room temperature for 20 h. The solvent was evaporated under vacuum, and water (5 mL) was added. The water phase was washed with CH_2Cl_2 (2 * 1mL). Organic layer was discarded. Water phase was acidified with conc. HCl to pH = 1. White precipitate was formed. The suspension was extracted CH_2Cl_2 (3 * 5mL). The combined organic layer was dried over sodium sulfate, and evaporated under vacuum to afford the pure acid **27** (92 mg, 0.37 mmol, 97% yield) as a white solid. M.p.= 125-126 °C.

^{1}H NMR (500 MHz; $CDCl_3$; Me_4Si), δ: 7.25 (s, 1H, C*H*), 4.28 (s, 3H, C*H*$_3$).

^{13}C NMR (125 MHz; $CDCl_3$; Me_4Si), δ: 162.4 (s, CO), 139.4 (t, ^2J(C,F) = 28.5 Hz, *C*CF_2), 132.8 (s, *C*), 118.2 (qt, $^1J_{C\text{-}F}$ = 285.0 Hz, $^2J_{C\text{-}F}$ = 36.5 Hz, CF_2CF_3), 109.8 (tq, $^1J_{C\text{-}F}$ = 250.0 Hz, $^2J_{C\text{-}F}$ = 38.9 Hz, *C*F_2CF_3), 112.0 (s, *C*H), 40.0 (s, *C*H_3).

^{19}F NMR (375 MHz; $CDCl_3$; $CFCl_3$), δ: -85.1 (s, 3F, CF_2CF_3), -113.8 (s, 2F, CF_2CF_3).

MS (CI): *m/z* (%) = 243 $[M-1]^+$.

Anal. calcd for $C_7H_5F_5N_2O_2$: C, 34.44; H, 2.06; N, 11.48. Found: C, 34.17; H, 1.91; N, 11.37.

Copies of NMR spectra.

Compound 1a

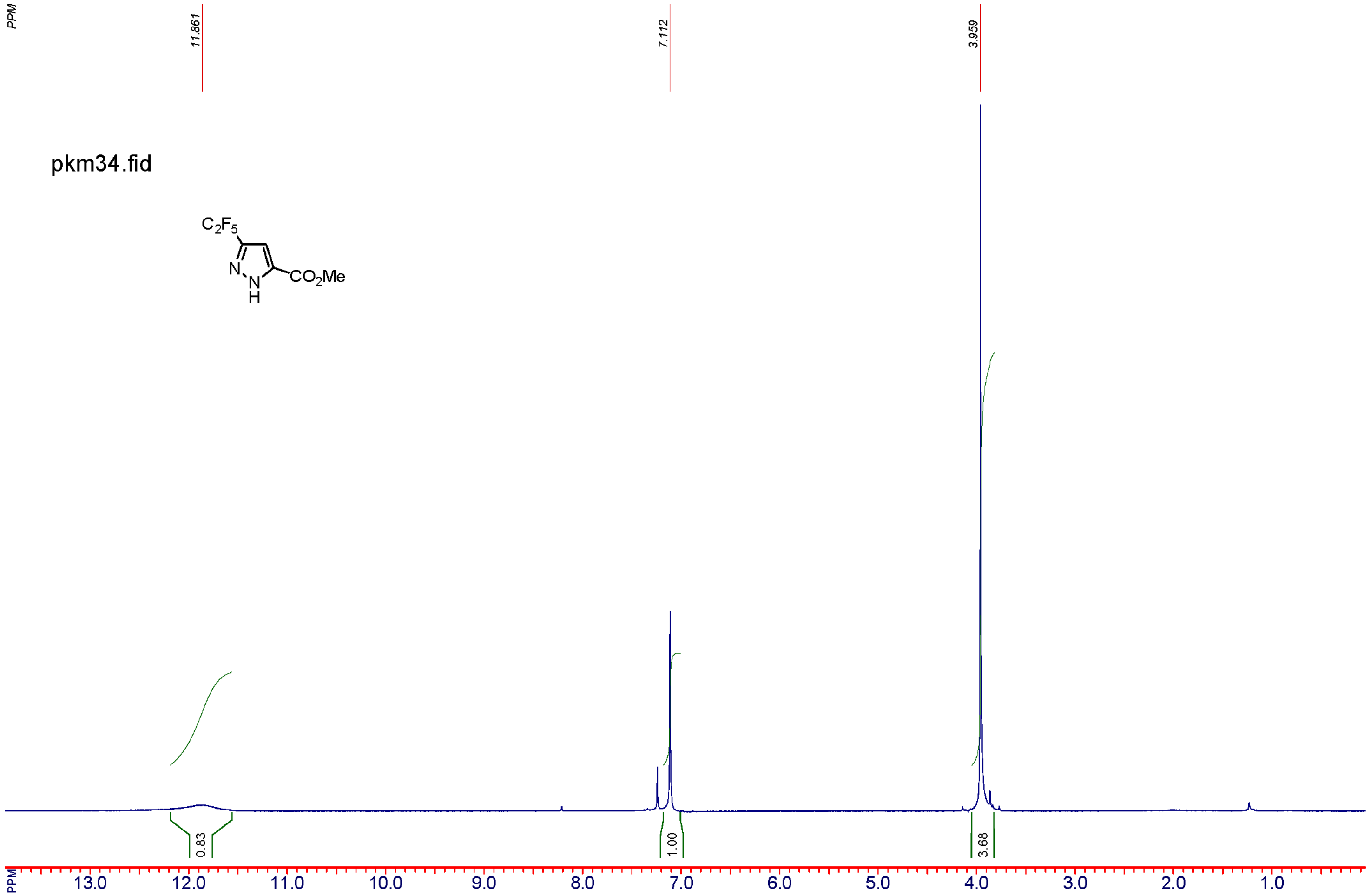

**File name: pkm34.fid**	Operator:	SF: 400.0002 MHz	NSC: 0	PW: 7.50 usec, RG: 26	SI: 32768
Date: 08-Jul-2013	Solvent: CDCl3	SW: 7261 Hz	TE: 293 K	AQ: 1.24 sec, RD: 0.00 sec	

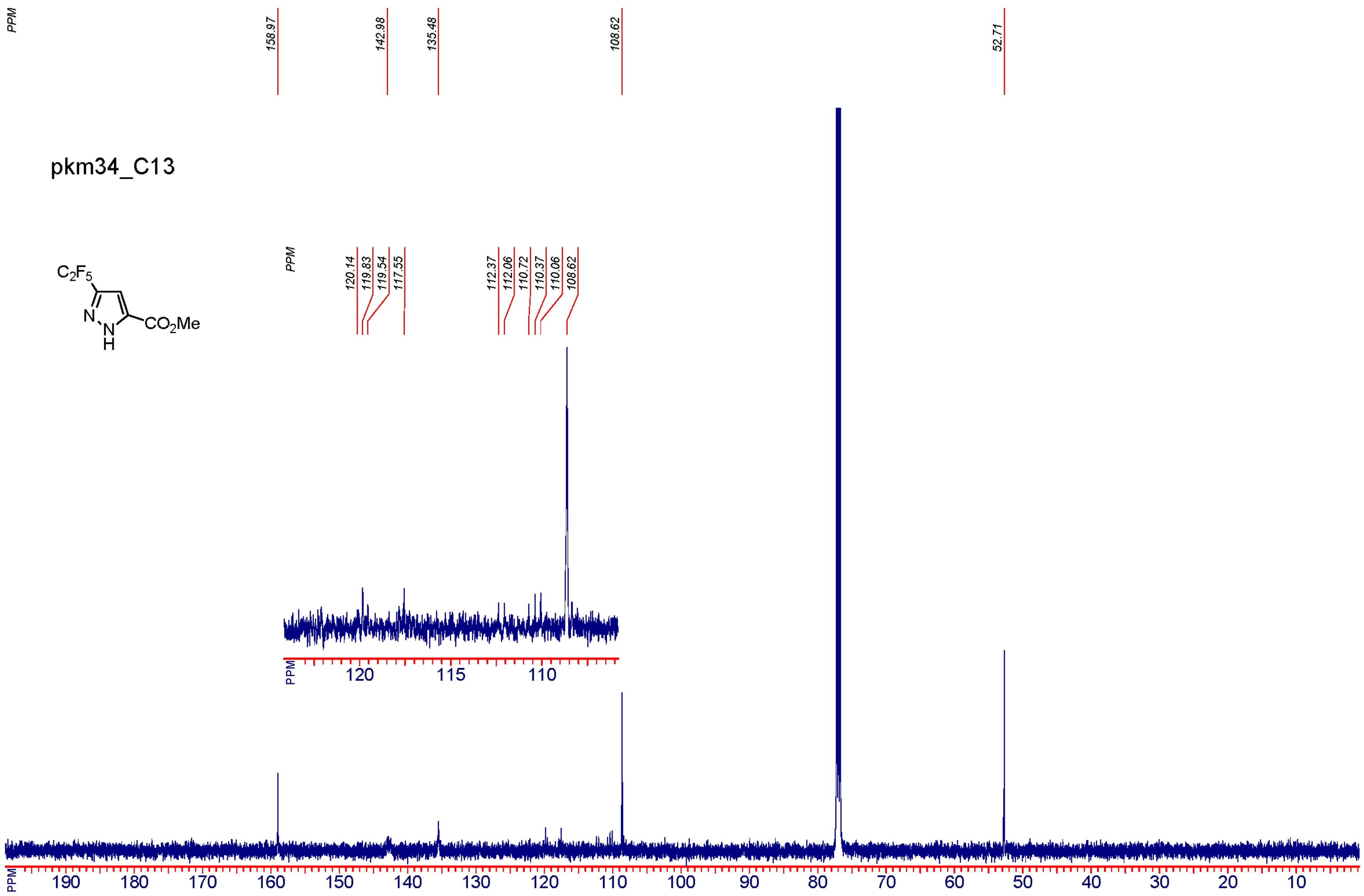

**File name: pkm34_C13**	Operator: root	SF: 125.7422 MHz	NSC: 1125	PW: 0.00 usec, RG: 51200	SI: 65536
Date: 10-Jul-2013	Solvent: dmso	SW: 32680 Hz	TE: 0 K	AQ: 1.57 sec, RD: 0.00 sec	Parameter file, XWIN-NMRVersion 3.5

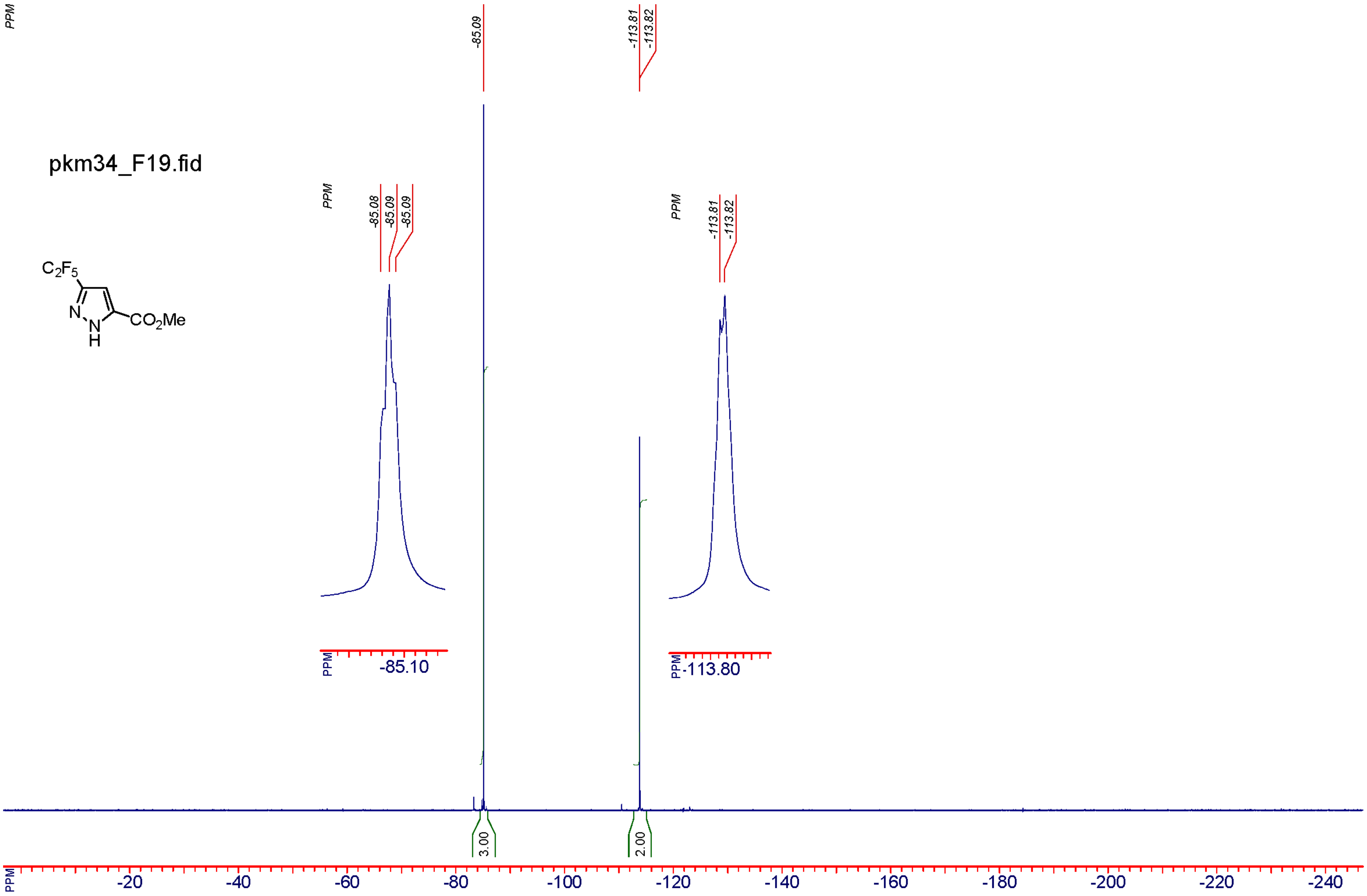

**File name: pkm34_F19.fid**	Operator:	SF: 376.3189 MHz	NSC: 0	PW: 3.00 usec, RG: 16	SI: 131072
Date: 11-Jul-2013	Solvent: cdcl3	SW: 94118 Hz	TE: 293 K	AQ: 0.68 sec, RD: 0.00 sec	

Compound 2a

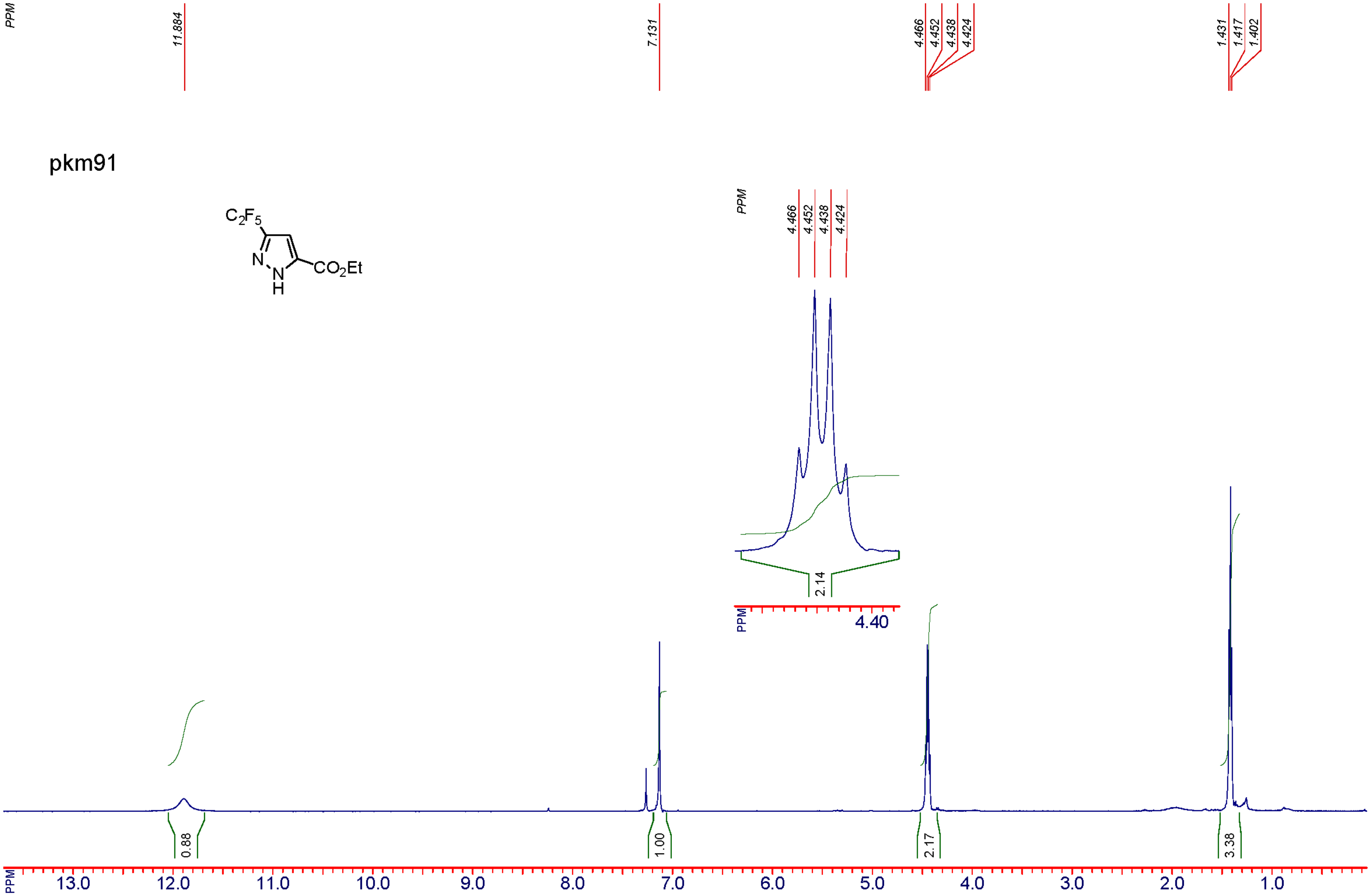

File name: pkm91	Operator: root	SF: 499.9500 MHz	NSC: 1	PW: 0.00 usec, RG: 24	SI: 32768
Date: 21-Oct-2013	Solvent: CDCl3	SW: 8993 Hz	TE: 0 K	AQ: 1.82 sec, RD: 0.00 sec	Parameter file, XWIN-NMRVersion 3.5

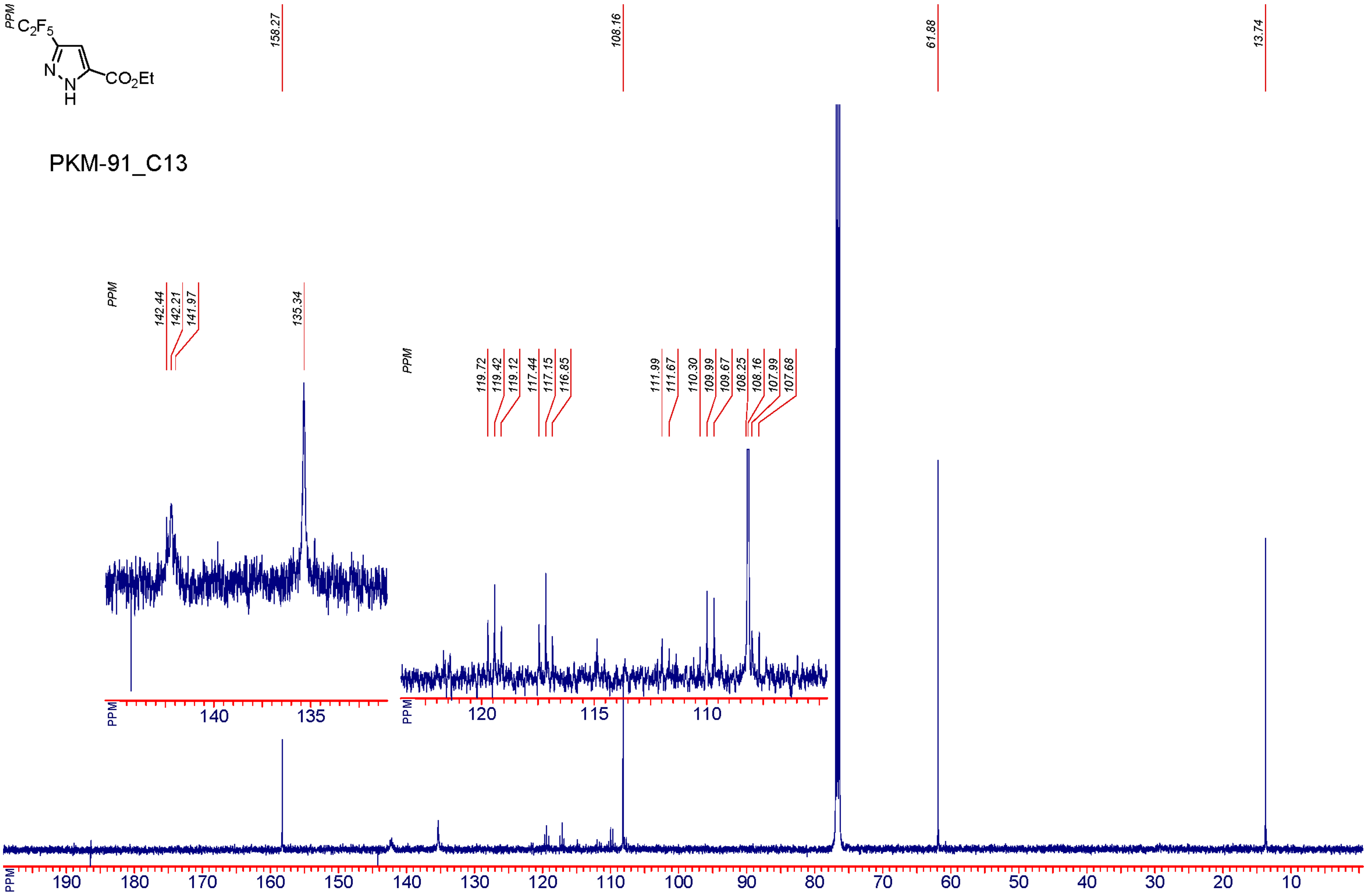

**File name: PKM-91_C13**	Operator: root	SF: 125.7126 MHz	NSC: 2651	PW: 0.00 usec, RG: 51200	SI: 65536
Date: 22-Oct-2013	Solvent: DMSO	SW: 32680 Hz	TE: 0 K	AQ: 1.57 sec, RD: 0.00 sec	Parameter file, XWIN-NMRVersion 3.5

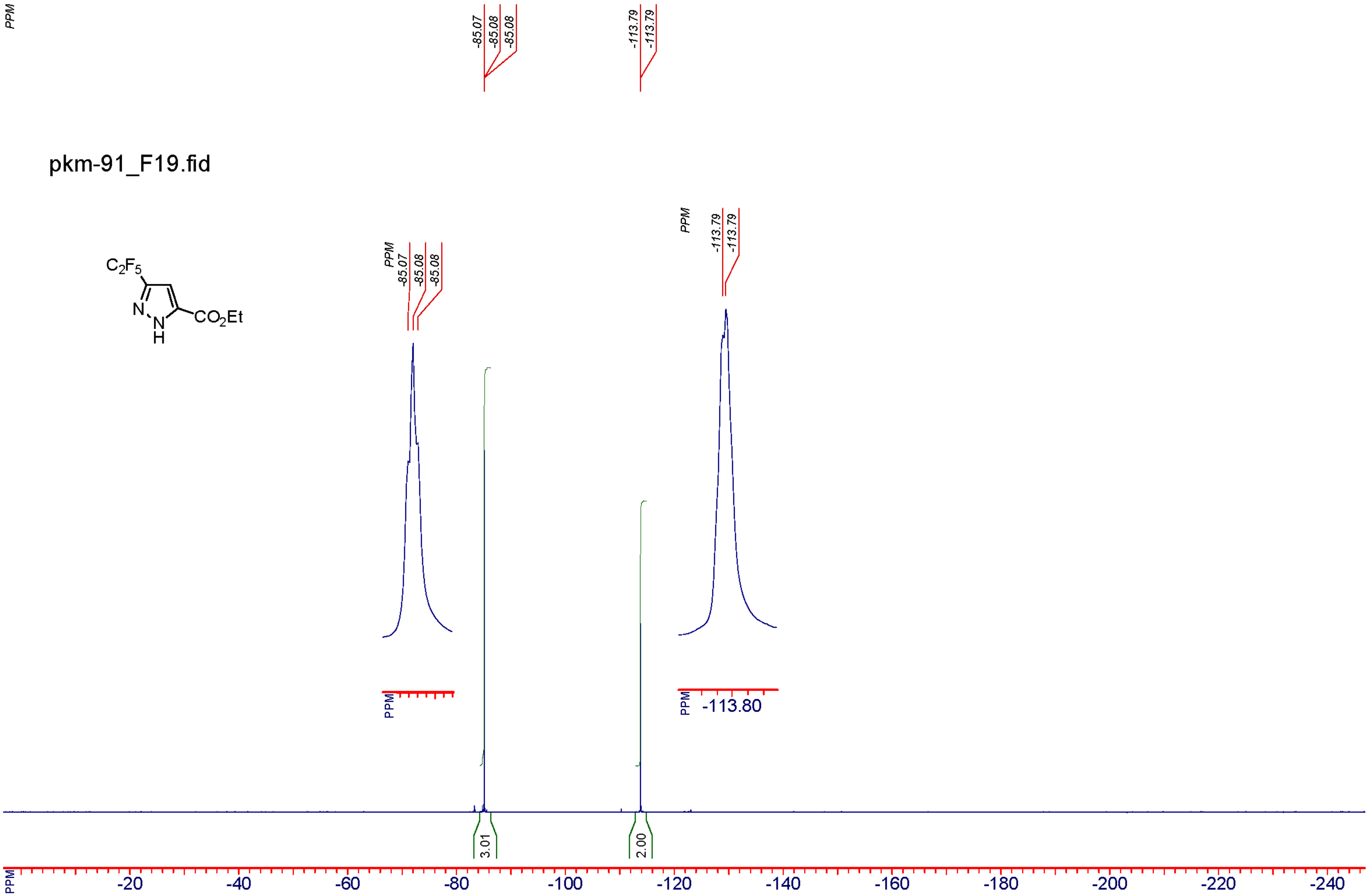

**File name: pkm-91_F19.fid**	Operator:	SF: 376.3189 MHz	NSC: 0	PW: 3.00 usec, RG: 16	SI: 131072
Date: 24-Oct-2013	Solvent: cdcl3	SW: 94118 Hz	TE: 293 K	AQ: 0.68 sec, RD: 0.00 sec	

Compound 3a

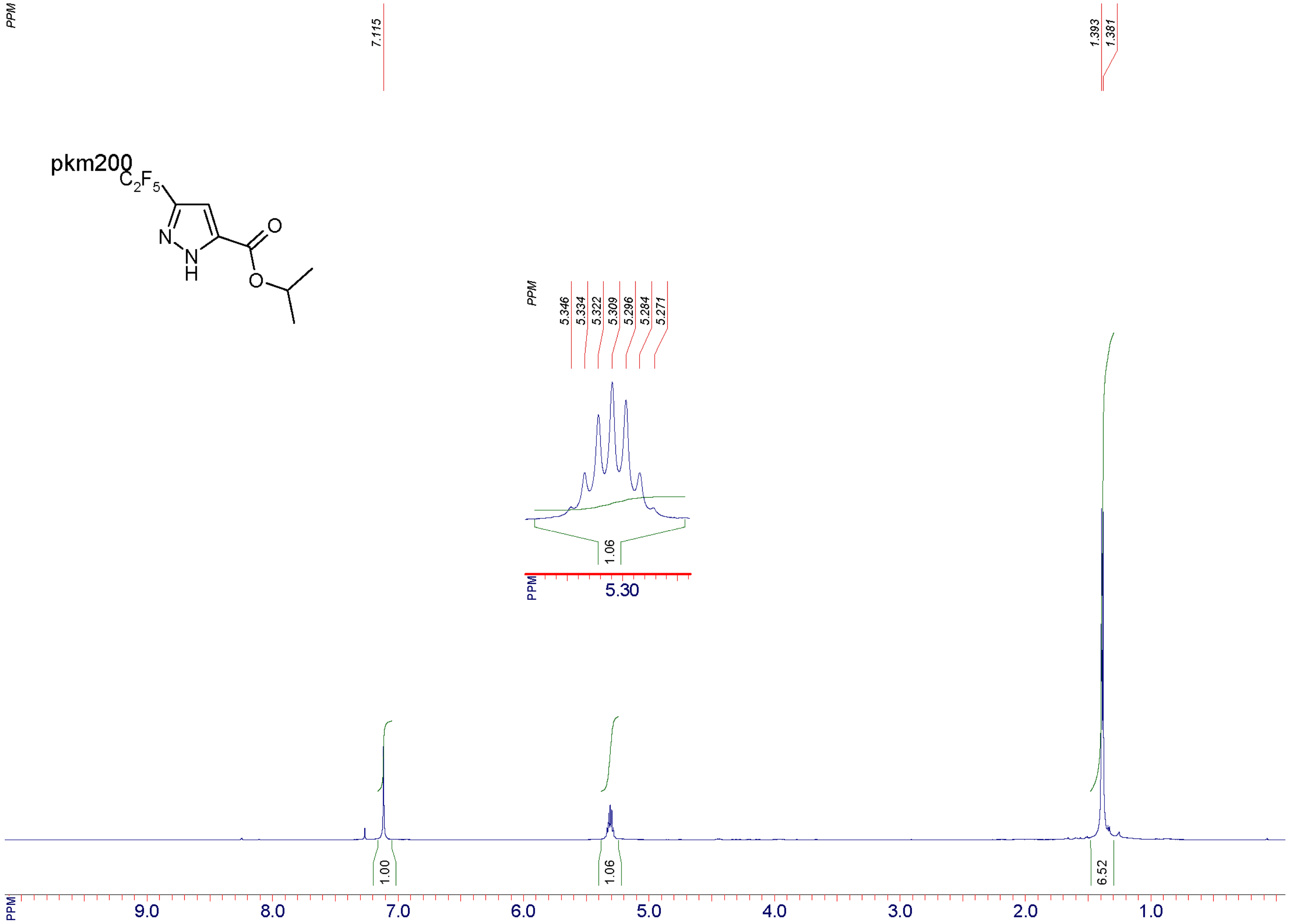

**File name: pkm200**	Operator: root	SF: 499.9500 MHz	NSC: 1	PW: 0.00 usec, RG: 200	SI: 32768
Date: 12-May-2014	Solvent: CDCl3	SW: 8993 Hz	TE: 0 K	AQ: 1.82 sec, RD: 0.00 sec	Parameter file, XWIN-NMR Version 3.5

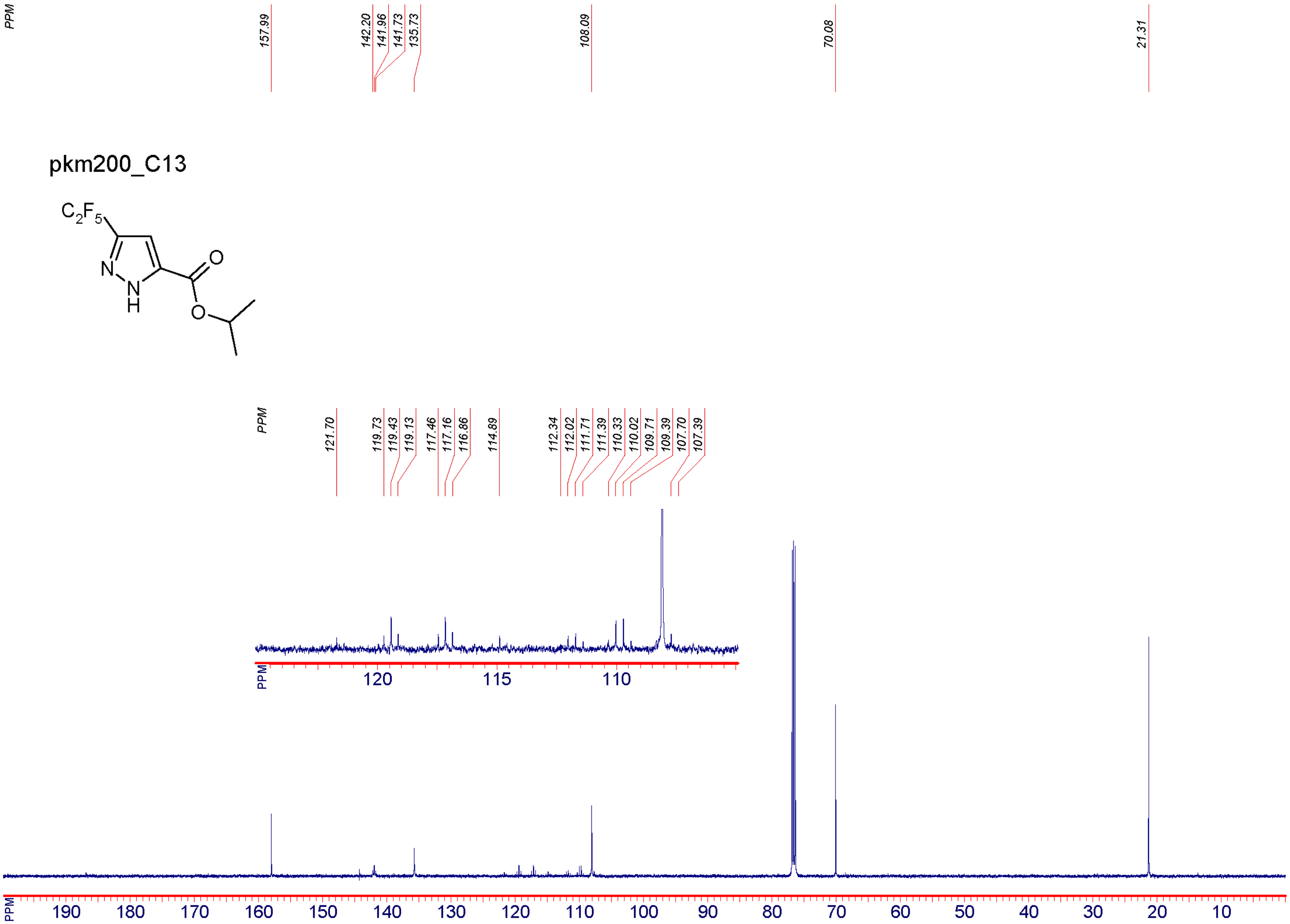

**File name: pkm200_C13**	Operator: root	SF: 125.7126 MHz	NSC: 1619	PW: 0.00 usec, RG: 51200	SI: 65536
Date: 14-May-2014	Solvent: CDCl3	SW: 32680 Hz	TE: 0 K	AQ: 1.57 sec, RD: 0.00 sec	Parameter file, XWIN-NMR Version 3.5

Pkm200_F19.fid

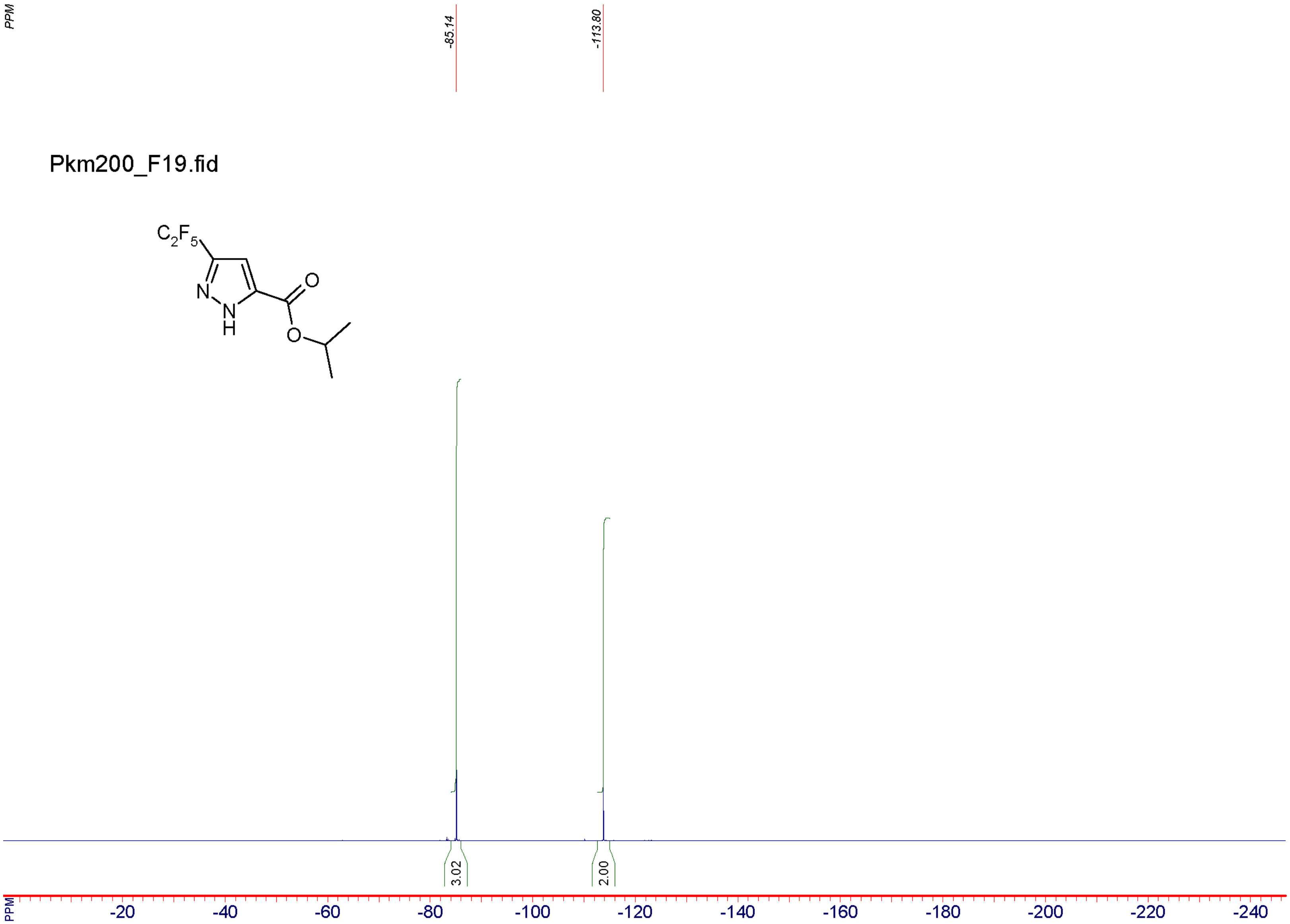

**File name: Pkm200_F19.fid**	Operator:	SF: 376.3189 MHz	NSC: 0	PW: 3.00 usec, RG: 32	SI: 65536
Date: 20-May-2014	Solvent: cdcl3	SW: 94118 Hz	TE: 293 K	AQ: 0.34 sec, RD: 0.00 sec	⊓

Compound 4a

pkm-106

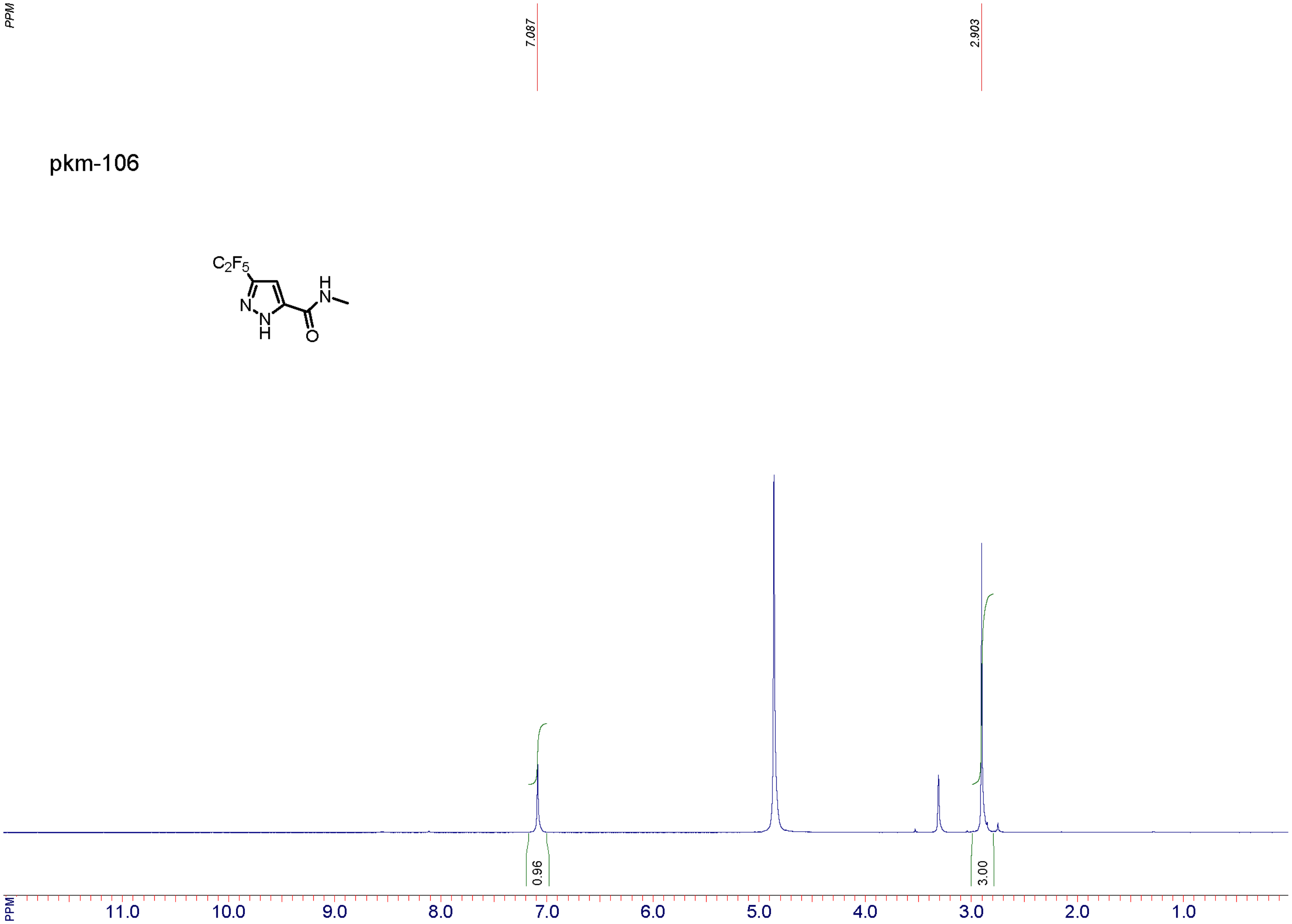

File name: pkm-106	Operator: root	SF: 499.9500 MHz	NSC: 1	PW: 0.00 usec, RG: 32	SI: 32768
Date: 06-Nov-2013	Solvent: MeOD	SW: 8993 Hz	TE: 0 K	AQ: 1.82 sec, RD: 0.00 sec	Parameter file, XWIN-NMR Version 3.5

pkm-106   C13

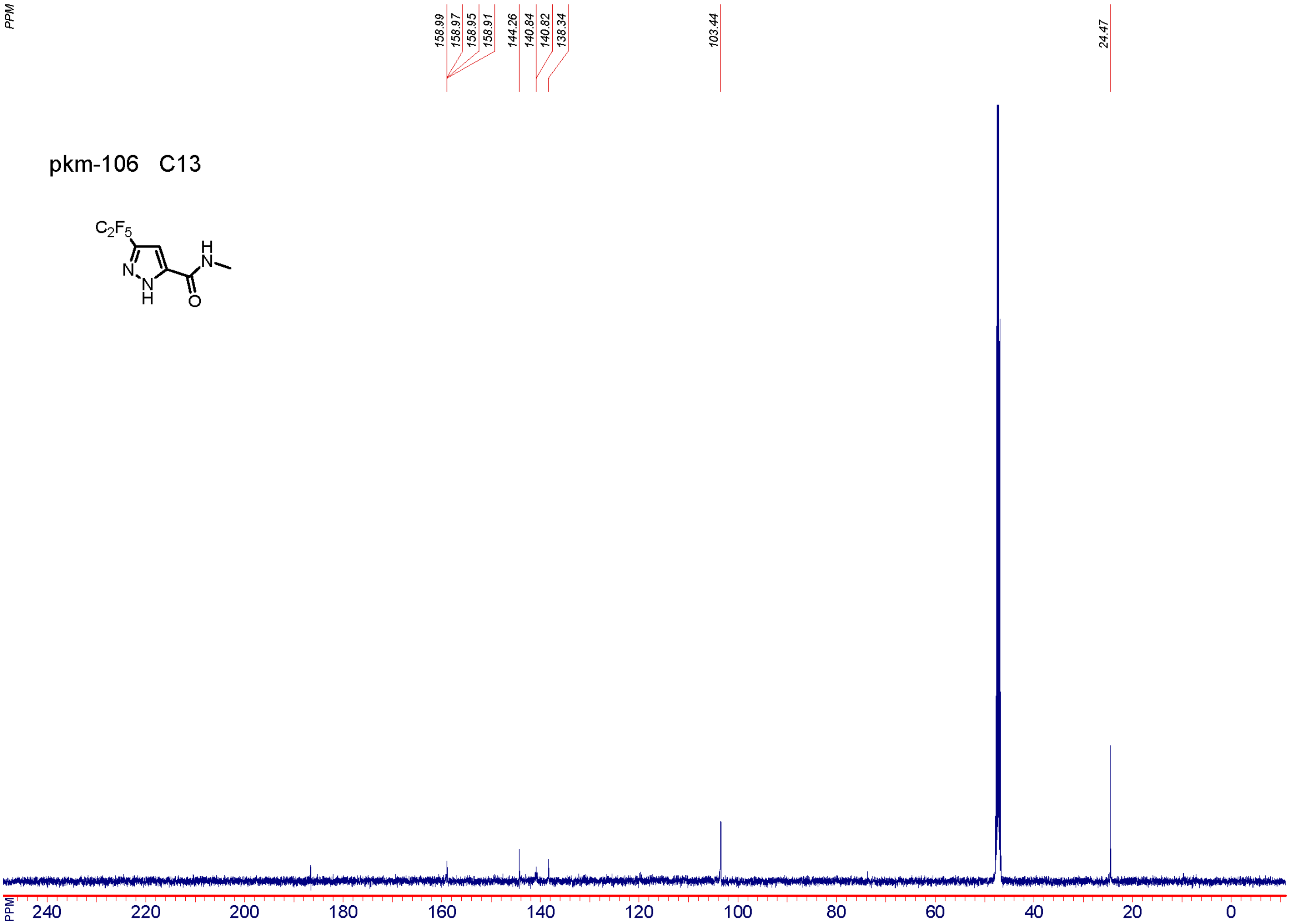

**File name: pkm-106 C13**	Operator: root	SF: 125.7126 MHz	NSC: 9466	PW: 0.00 usec, RG: 51200	SI: 65536
Date: 08-Nov-2013	Solvent: MeOD	SW: 32680 Hz	TE: 0 K	AQ: 1.57 sec, RD: 0.00 sec	Parameter file, XWIN-NMR Version 3.5

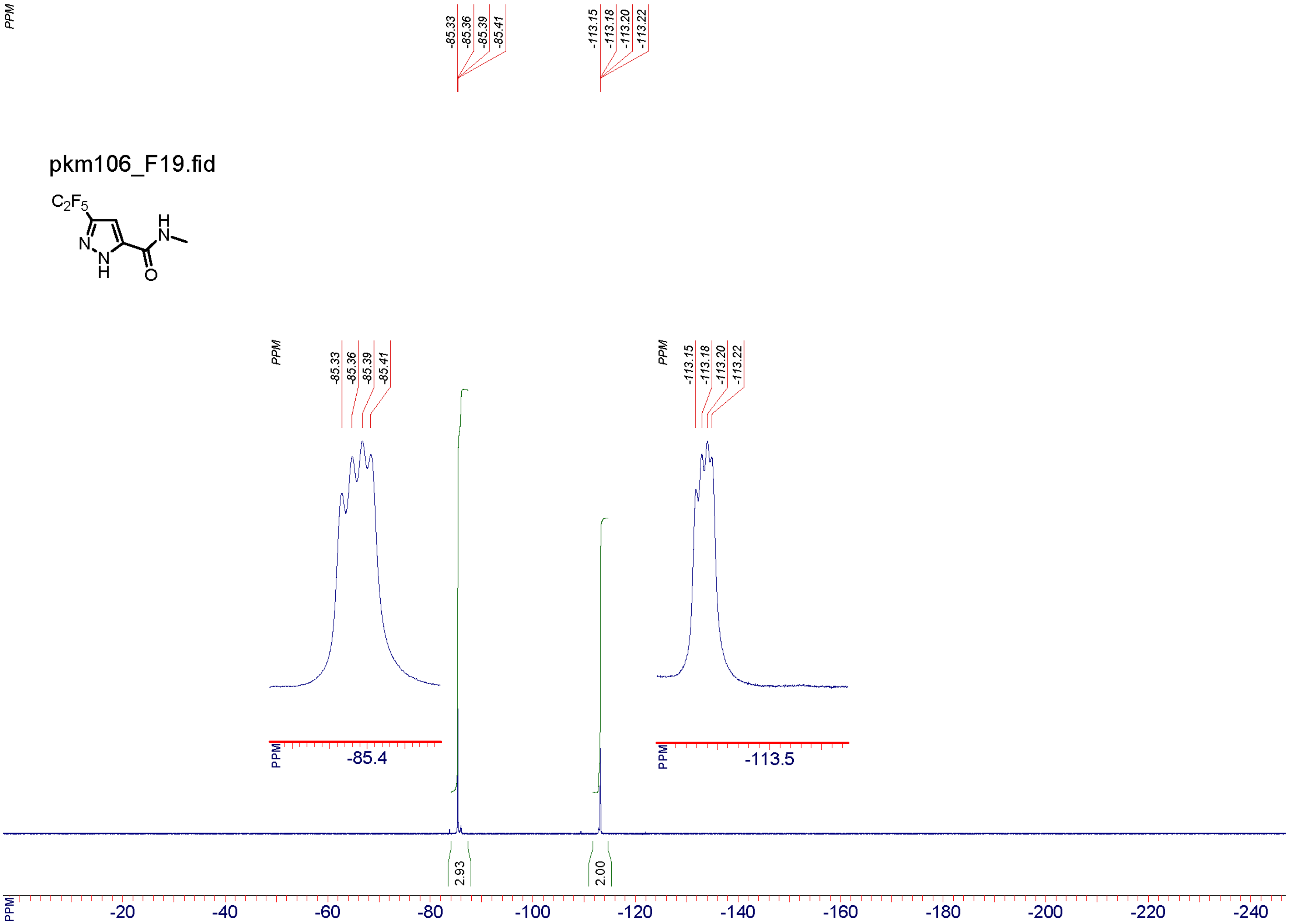

**File name: pkm106_F19.fid**	Operator:	SF: 376.3189 MHz	NSC: 0	PW: 3.00 usec, RG: 16	SI: 131072
Date: 12-Nov-2013	Solvent: cdcl3	SW: 94118 Hz	TE: 293 K	AQ: 0.68 sec, RD: 0.00 sec	⊓

Compound 5a

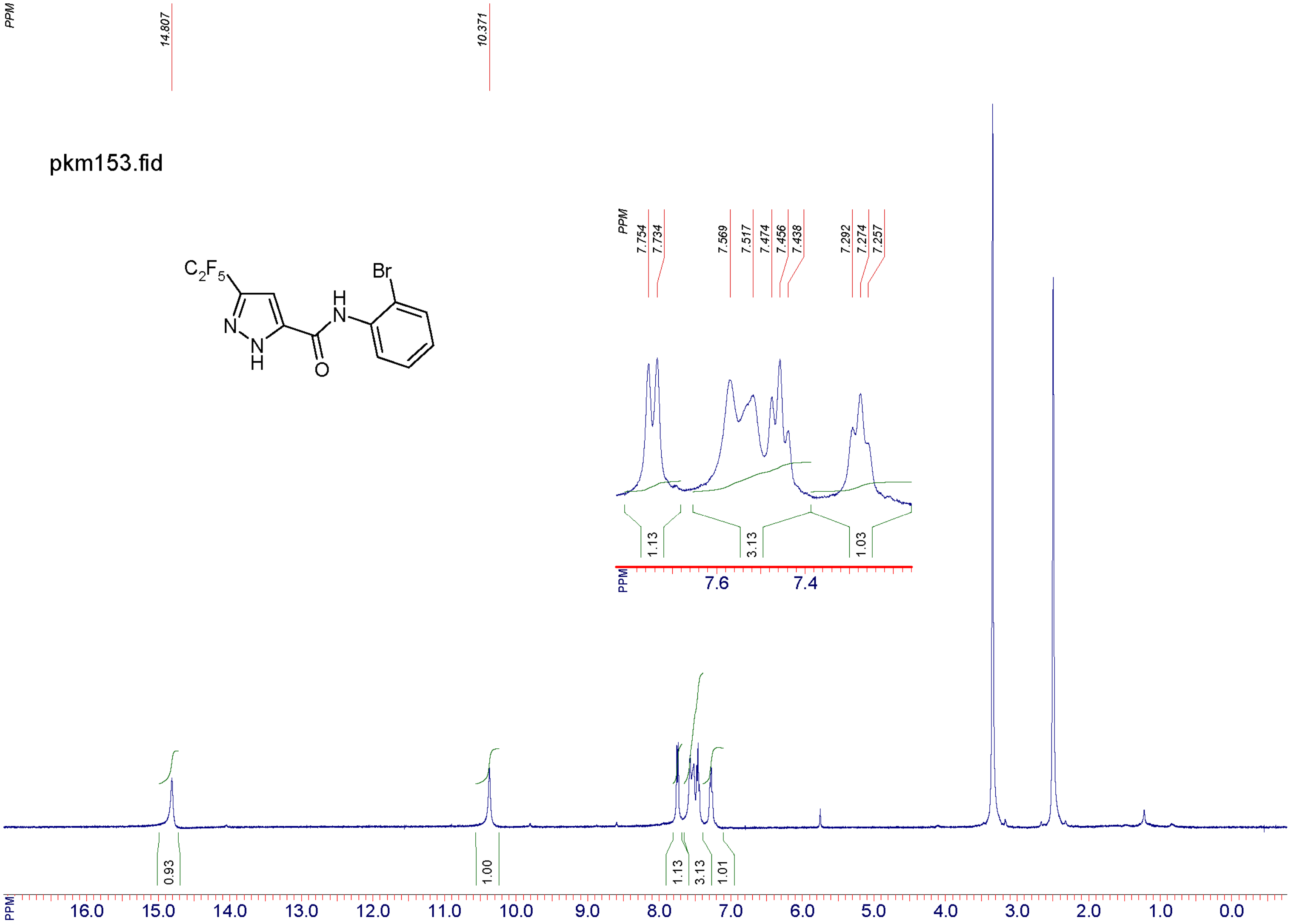

**File name: pkm153.fid**	Operator:	SF: 400.0019 MHz	NSC: 0	PW: 7.50 usec, RG: 26	SI: 32768
Date: 31-Jan-2014	Solvent: dmso_d6	SW: 7175 Hz	TE: 293 K	AQ: 1.67 sec, RD: 0.00 sec	⊓

pkm153_C13

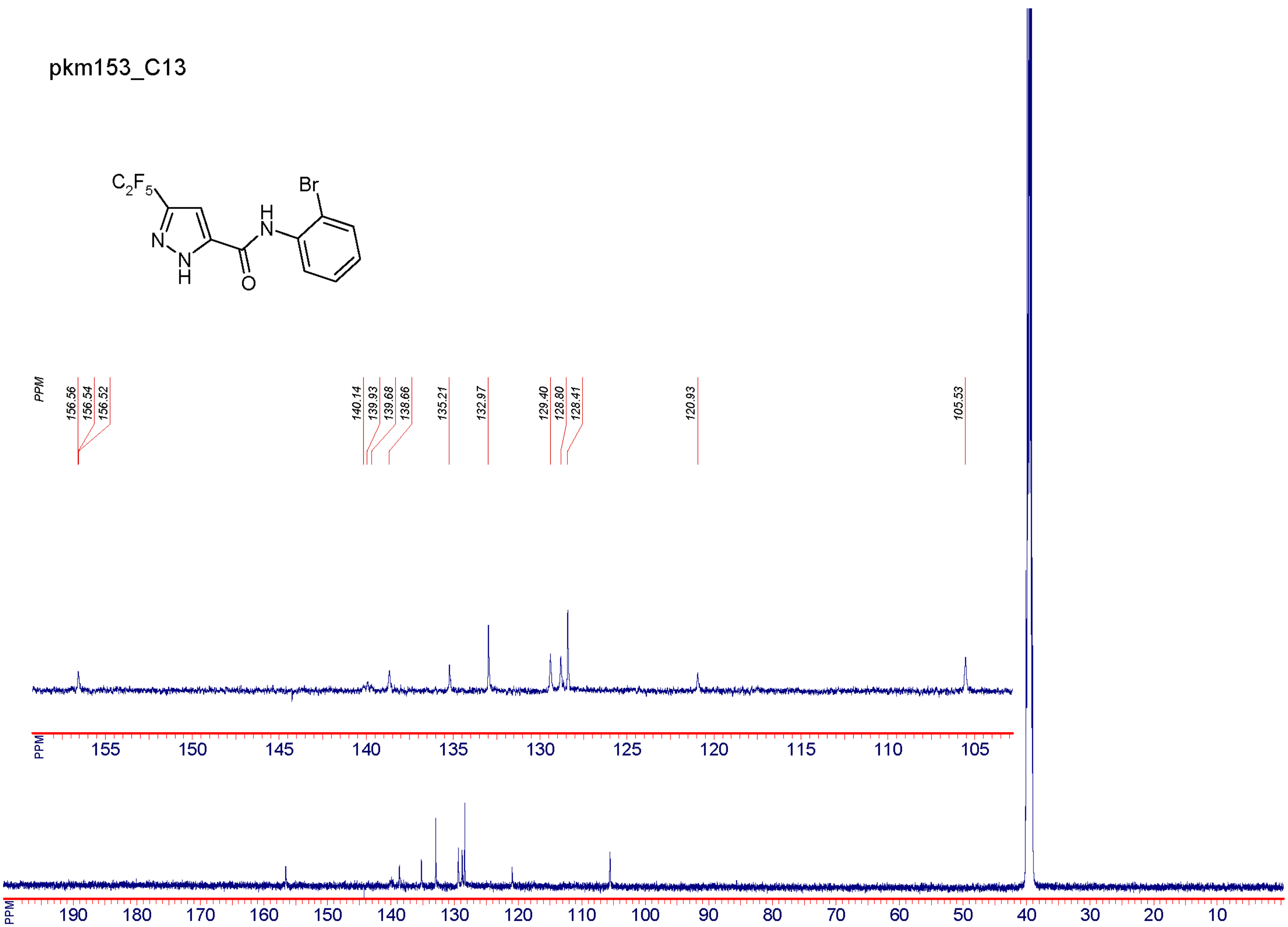

**File name: pkm153_C13**	Operator: root	SF: 125.7126 MHz	NSC: 6144	PW: 0.00 usec, RG: 51200	SI: 65536
Date: 07-Feb-2014	Solvent: DMSO	SW: 32680 Hz	TE: 0 K	AQ: 1.57 sec, RD: 0.00 sec	Parameter file, XWIN-NMR Version 3.5

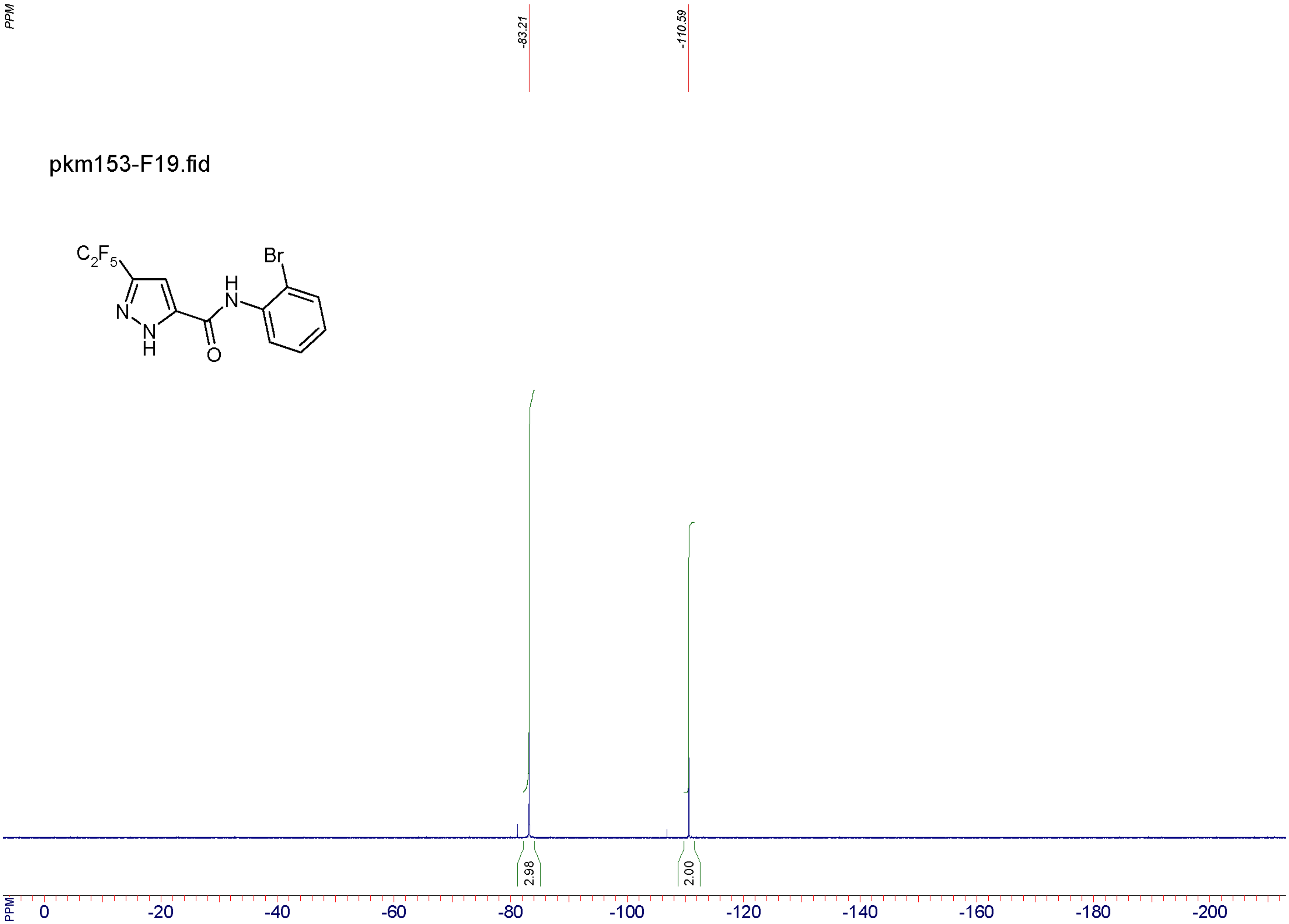

**File name: pkm153-F19.fid**	Operator:	SF: 376.3274 MHz	NSC: 0	PW: 3.00 usec, RG: 60	SI: 65536
Date: 06-Mar-2014	Solvent: dmso_d6	SW: 82816 Hz	TE: 293 K	AQ: 0.39 sec, RD: 0.00 sec	⊓

Compound 6a

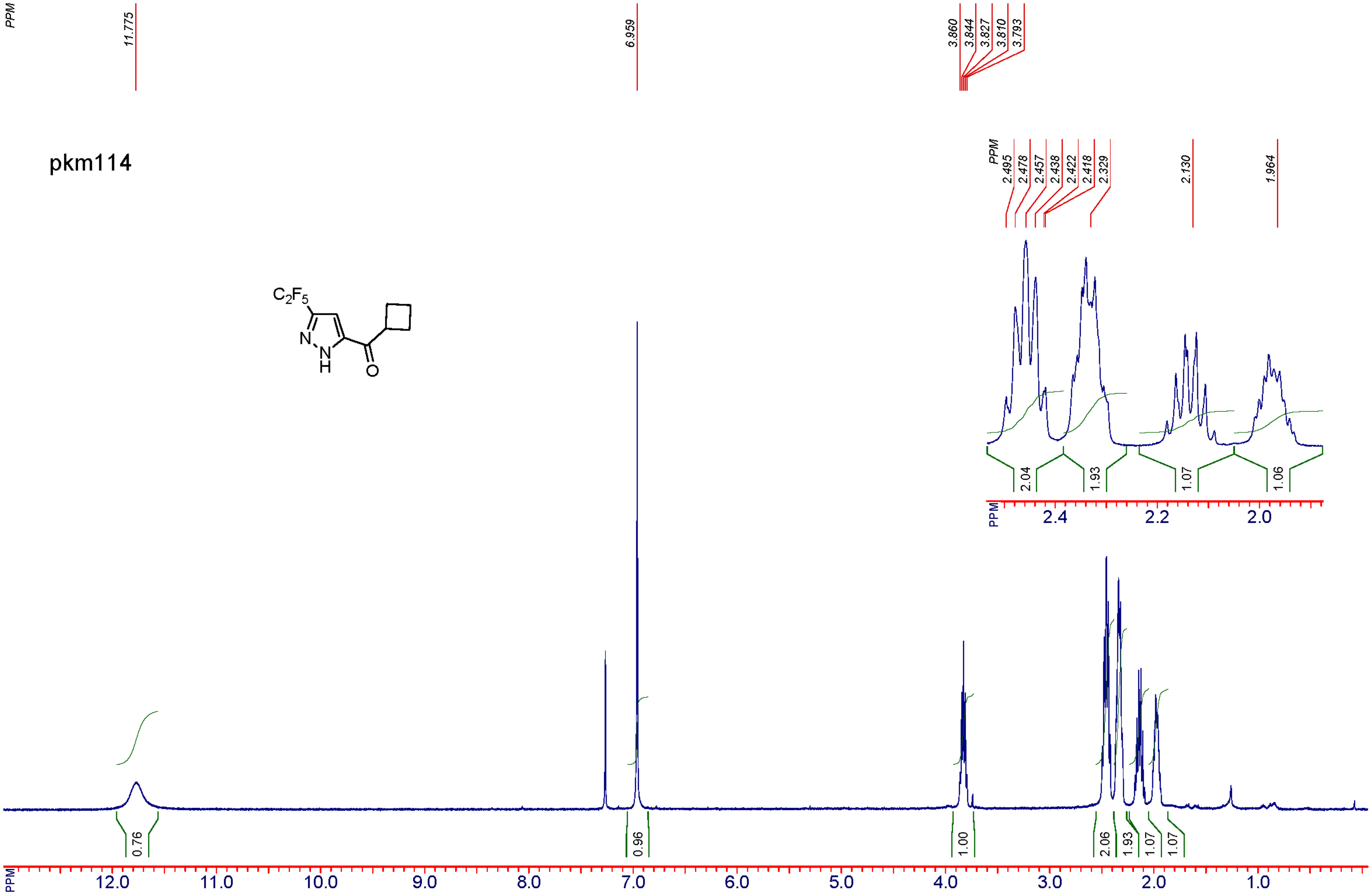

File name: pkm114	Operator: root	SF: 499.9500 MHz	NSC: 1	PW: 0.00 usec, RG: 100	SI: 32768
Date: 21-Nov-2013	Solvent: CDCl3	SW: 8993 Hz	TE: 0 K	AQ: 1.82 sec, RD: 0.00 sec	Parameter file, XWIN-NMRVersion 3.5

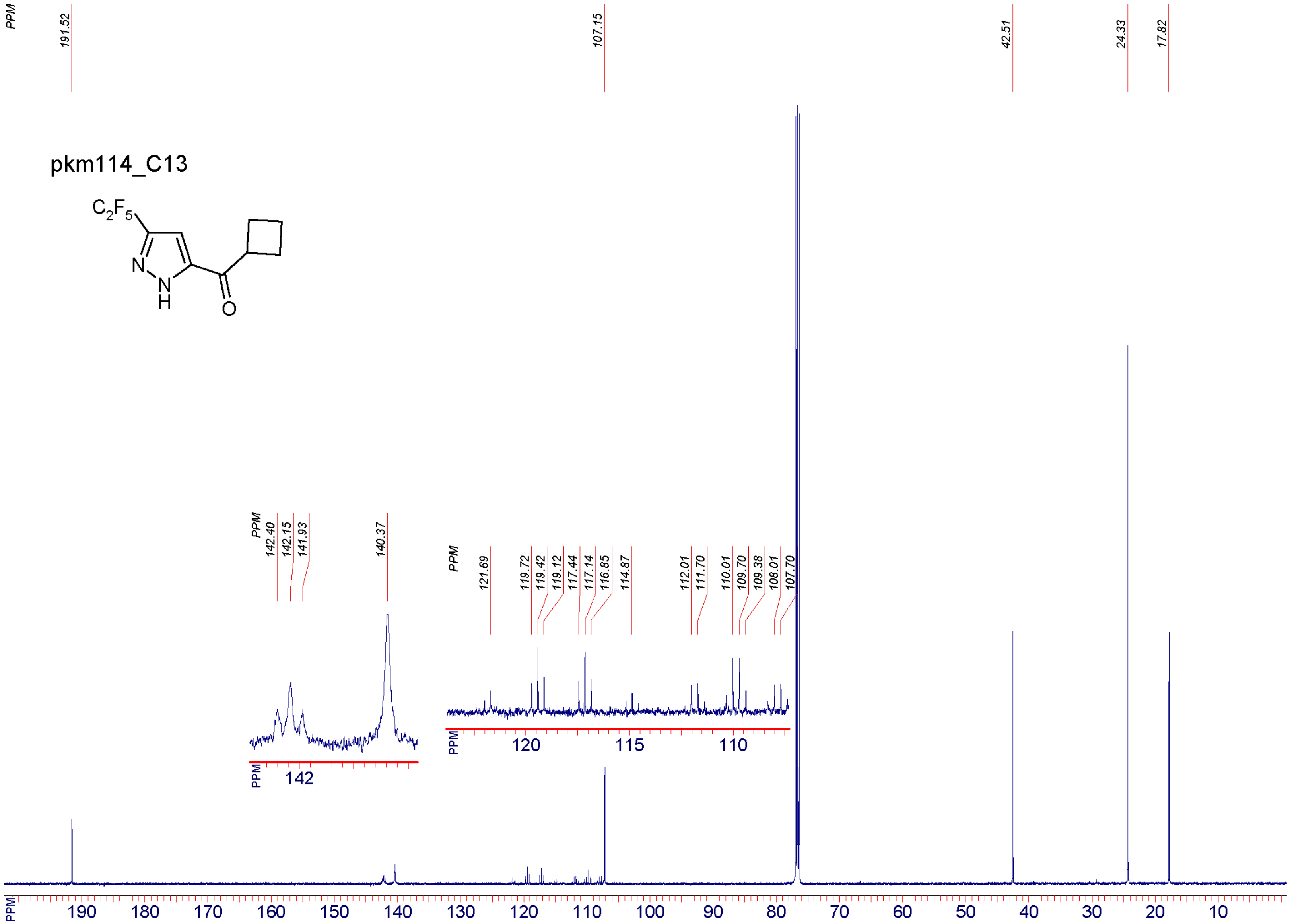

File name: pkm114_C13	Operator: root	SF: 125.7126 MHz	NSC: 10240	PW: 0.00 usec, RG: 51200	SI: 65536
Date: 07-Dec-2013	Solvent: dmso	SW: 32680 Hz	TE: 0 K	AQ: 1.57 sec, RD: 0.00 sec	Parameter file, XWIN-NMR Version 3.5

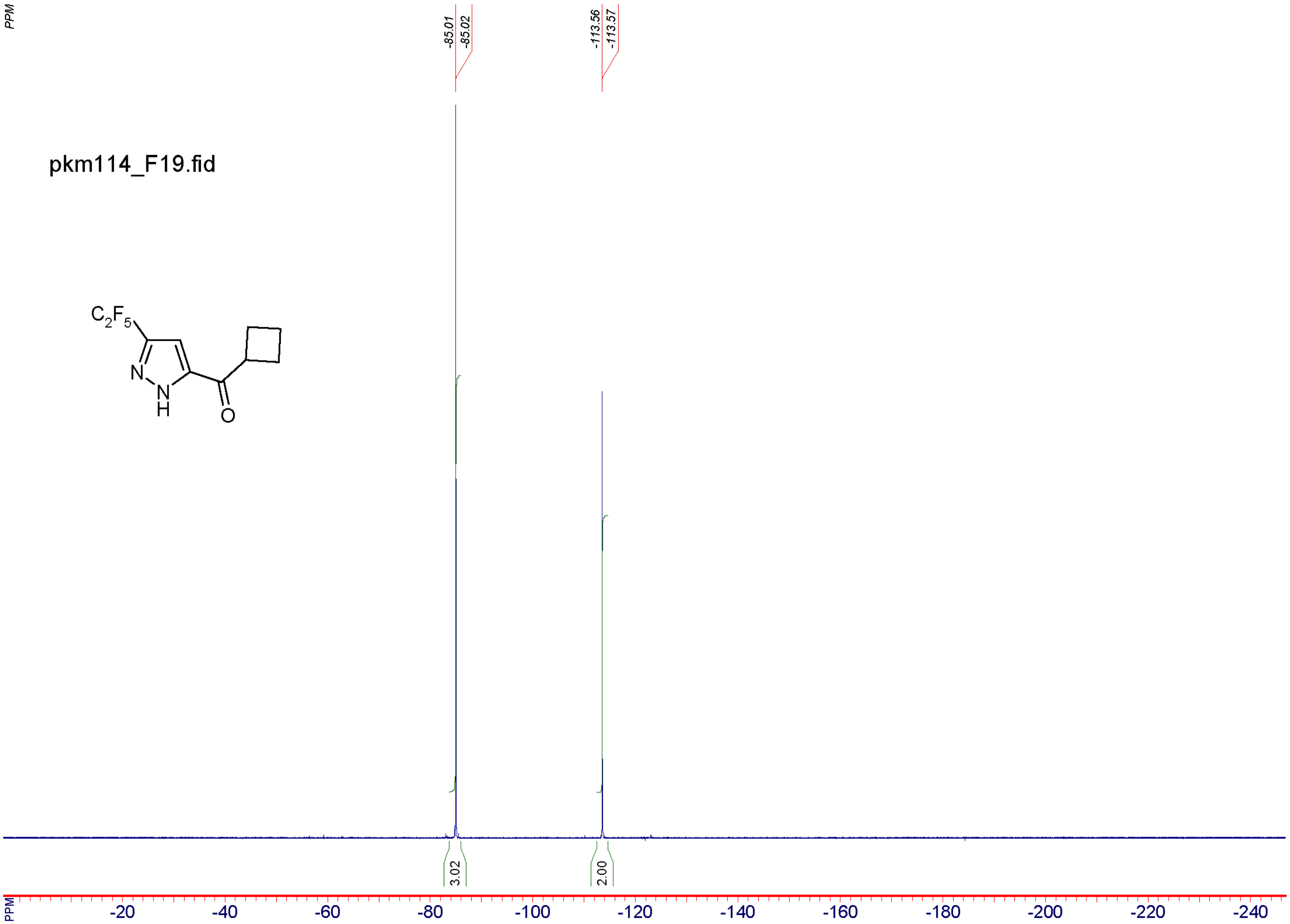

**File name: pkm114_F19.fid**	Operator:	SF: 376.3189 MHz	NSC: 0	PW: 3.00 usec, RG: 16	SI: 131072
Date: 10-Dec-2013	Solvent: cdcl3	SW: 94118 Hz	TE: 293 K	AQ: 0.68 sec, RD: 0.00 sec	⊓

Compound 7a

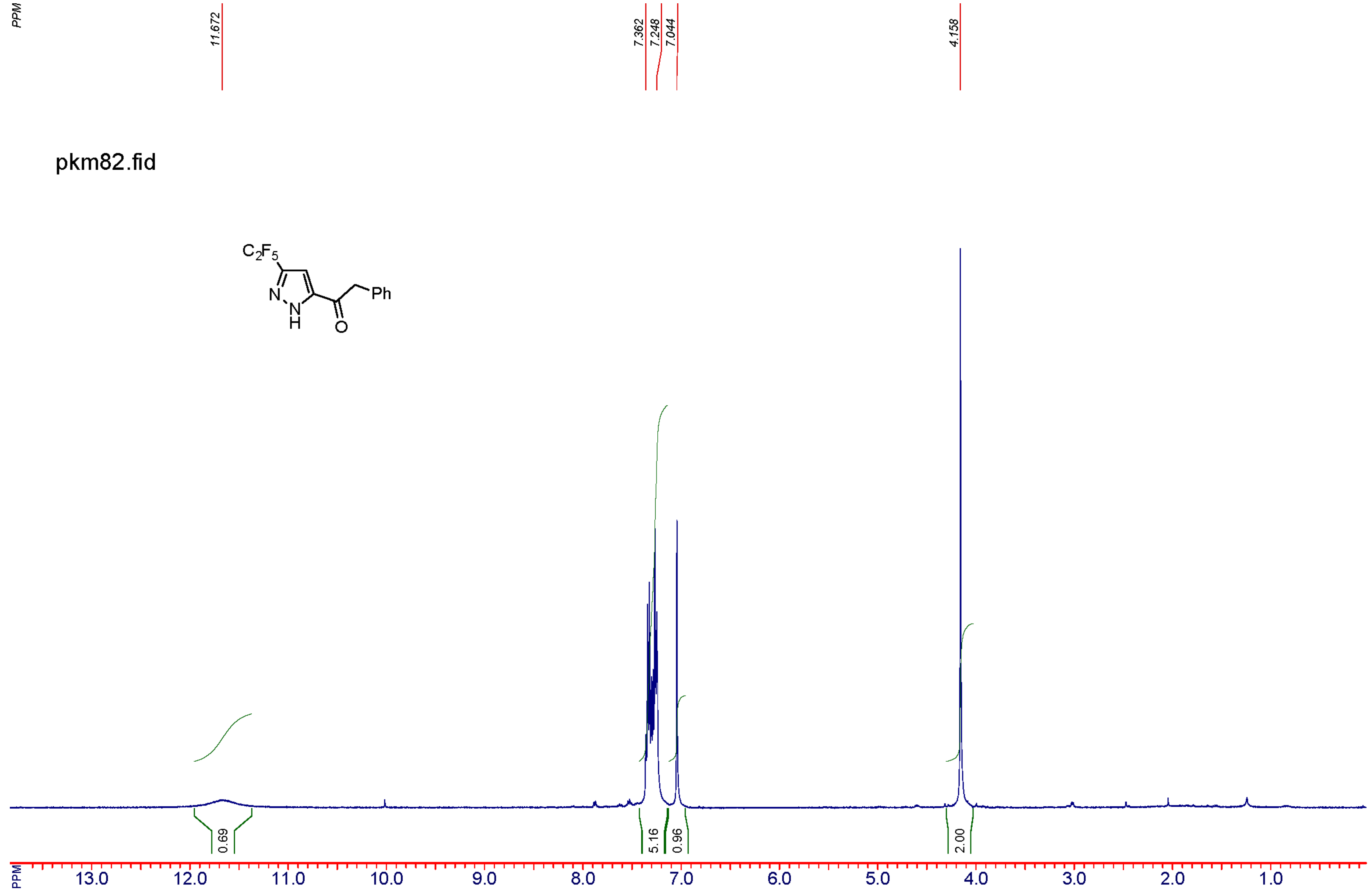

**File name: pkm82.fid**	Operator:	SF: 400.0002 MHz	NSC: 0	PW: 7.50 usec, RG: 26	SI: 32768
Date: 10-Oct-2013	Solvent: CDCl3	SW: 7261 Hz	TE: 293 K	AQ: 1.24 sec, RD: 0.00 sec	

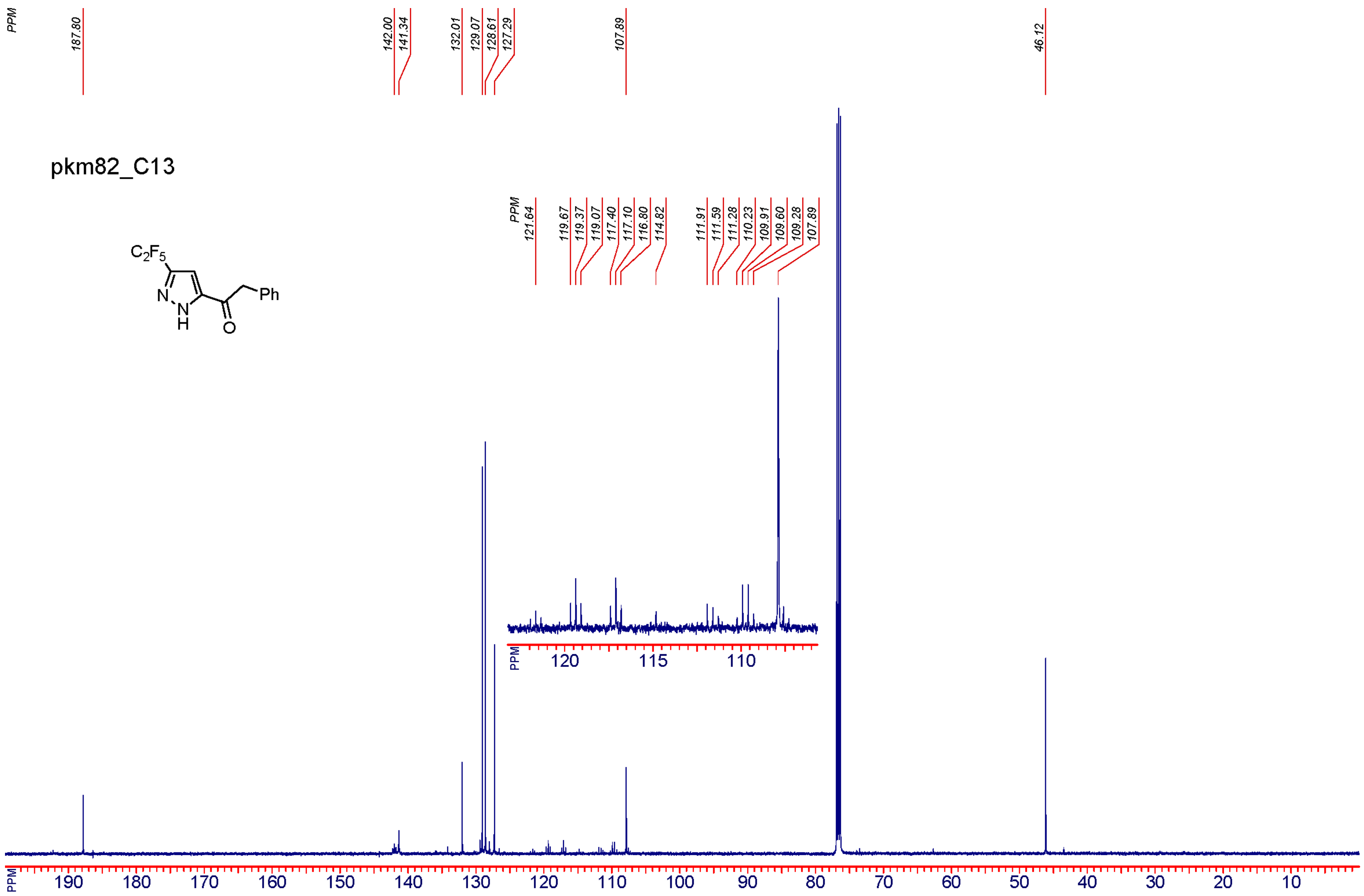

**File name: pkm82_C13**	Operator: root	SF: 125.7126 MHz	NSC: 6144	PW: 0.00 usec, RG: 51200	SI: 65536
Date: 13-Oct-2013	Solvent: CDCl3	SW: 32680 Hz	TE: 0 K	AQ: 1.57 sec, RD: 0.00 sec	Parameter file, XWIN-NMRVersion 3.5

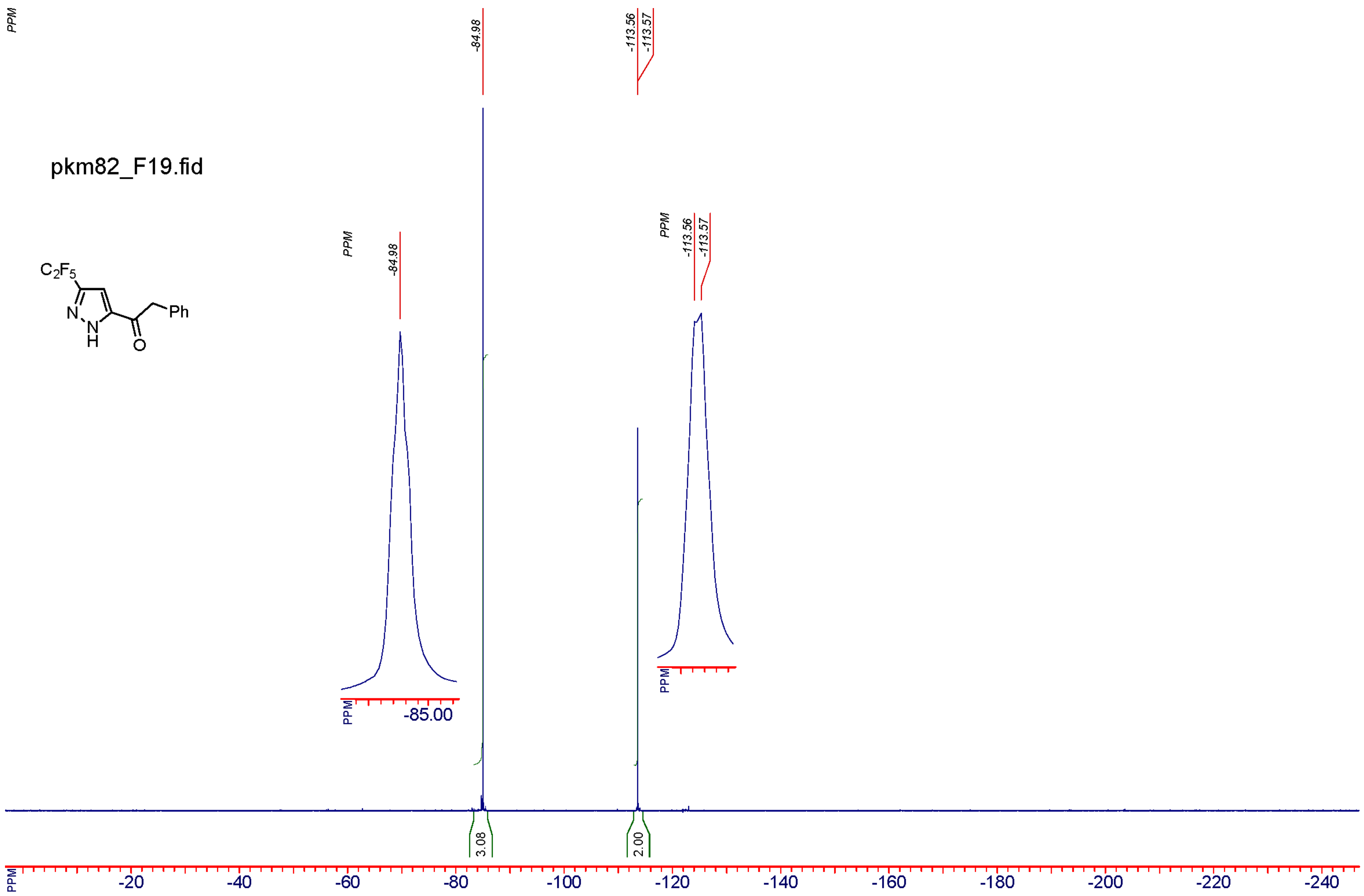

**File name: pkm82_F19.fid**	Operator:	SF: 376.3189 MHz	NSC: 0	PW: 3.00 usec, RG: 16	SI: 131072
Date: 15-Oct-2013	Solvent: cdcl3	SW: 94118 Hz	TE: 293 K	AQ: 0.68 sec, RD: 0.00 sec	

Compound 8a

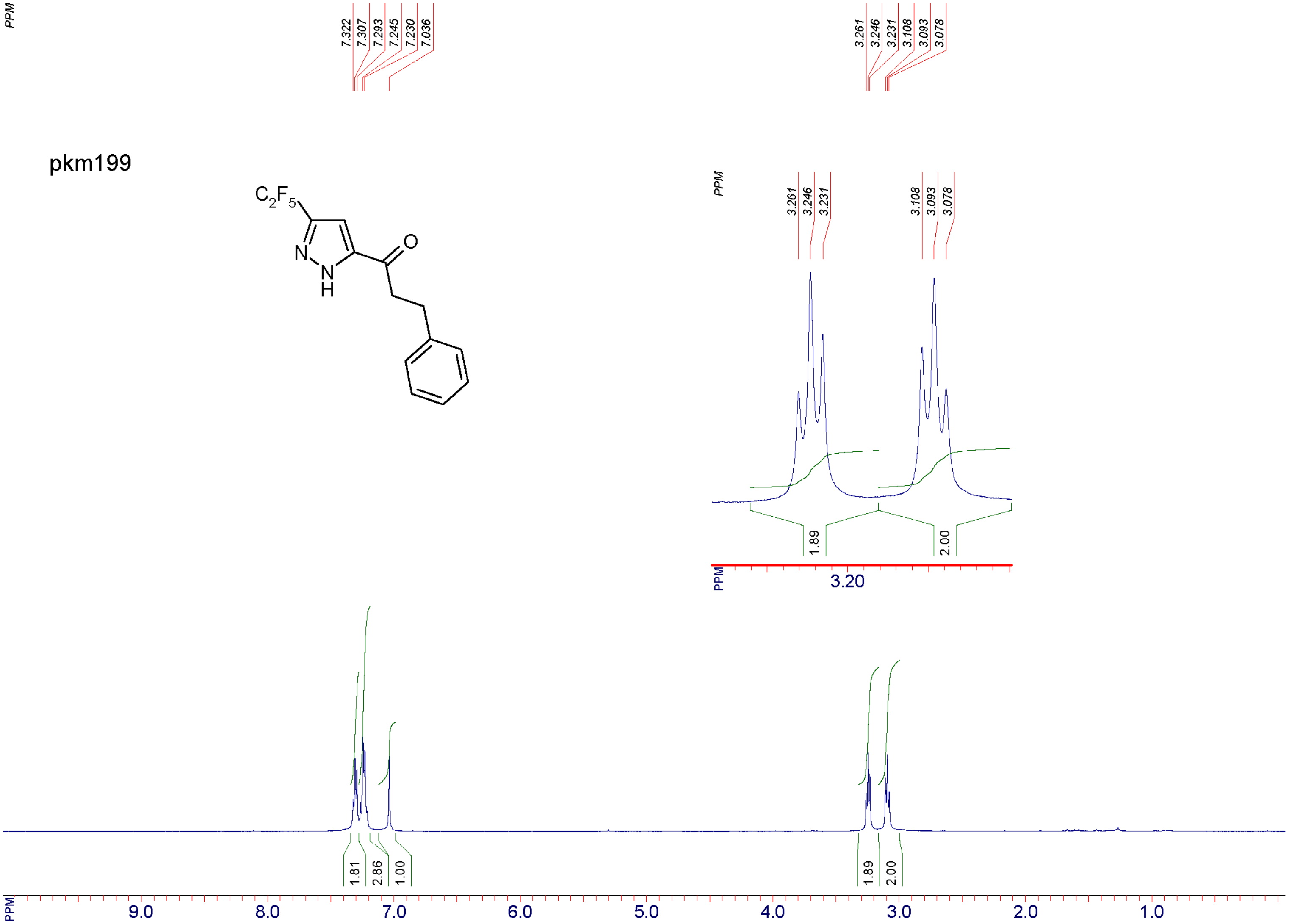

**File name: pkm199**	Operator: root	SF: 499.9500 MHz	NSC: 1	PW: 0.00 usec, RG: 40	SI: 32768
Date: 12-May-2014	Solvent: CDCl3	SW: 8993 Hz	TE: 0 K	AQ: 1.82 sec, RD: 0.00 sec	Parameter file, XWIN-NMR Version 3.5

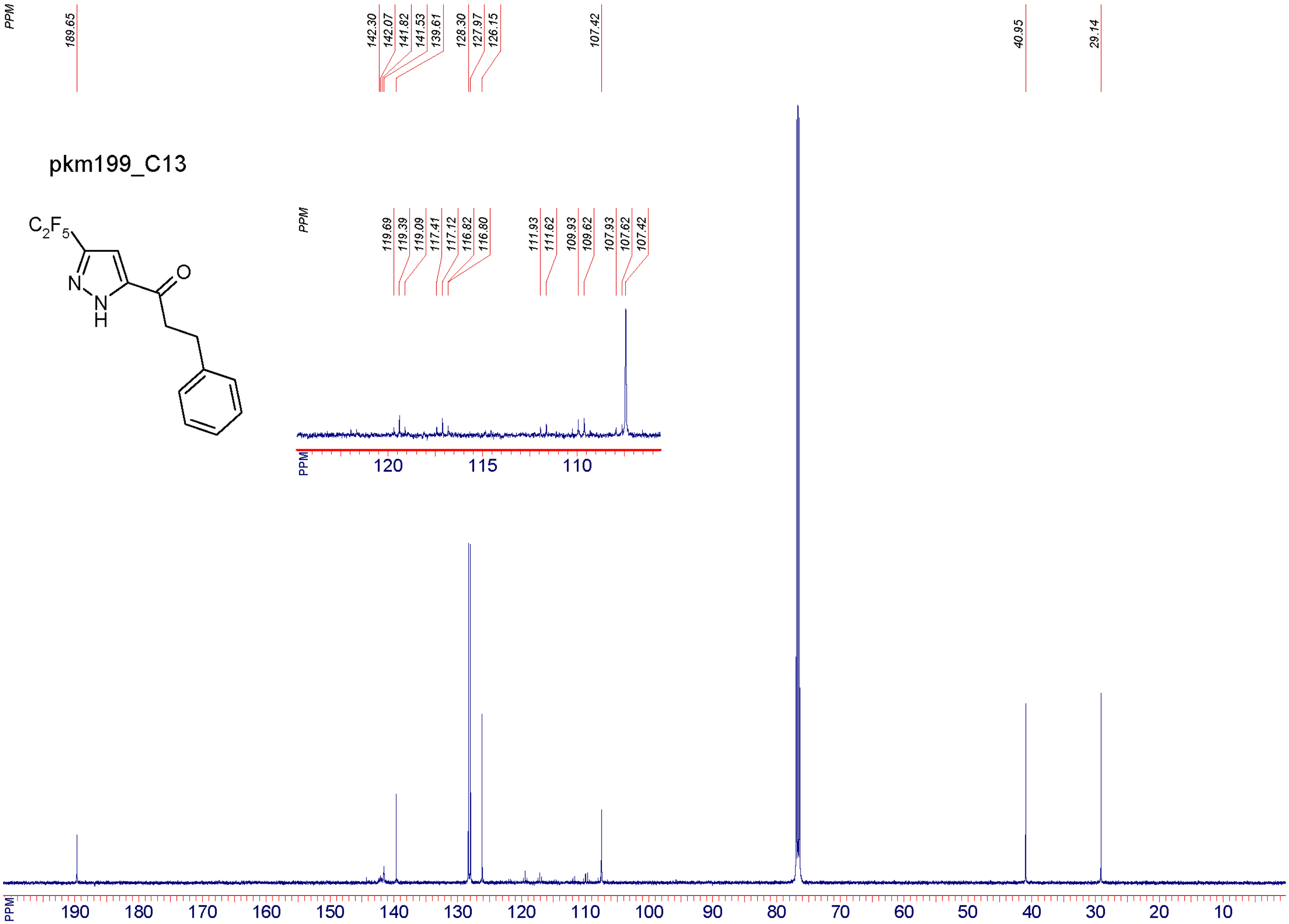

File name: pkm199_C13	Operator: root	SF: 125.7126 MHz	NSC: 5120	PW: 0.00 usec, RG: 51200	SI: 65536
Date: 14-May-2014	Solvent: CDCl3	SW: 32680 Hz	TE: 0 K	AQ: 1.57 sec, RD: 0.00 sec	Parameter file, XWIN-NMR Version 3.5

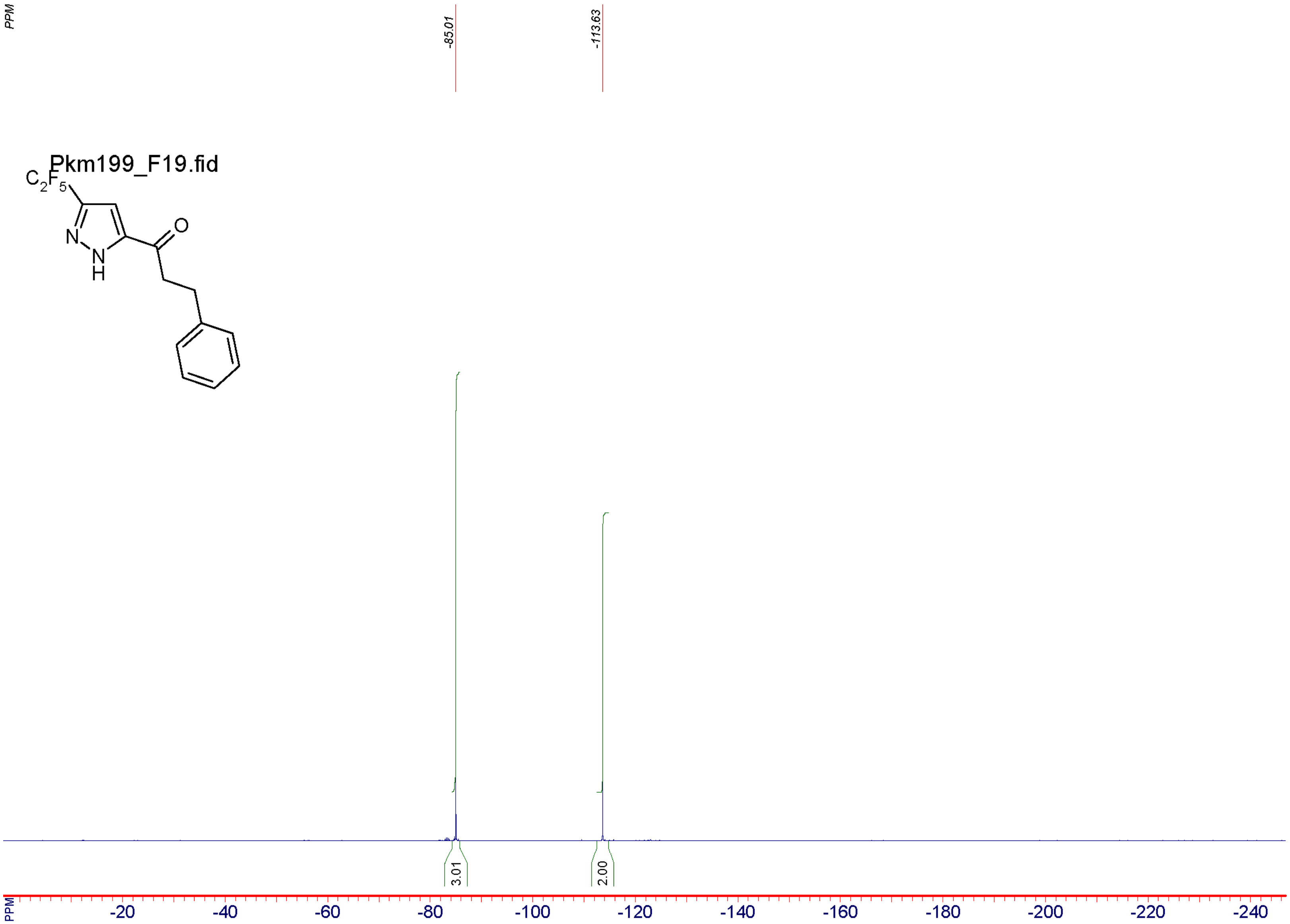

**File name: Pkm199_F19.fid**	Operator:	SF: 376.3189 MHz	NSC: 0	PW: 3.00 usec, RG: 32	SI: 65536
Date: 20-May-2014	Solvent: cdcl3	SW: 94118 Hz	TE: 293 K	AQ: 0.34 sec, RD: 0.00 sec	⊓

Compound 9a

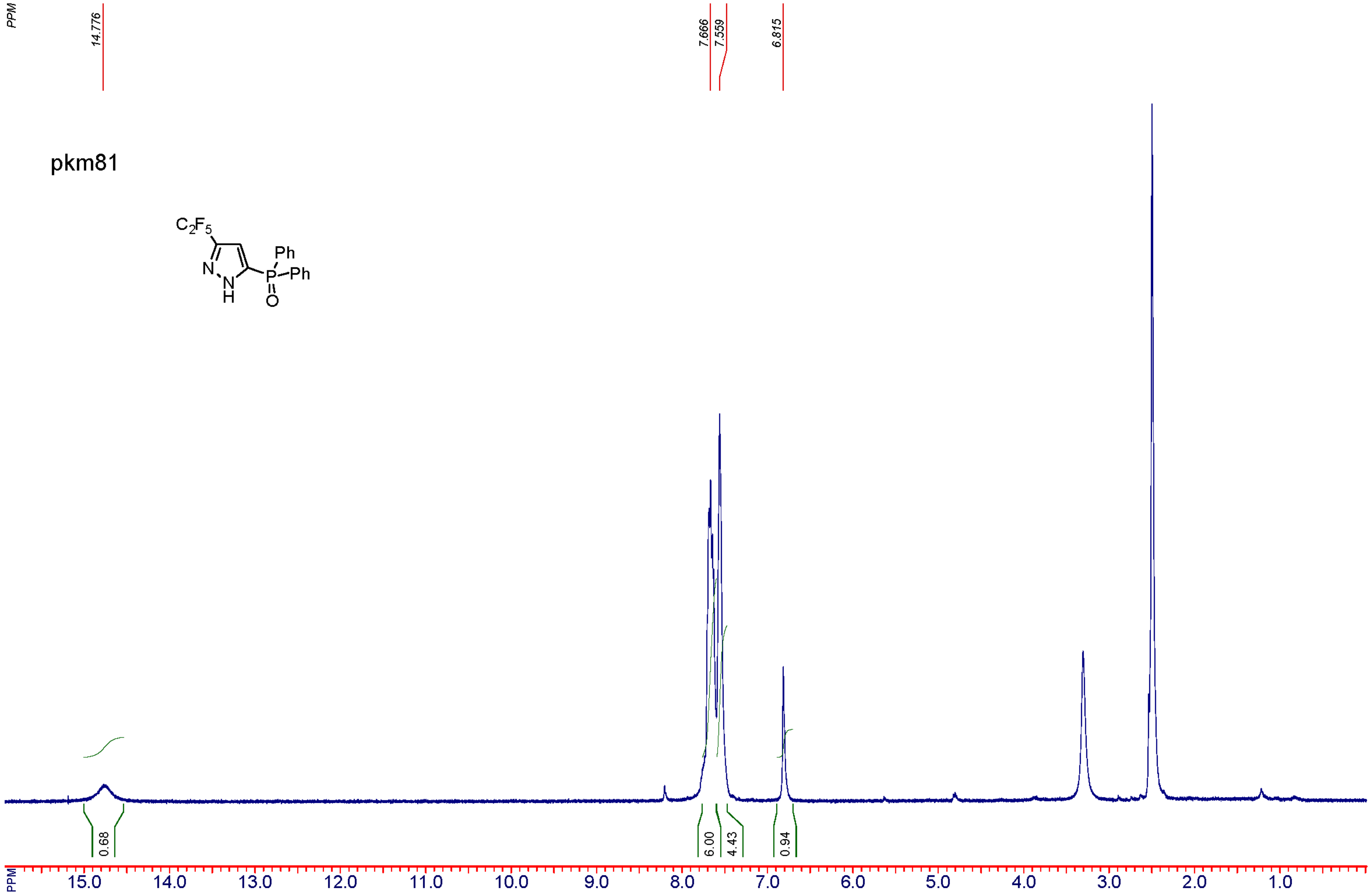

**File name: pkm81**	Operator: root	SF: 499.9500 MHz	NSC: 1	PW: 0.00 usec, RG: 32	SI: 32768
Date: 10-Oct-2013	Solvent: DMSO	SW: 8993 Hz	TE: 0 K	AQ: 1.82 sec, RD: 0.00 sec	Parameter file, XWIN-NMRVersion 3.5

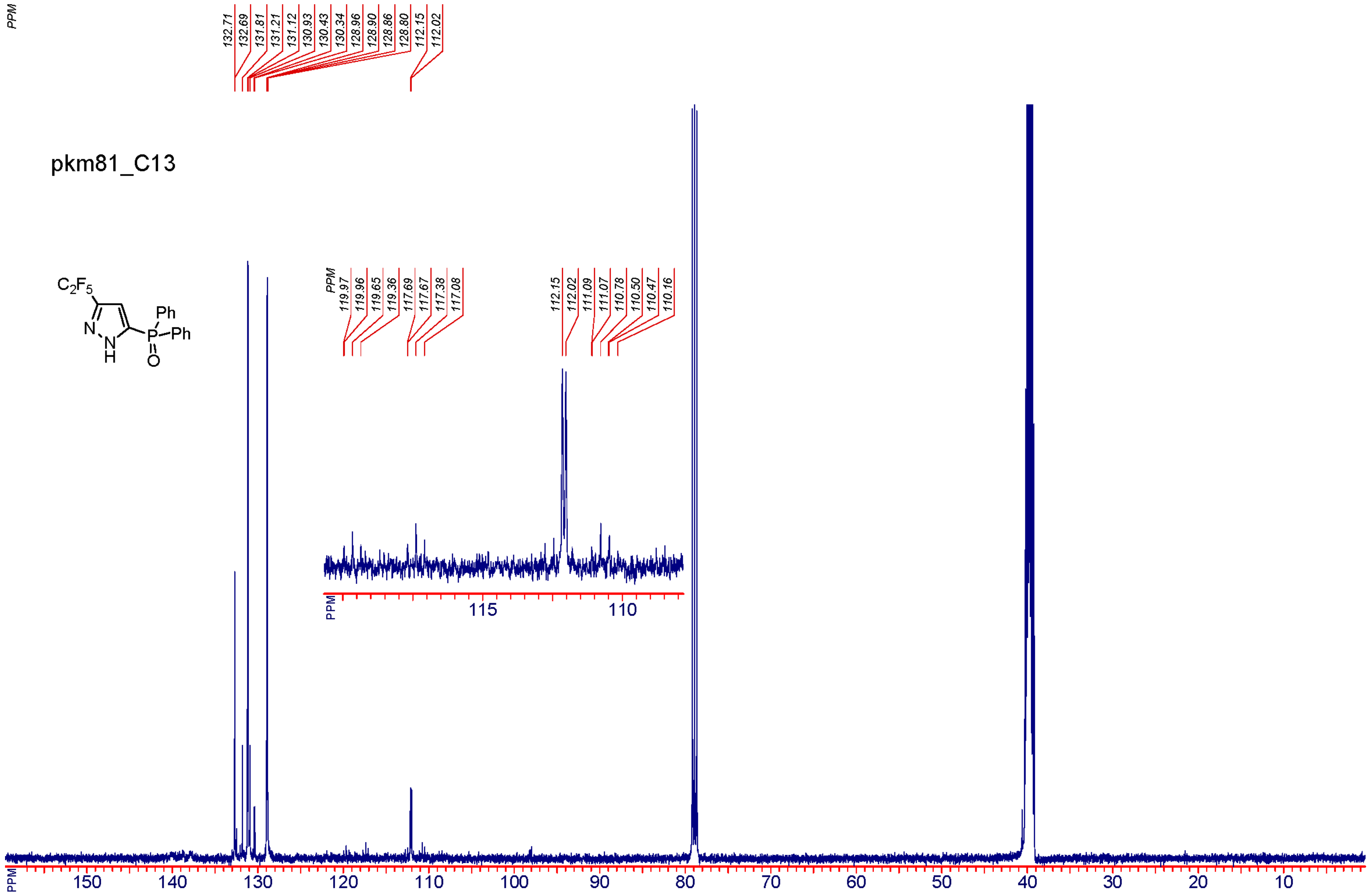

**File name: pkm81_C13**	Operator: root	SF: 125.7126 MHz	NSC: 10240	PW: 0.00 usec, RG: 51200	SI: 65536
Date: 12-Oct-2013	Solvent: DMSO	SW: 32680 Hz	TE: 0 K	AQ: 1.57 sec, RD: 0.00 sec	Parameter file, XWIN-NMRVersion 3.5

pkm81_F19.fid

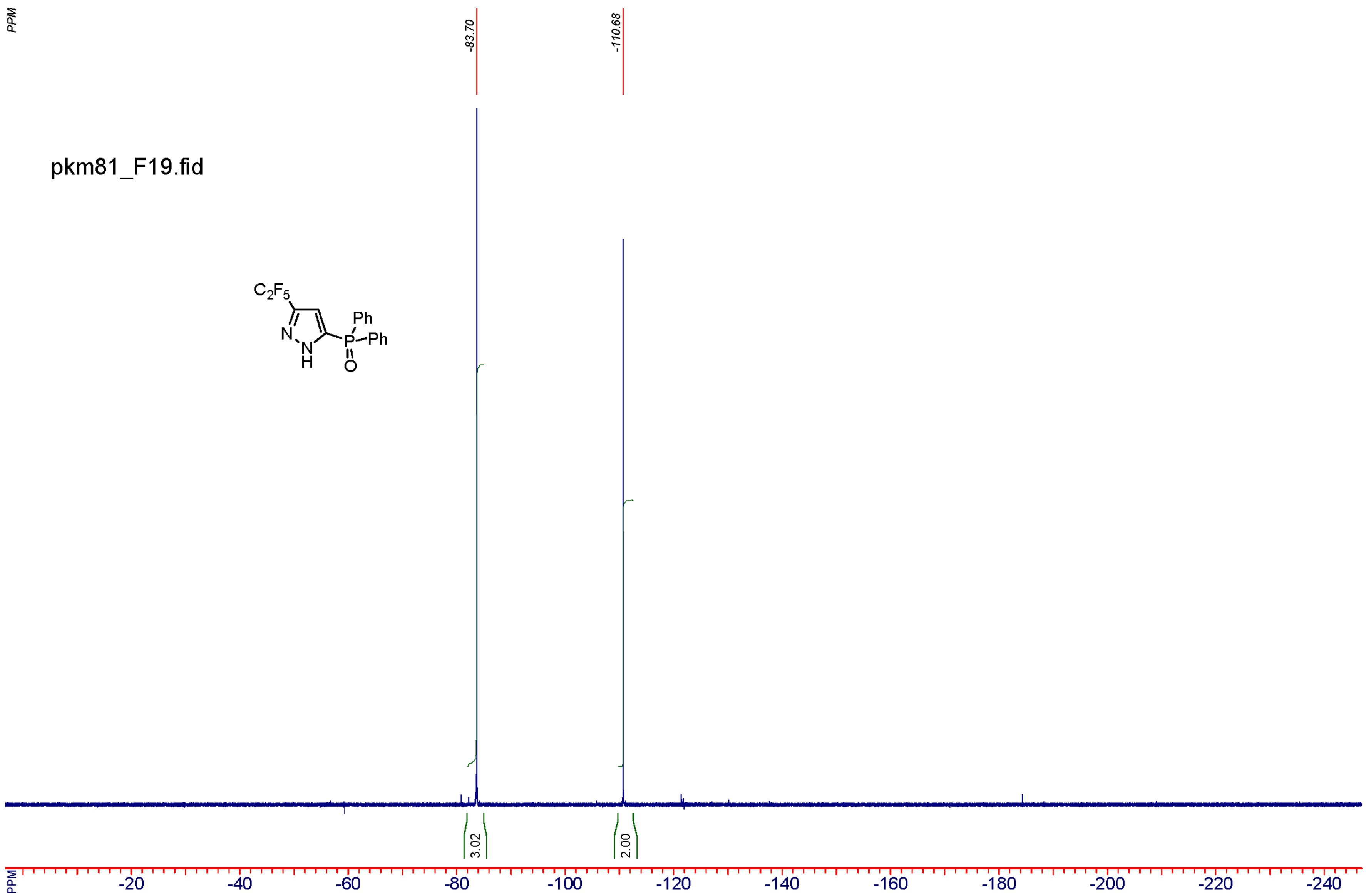

**File name: pkm81_F19.fid**	Operator:	SF: 376.3189 MHz	NSC: 0	PW: 3.00 usec, RG: 16	SI: 131072
Date: 22-Oct-2013	Solvent: cdcl3	SW: 94118 Hz	TE: 293 K	AQ: 0.68 sec, RD: 0.00 sec	

pkm81_P31

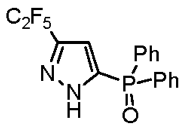

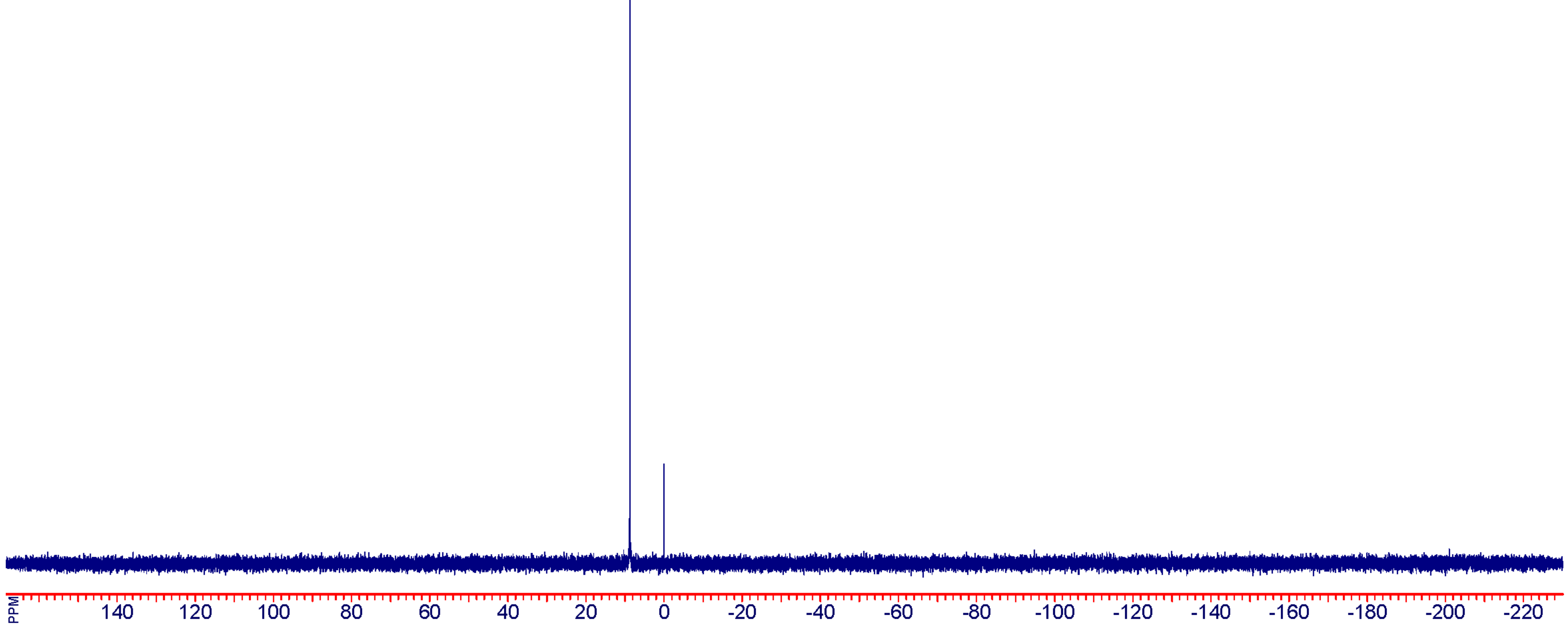

**File name: pkm81_P31**	Operator: root	SF: 202.3833 MHz	NSC: 12	PW: 0.00 usec, RG: 512	SI: 65536
Date: 24-Oct-2013	Solvent: CDCL3	SW: 80645 Hz	TE: 0 K	AQ: 0.81 sec, RD: 0.00 sec	Parameter file, XWIN-NMRVersion 3.5

Compound 10a

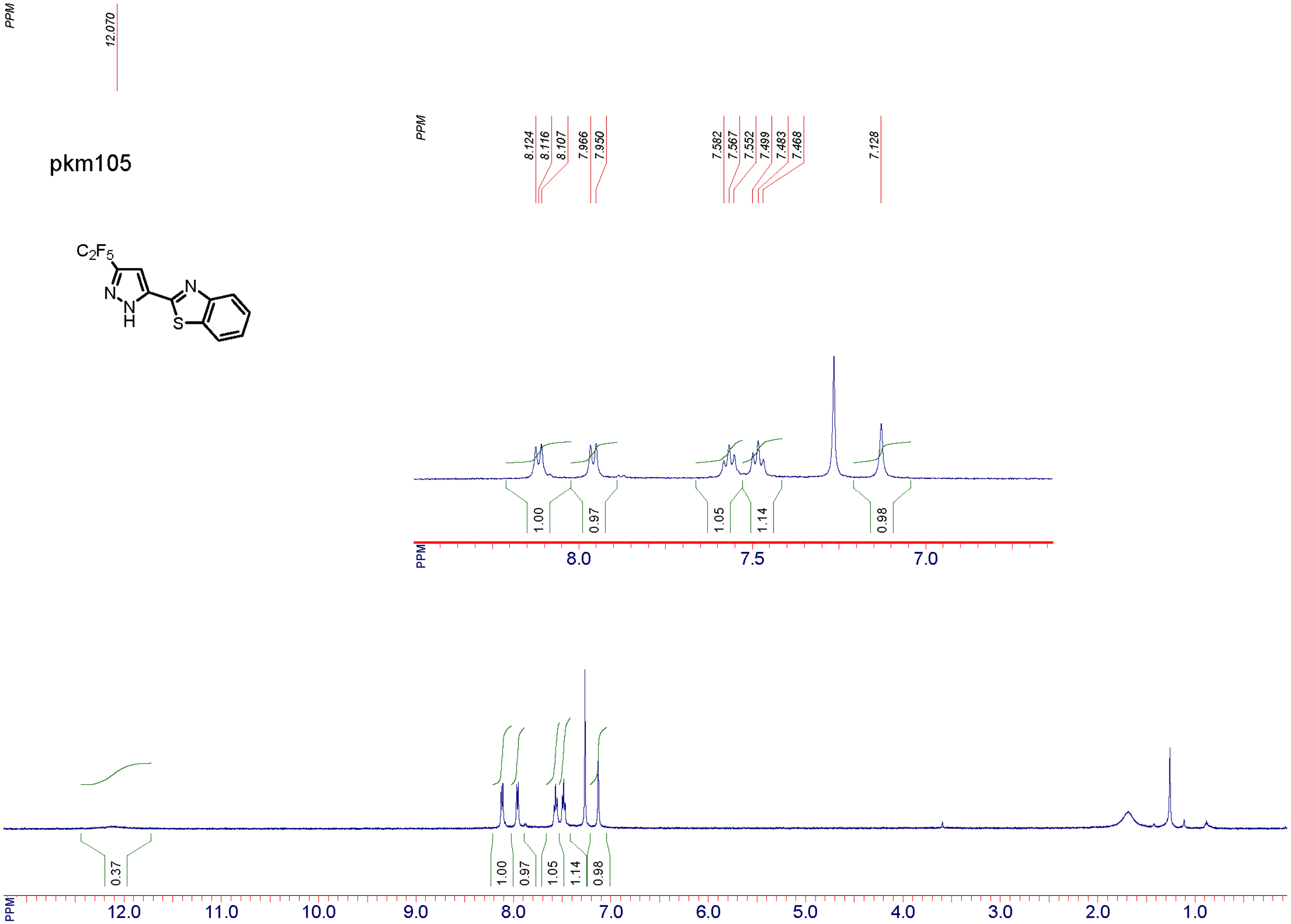

**File name: pkm105**	Operator: root	SF: 499.9500 MHz	NSC: 1	PW: 0.00 usec, RG: 24	SI: 32768
Date: 05-Nov-2013	Solvent: CDCl3	SW: 8993 Hz	TE: 0 K	AQ: 1.82 sec, RD: 0.00 sec	Parameter file, XWIN-NMR Version 3.5

pkm-105_C13

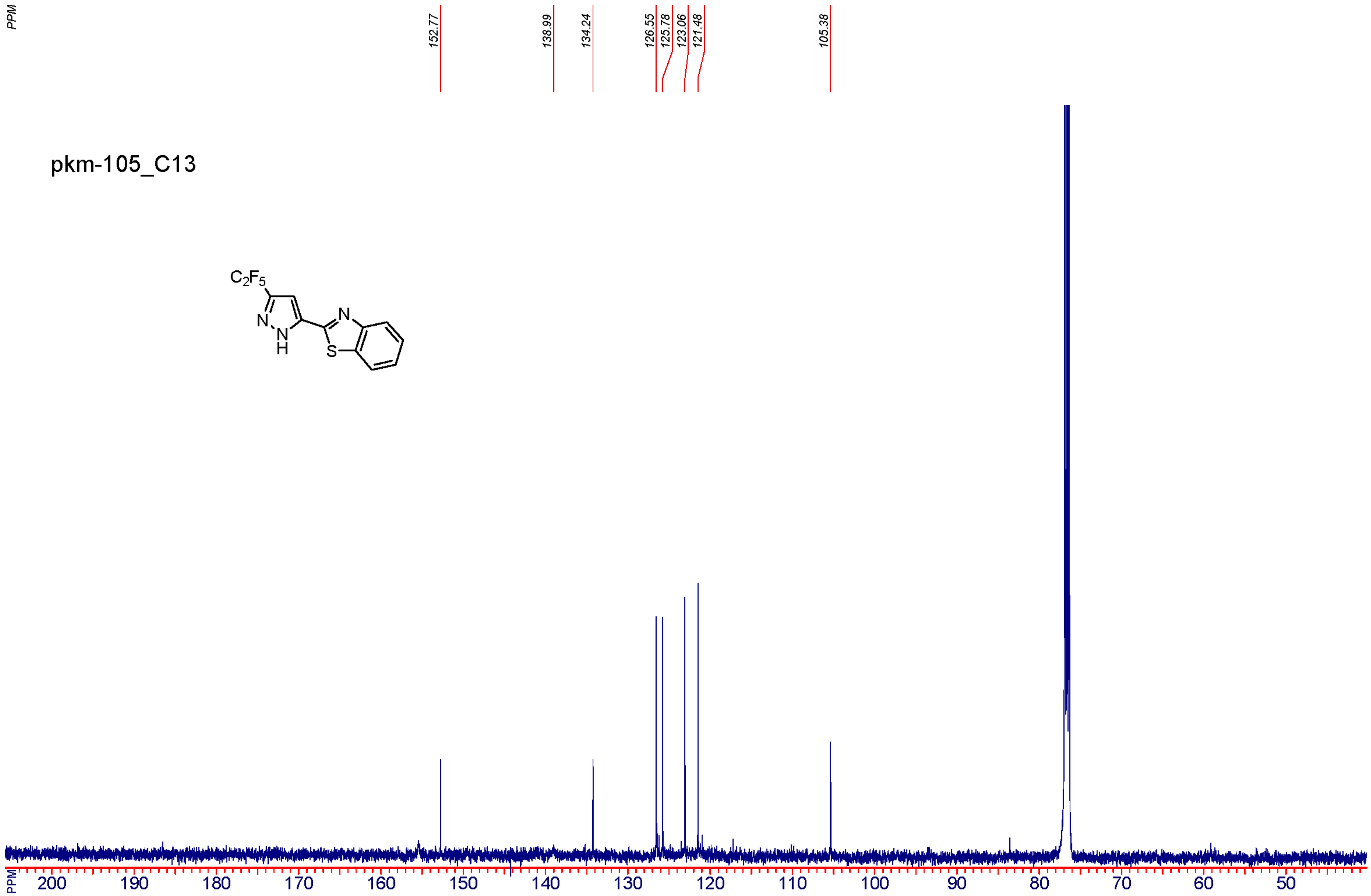

**File name: pkm-105_C13**	Operator: root	SF: 125.7126 MHz	NSC: 10240	PW: 0.00 usec, RG: 51200	SI: 65536
Date: 10-Nov-2013	Solvent: dmso	SW: 32680 Hz	TE: 0 K	AQ: 1.57 sec, RD: 0.00 sec	Parameter file, XWIN-NMRVersion 3.5

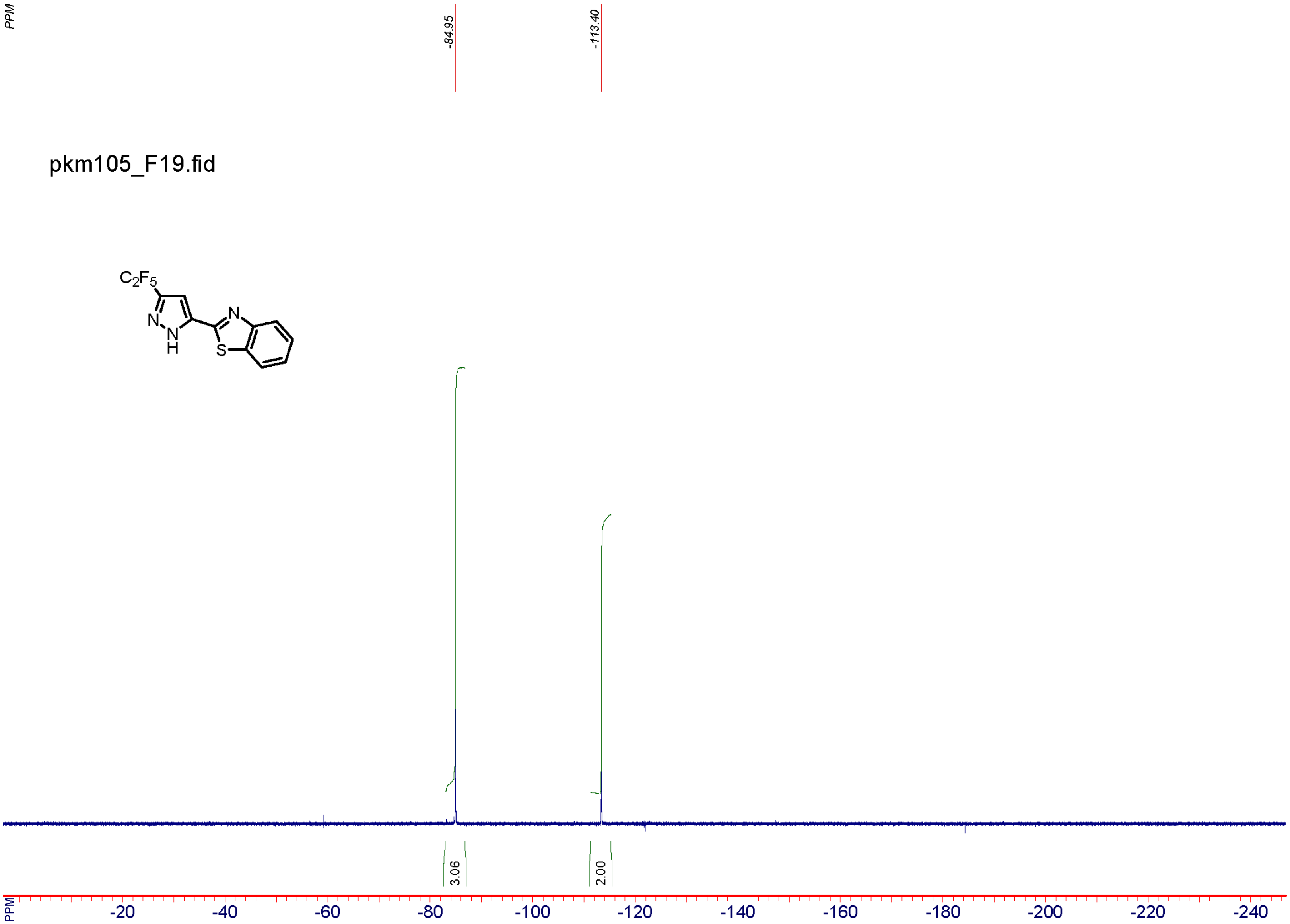

**File name: pkm105_F19.fid**	Operator:	SF: 376.3189 MHz	NSC: 0	PW: 3.00 usec, RG: 16	SI: 131072
Date: 12-Nov-2013	Solvent: cdcl3	SW: 94118 Hz	TE: 293 K	AQ: 0.68 sec, RD: 0.00 sec	□

Compound 11a

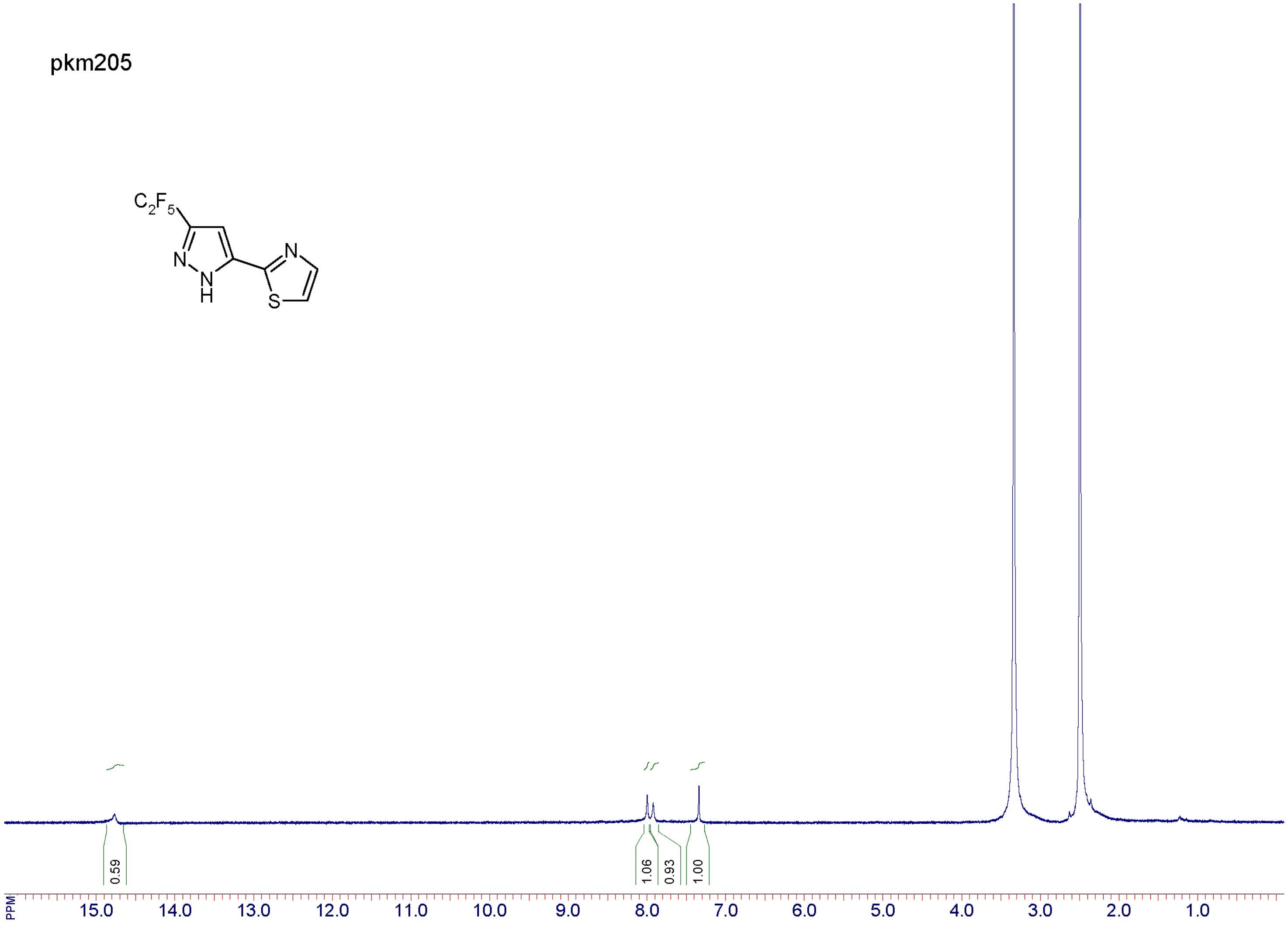

**File name: pkm205**	Operator: root	SF: 499.9500 MHz	NSC: 1	PW: 0.00 usec, RG: 250	SI: 32768
Date: 14-May-2014	Solvent: DMSO	SW: 8993 Hz	TE: 0 K	AQ: 1.82 sec, RD: 0.00 sec	Parameter file, XWIN-NMR Version 3.5

pkm205-2 C13

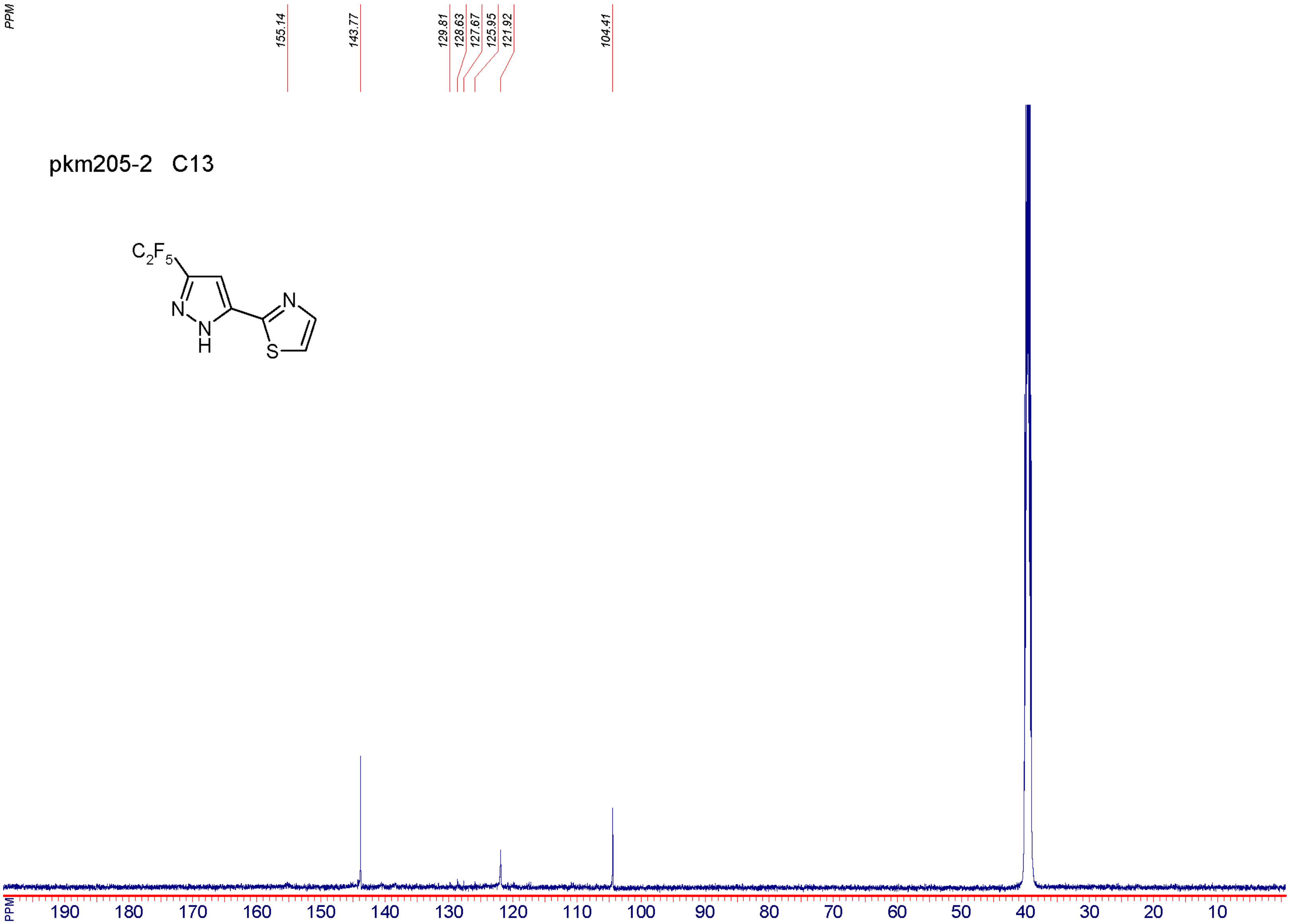

**File name: pkm205-2 C13**	Operator: root	SF: 125.7126 MHz	NSC: 10240	PW: 0.00 usec, RG: 51200	SI: 65536
Date: 17-May-2014	Solvent: DMSO	SW: 32680 Hz	TE: 0 K	AQ: 1.57 sec, RD: 0.00 sec	Parameter file, XWIN-NMR Version 3.5

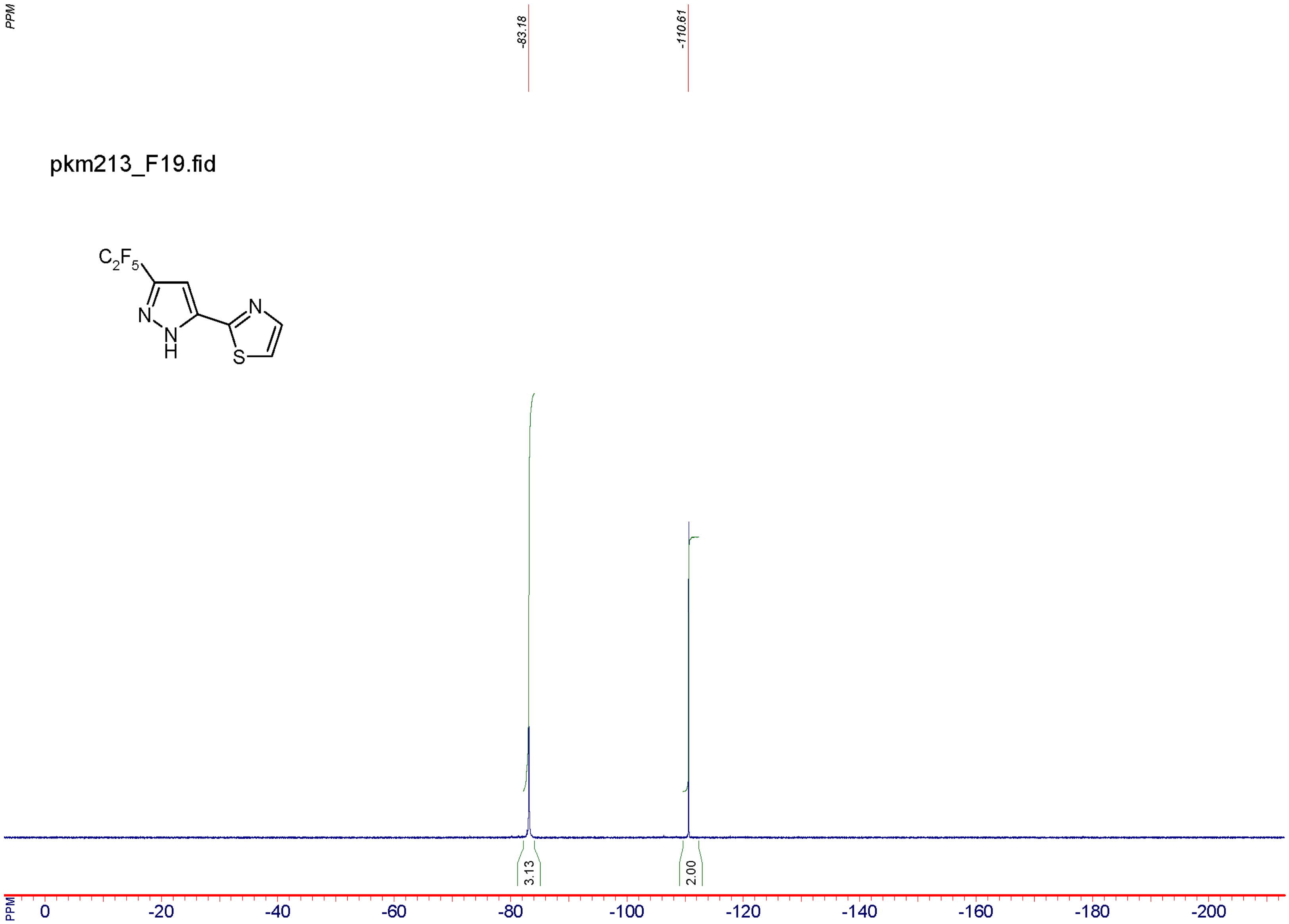

**File name: pkm213_F19.fid**	Operator:	SF: 376.3274 MHz	NSC: 0	PW: 3.00 usec, RG: 32	SI: 65536
Date: 22-May-2014	Solvent: dmso_d6	SW: 82816 Hz	TE: 293 K	AQ: 0.39 sec, RD: 0.00 sec	

Compound 12a

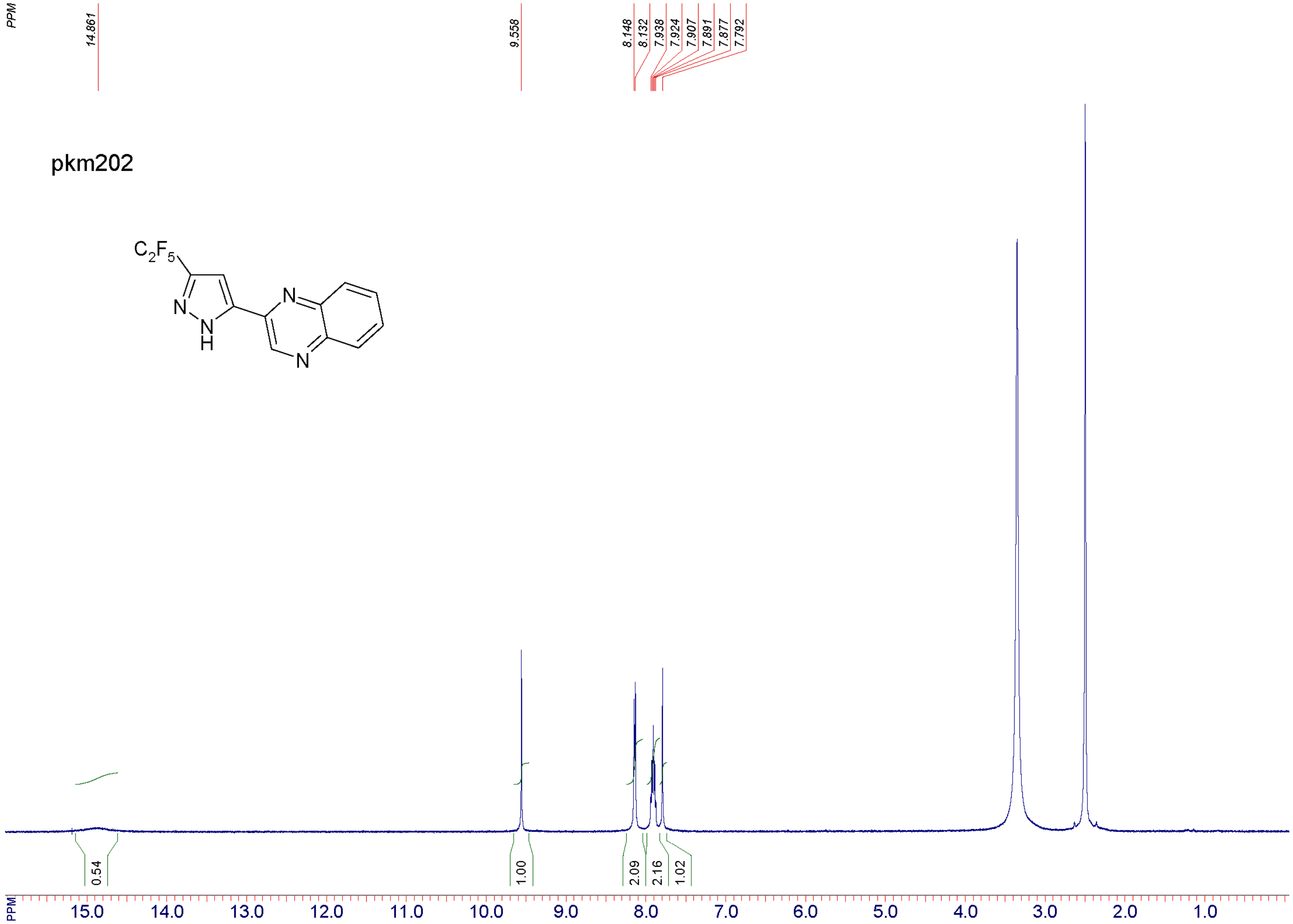

**File name: pkm202**	Operator: root	SF: 499.9500 MHz	NSC: 1	PW: 0.00 usec, RG: 40	SI: 32768
Date: 12-May-2014	Solvent: DMSO	SW: 8993 Hz	TE: 0 K	AQ: 1.82 sec, RD: 0.00 sec	Parameter file, XWIN-NMR Version 3.5

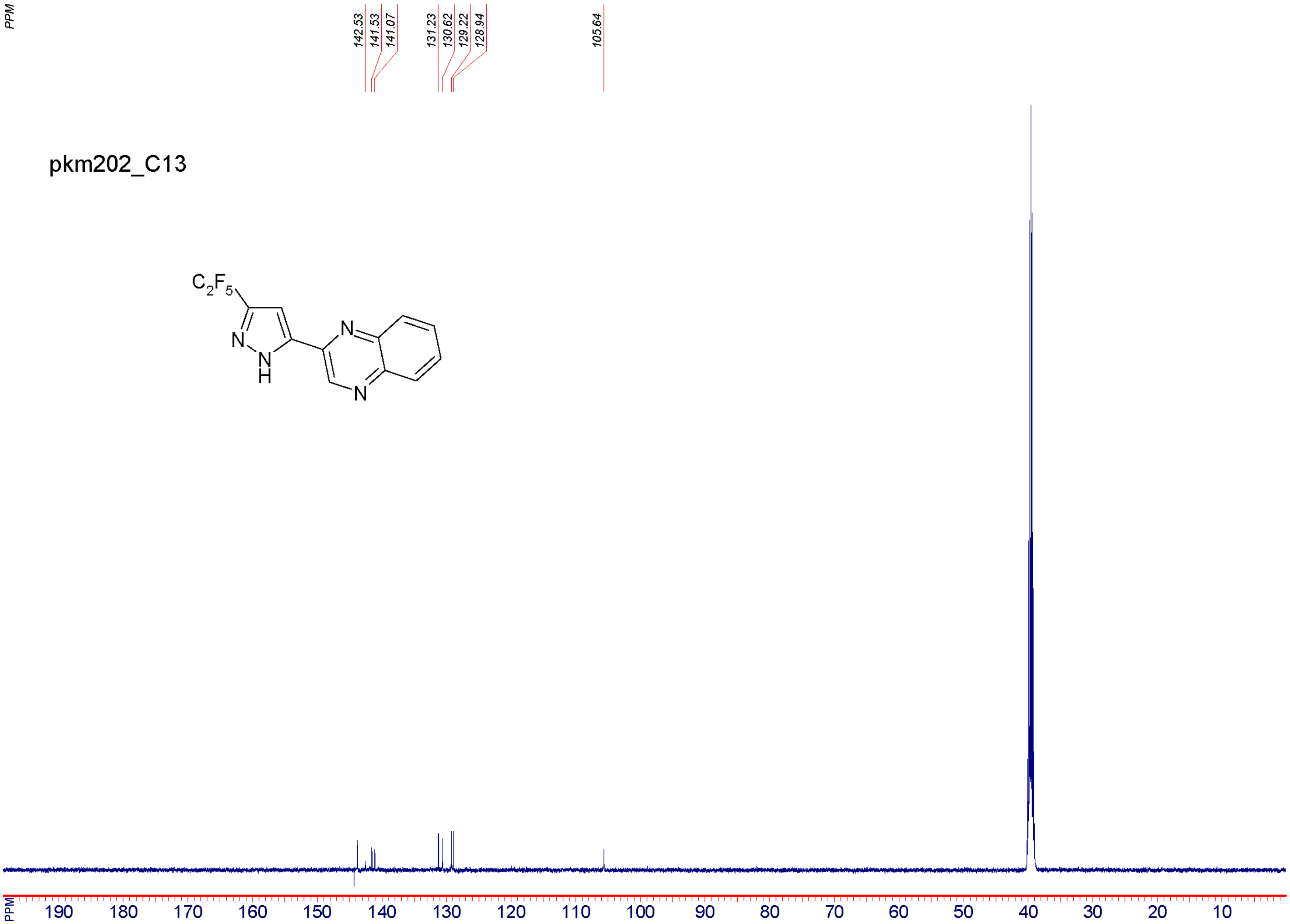

**File name: pkm202_C13**	Operator: root□	SF: 125.7126 MHz	NSC: 1364	PW: 0.00 usec, RG: 51200	SI: 65536
Date: 14-May-2014	Solvent: DMSO	SW: 32680 Hz	TE: 0 K	AQ: 1.57 sec, RD: 0.00 sec	Parameter file, XWIN-NMR□□Version 3.5□

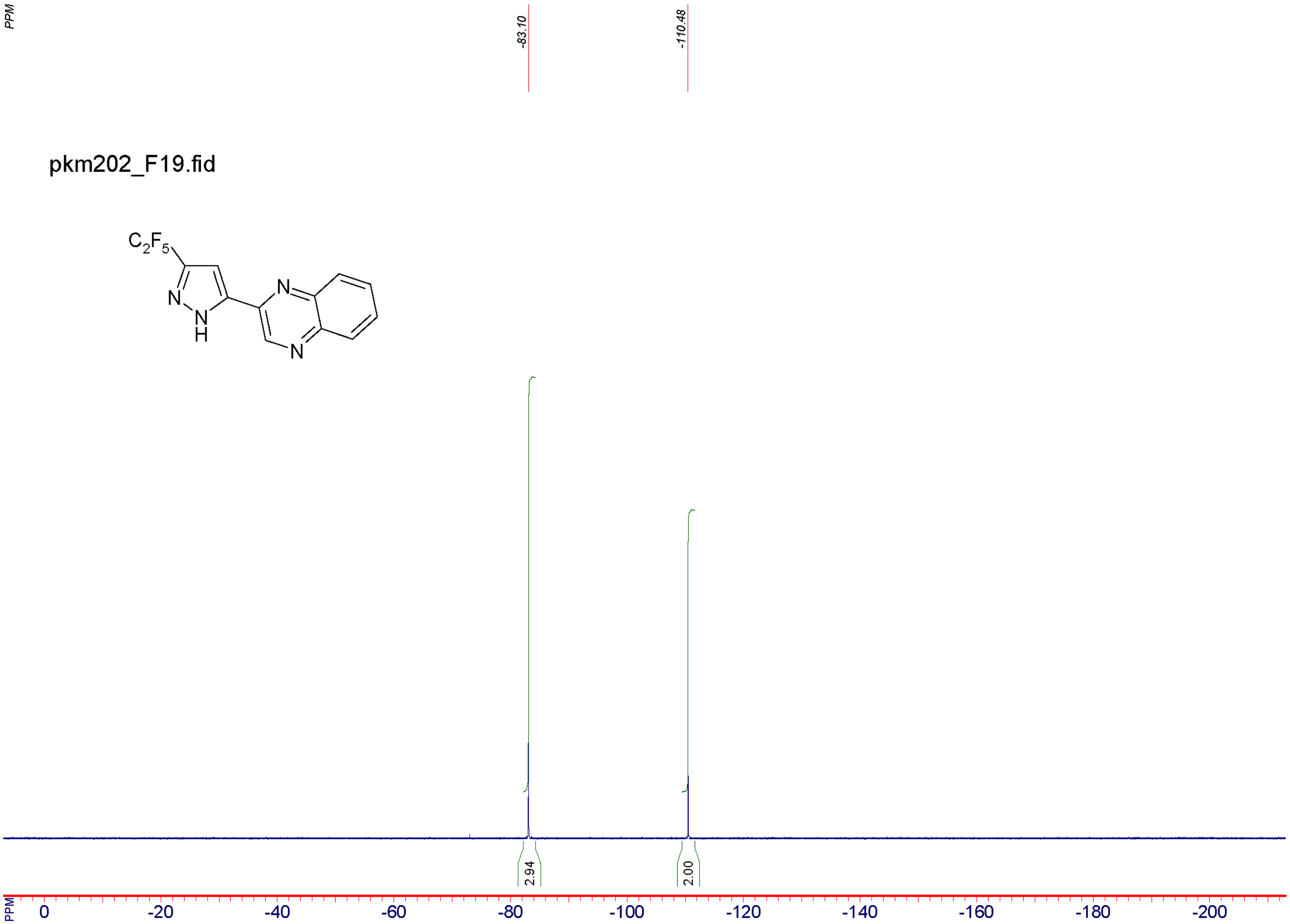

File name: pkm202_F19.fid	Operator:	SF: 376.3274 MHz	NSC: 0	PW: 3.00 usec, RG: 32	SI: 65536
Date: 20-May-2014	Solvent: dmso_d6	SW: 82816 Hz	TE: 293 K	AQ: 0.39 sec, RD: 0.00 sec	⊓

Compound 15a

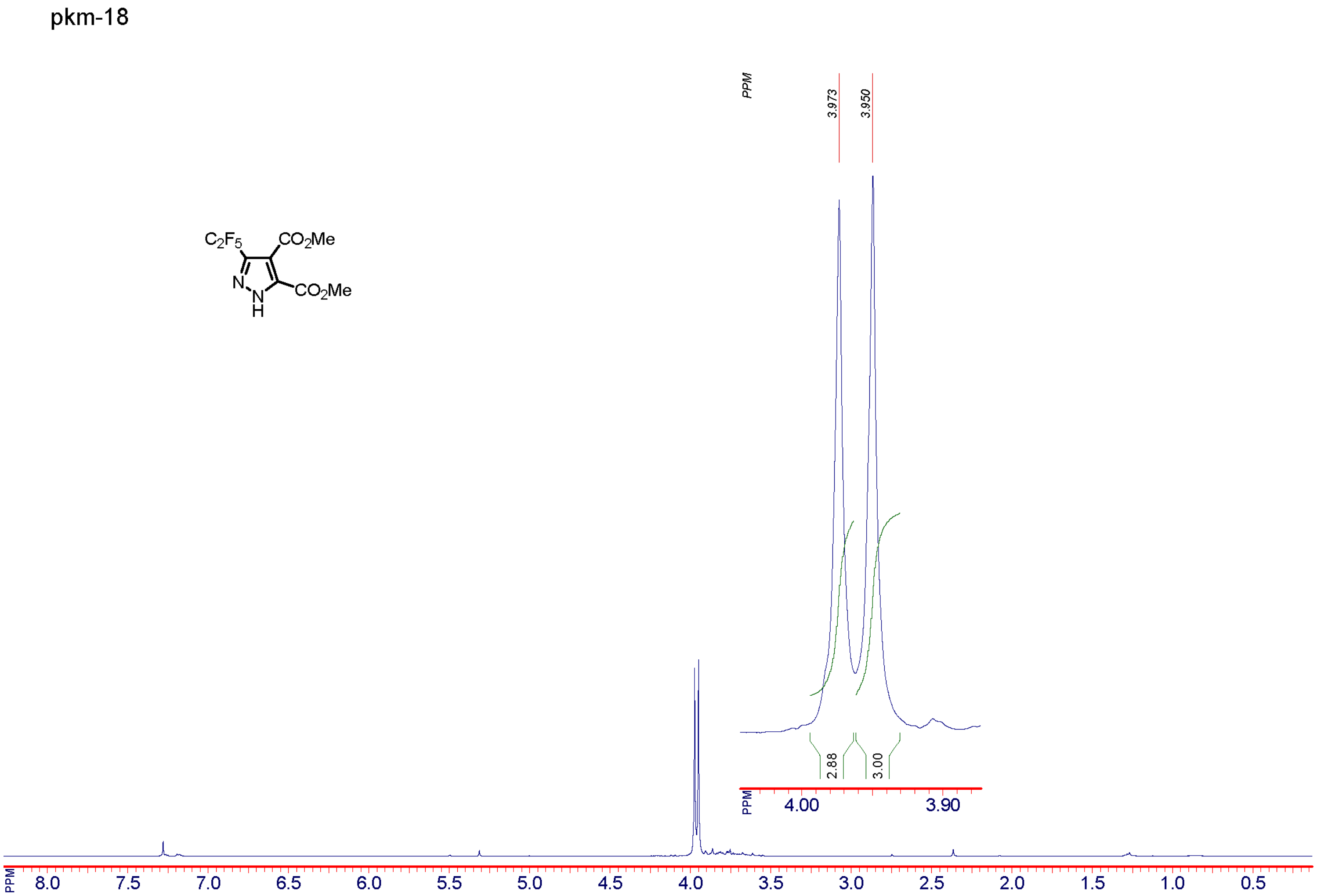

**File name: pkm-18**	Operator: root	SF: 500.0680 MHz	NSC: 1	PW: 0.00 usec, RG: 32	SI: 32768
Date: 14-Jun-2013	Solvent: CDCl3	SW: 8503 Hz	TE: 0 K	AQ: 1.93 sec, RD: 0.00 sec	Parameter file, XWIN-NMR Version 3.5

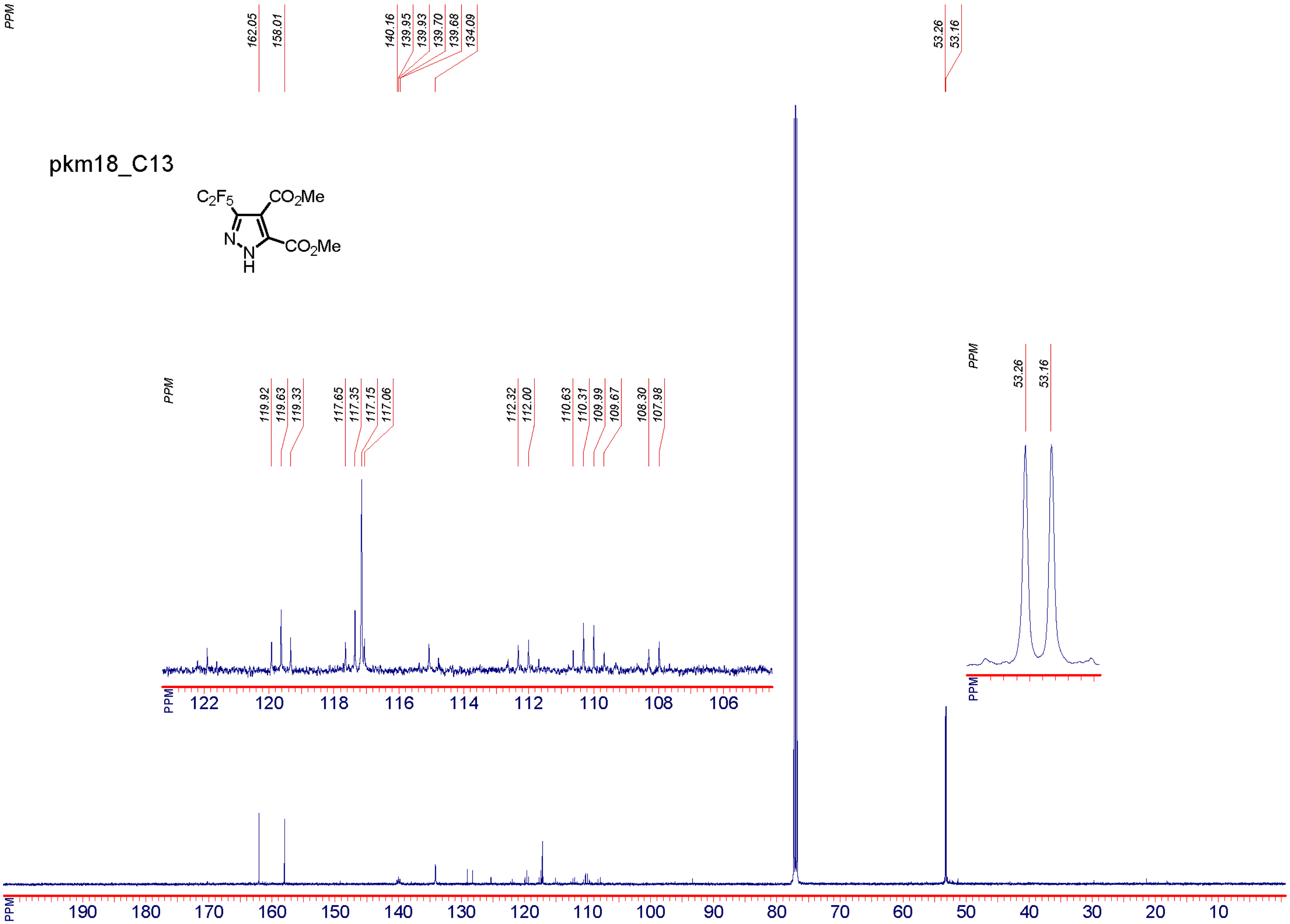

**File name: pkm18_C13**	Operator: root	SF: 125.7422 MHz	NSC: 10240	PW: 0.00 usec, RG: 51200	SI: 65536
Date: 16-Jun-2013	Solvent: CDCl3	SW: 32680 Hz	TE: 0 K	AQ: 1.57 sec, RD: 0.00 sec	Parameter file, XWIN-NMR Version 3.5

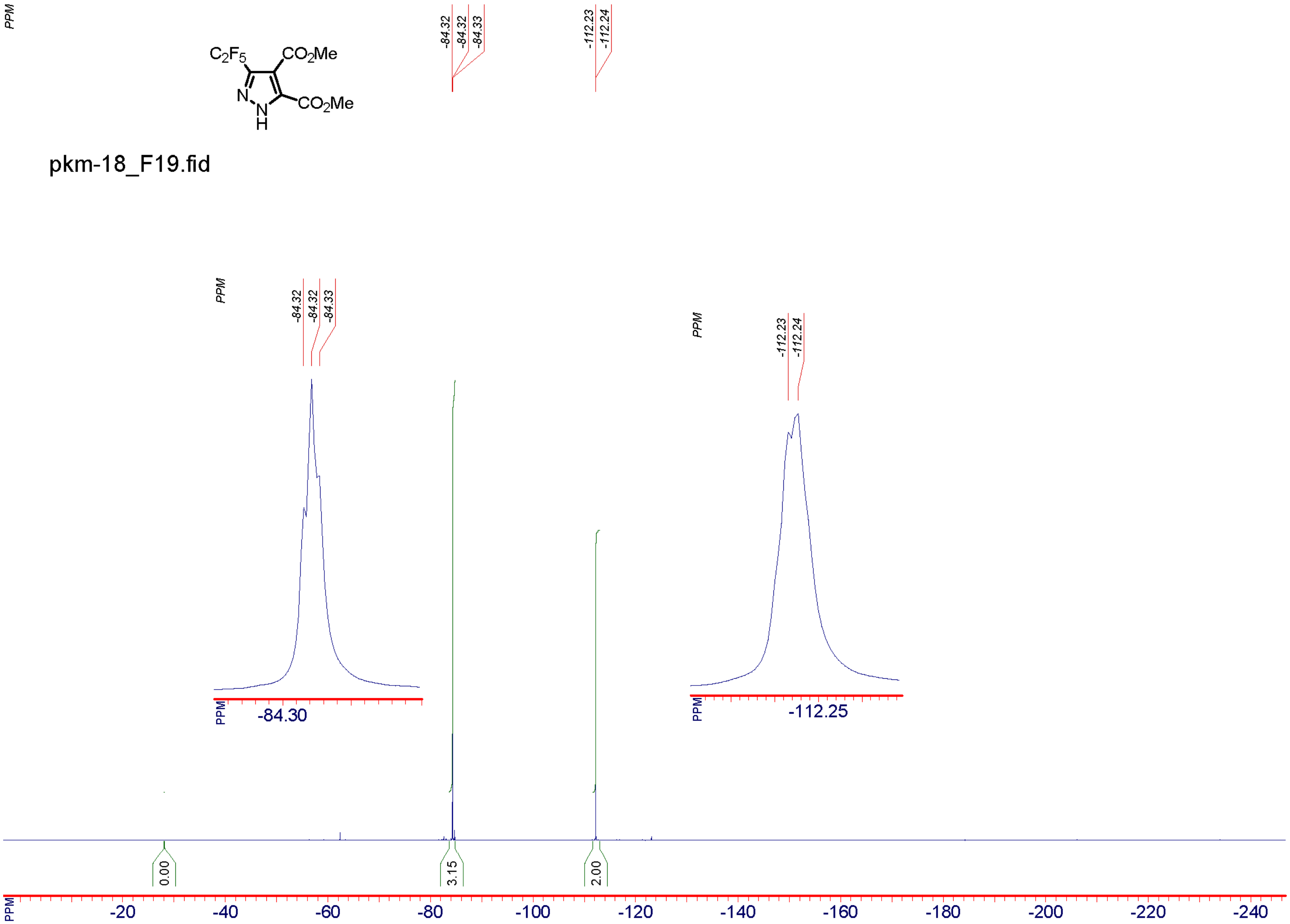

File name: pkm-18_F19.fid	Operator:	SF: 376.3207 MHz	NSC: 0	PW: 3.00 usec, RG: 16	SI: 131072
Date: 18-Jun-2013	Solvent: cdcl3	SW: 94118 Hz	TE: 293 K	AQ: 0.68 sec, RD: 0.00 sec	□

Compound 16a

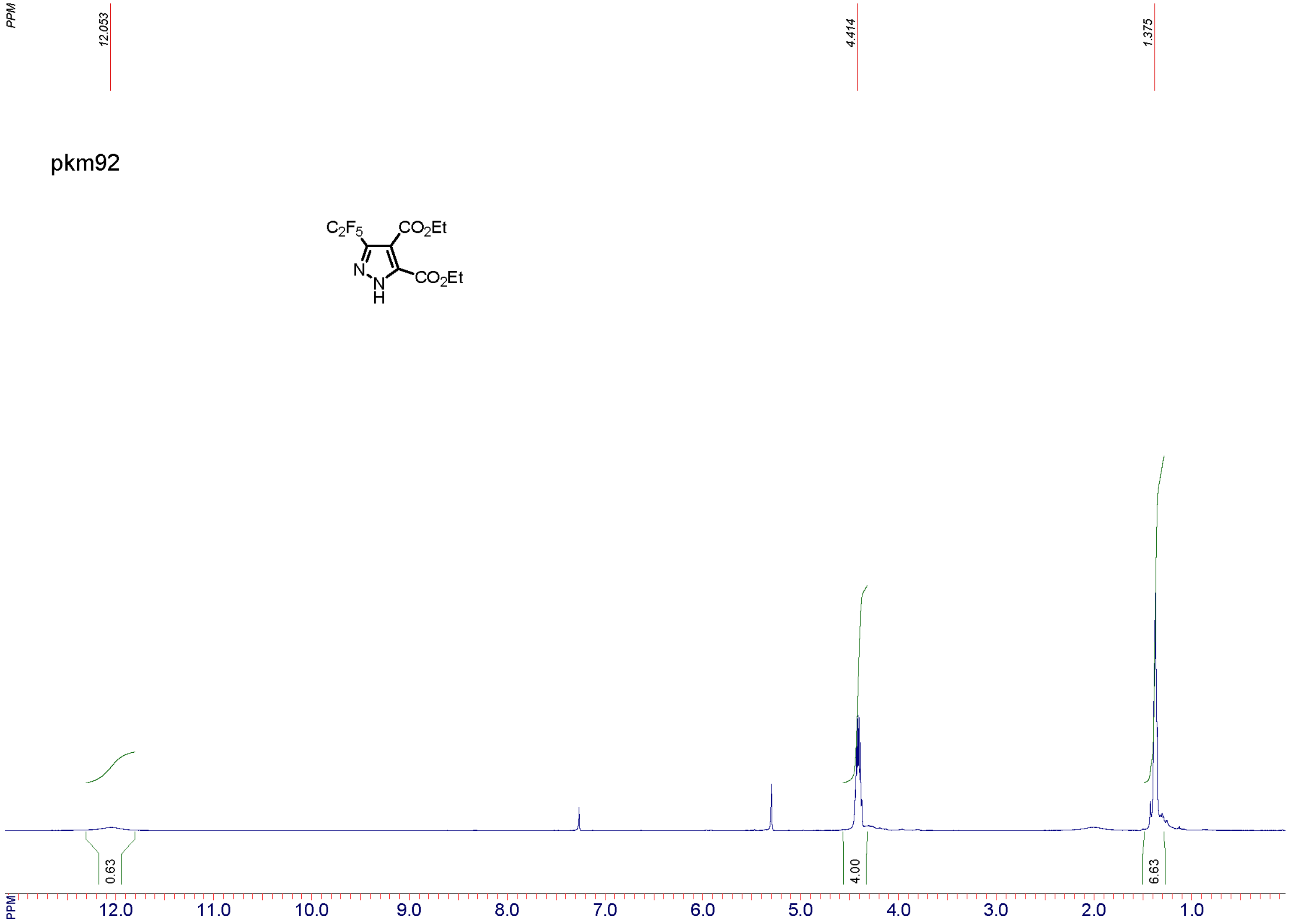

**File name: pkm92**	Operator: root	SF: 499.9500 MHz	NSC: 1	PW: 0.00 usec, RG: 24	SI: 32768
Date: 21-Oct-2013	Solvent: CDCl3	SW: 8993 Hz	TE: 0 K	AQ: 1.82 sec, RD: 0.00 sec	Parameter file, XWIN-NMR Version 3.5

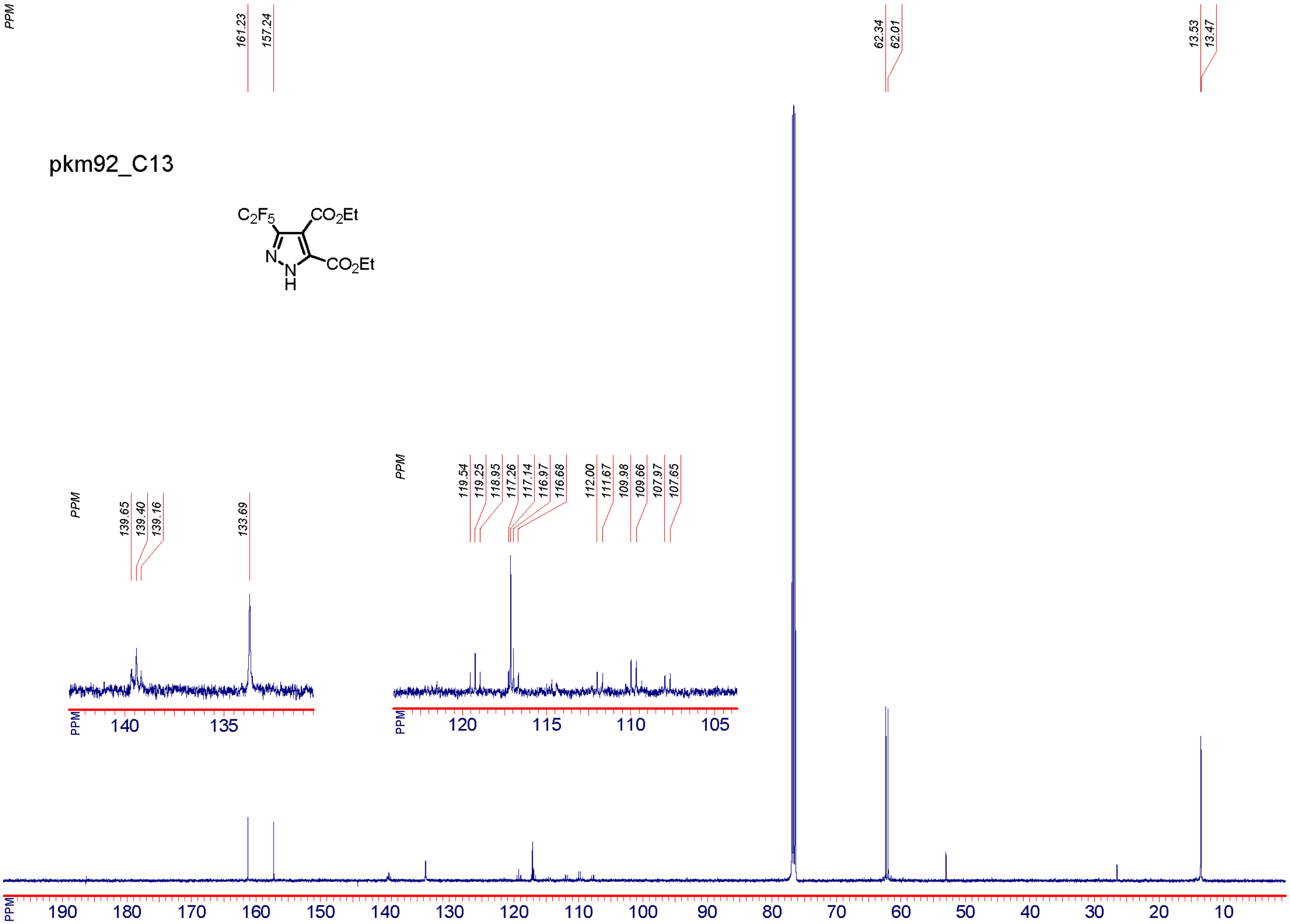

**File name: pkm92_C13**	Operator: root	SF: 125.7126 MHz	NSC: 5120	PW: 0.00 usec, RG: 51200	SI: 65536
Date: 25-Oct-2013	Solvent: CDCl3	SW: 32680 Hz	TE: 0 K	AQ: 1.57 sec, RD: 0.00 sec	Parameter file, XWIN-NMR Version 3.5

pkm92_F19.fid

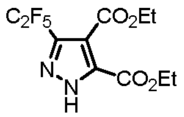

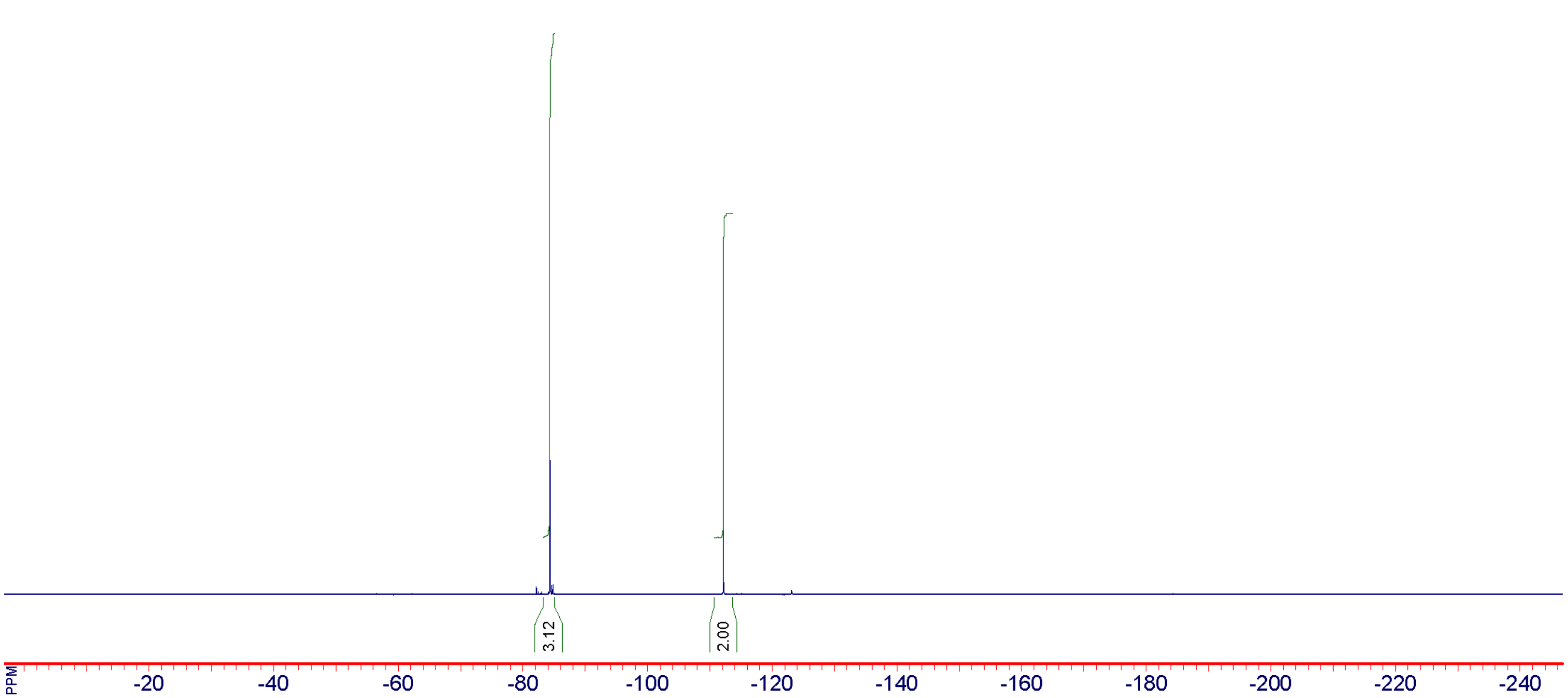

**File name: pkm92_F19.fid**	Operator:	SF: 376.3189 MHz	NSC: 0	PW: 3.00 usec, RG: 16	SI: 131072
Date: 29-Oct-2013	Solvent: cdcl3	SW: 94118 Hz	TE: 293 K	AQ: 0.68 sec, RD: 0.00 sec	⊓

Compound 17a

pkm217_C13

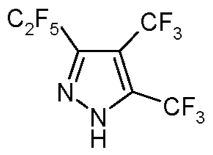

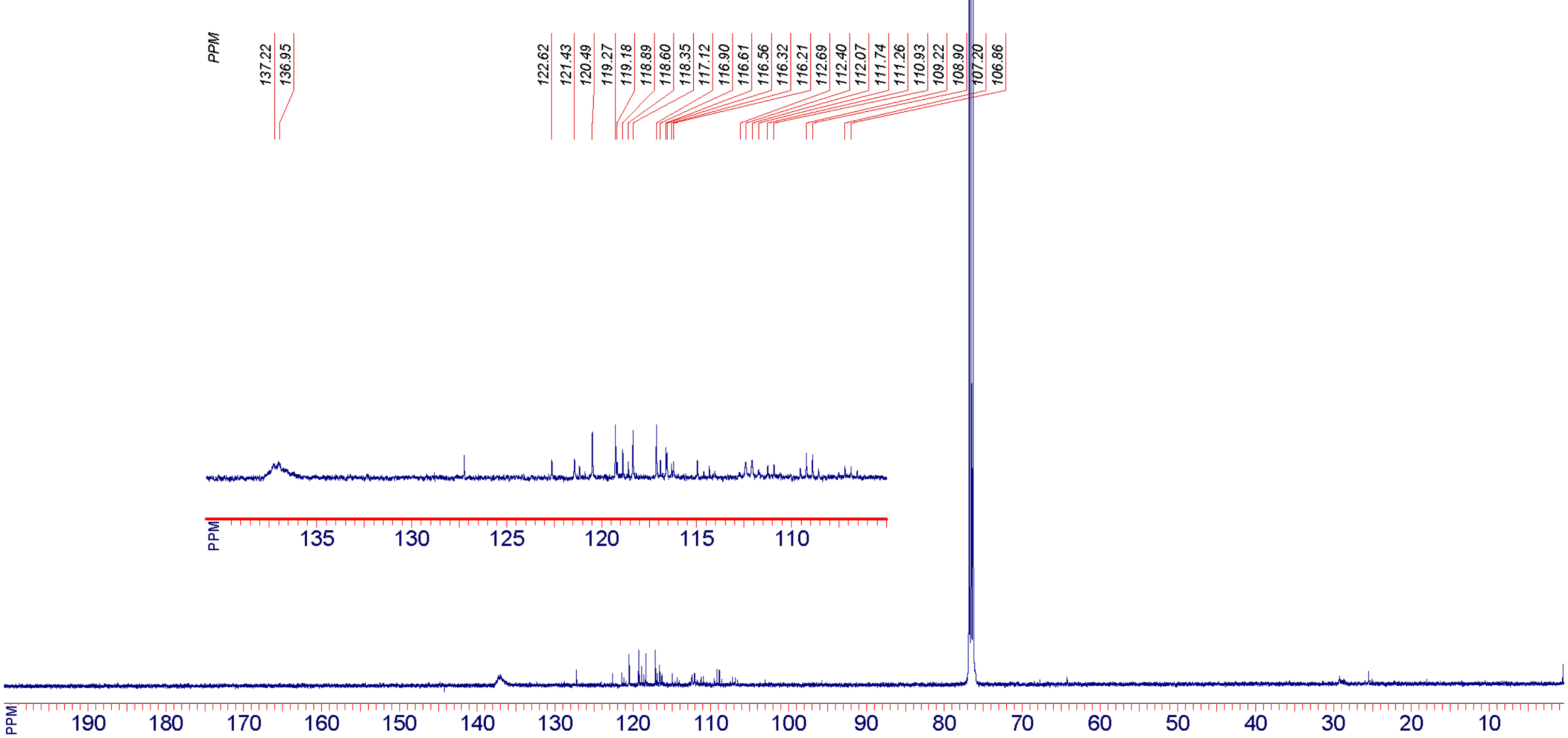

**File name: pkm217_C13**	Operator: root	SF: 125.7126 MHz	NSC: 10240	PW: 0.00 usec, RG: 51200	SI: 65536
Date: 25-May-2014	Solvent: dmso	SW: 32680 Hz	TE: 0 K	AQ: 1.57 sec, RD: 0.00 sec	Parameter file, XWIN-NMR Version 3.5

pkm217_F19.fid

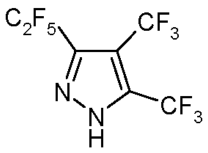

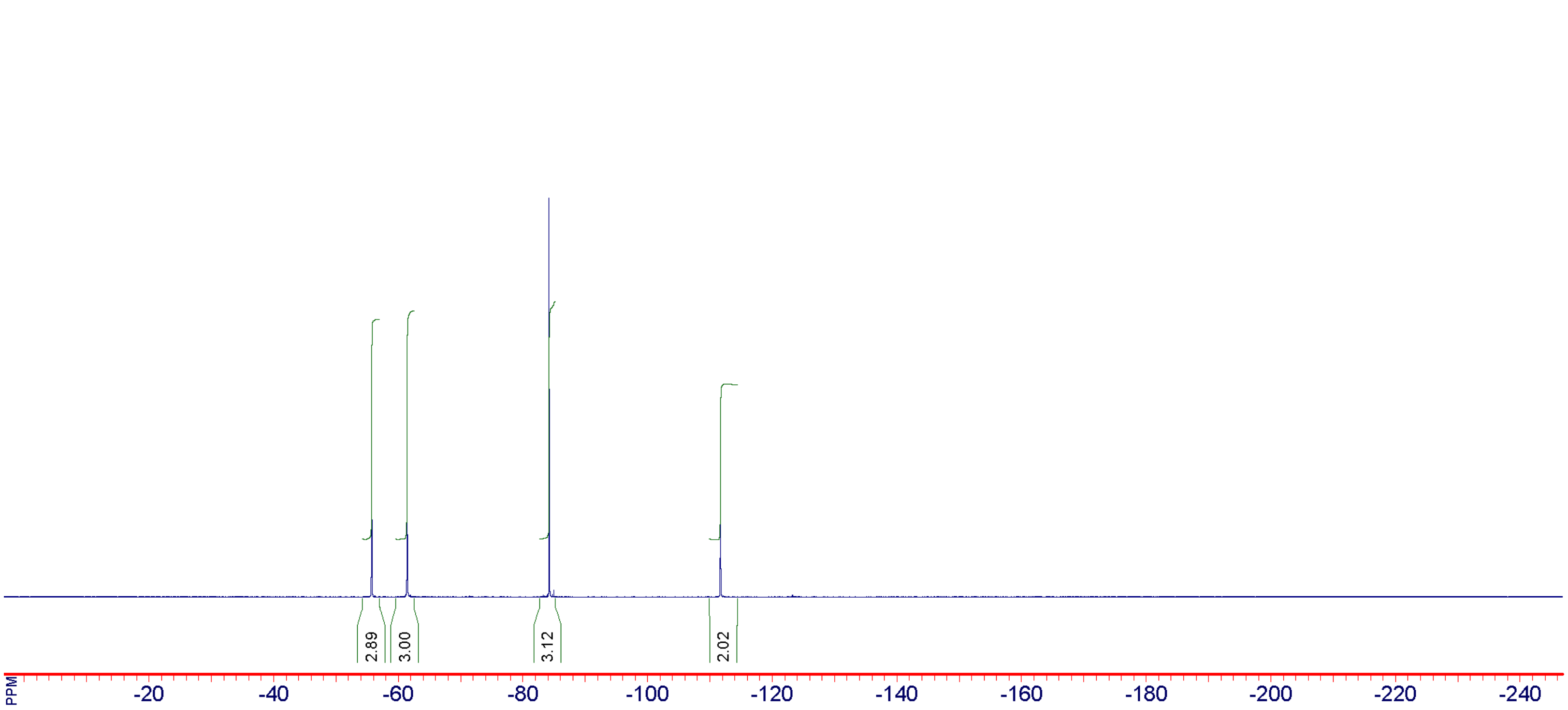

**File name: pkm217_F19.fid**	Operator:	SF: 376.3189 MHz	NSC: 0	PW: 3.00 usec, RG: 32	SI: 65536
Date: 27-May-2014	Solvent: cdcl3	SW: 94118 Hz	TE: 293 K	AQ: 0.34 sec, RD: 0.00 sec	⊓

Compound 18a/18b

pkm113

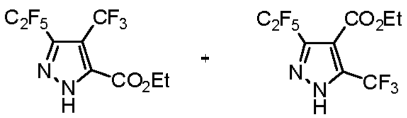

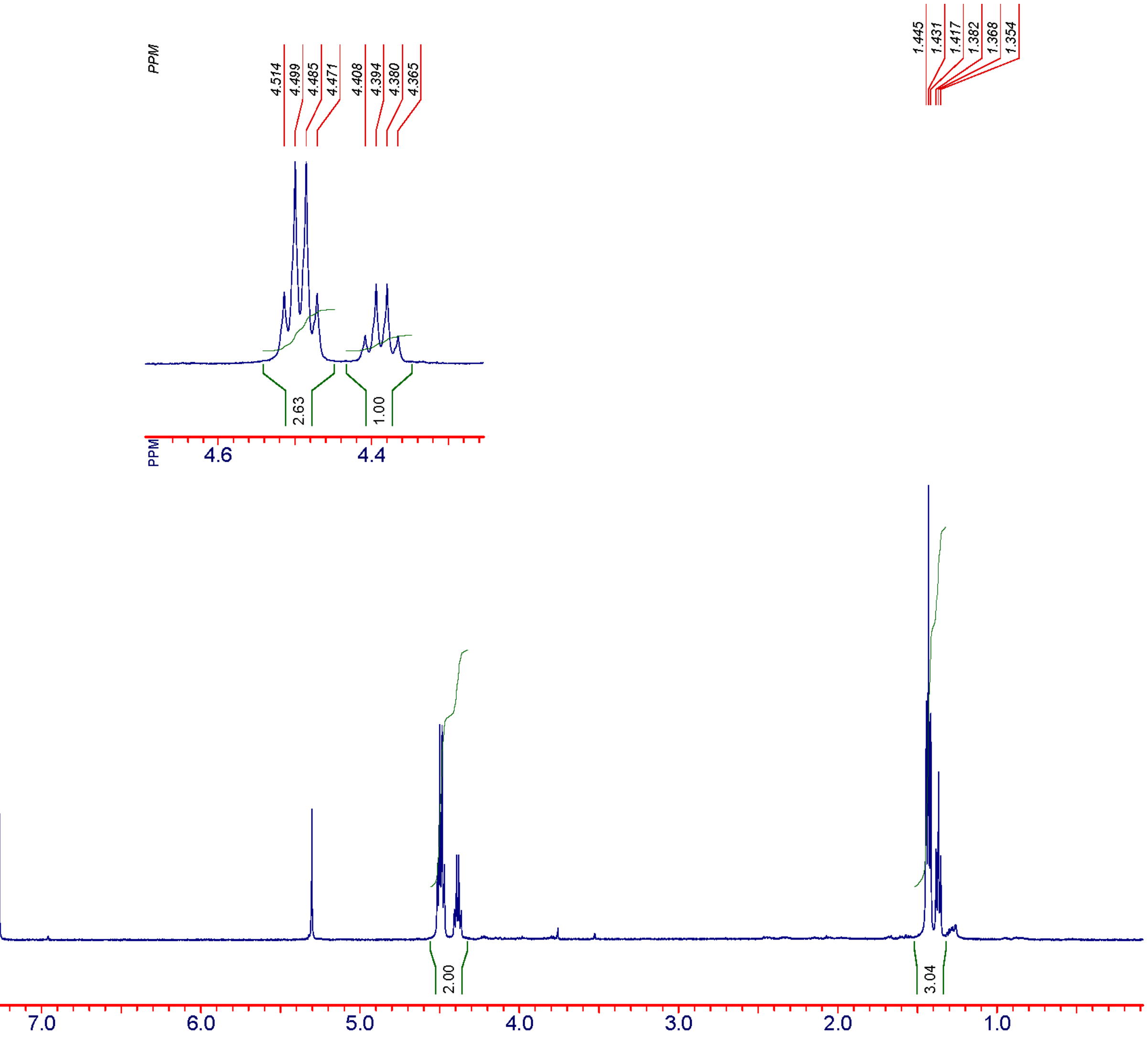

File name: pkm113	Operator: root	SF: 499.9500 MHz	NSC: 1	PW: 0.00 usec, RG: 100	SI: 32768
Date: 21-Nov-2013	Solvent: CDCl3	SW: 8993 Hz	TE: 0 K	AQ: 1.82 sec, RD: 0.00 sec	Parameter file, XWIN-NMRVersion 3.5

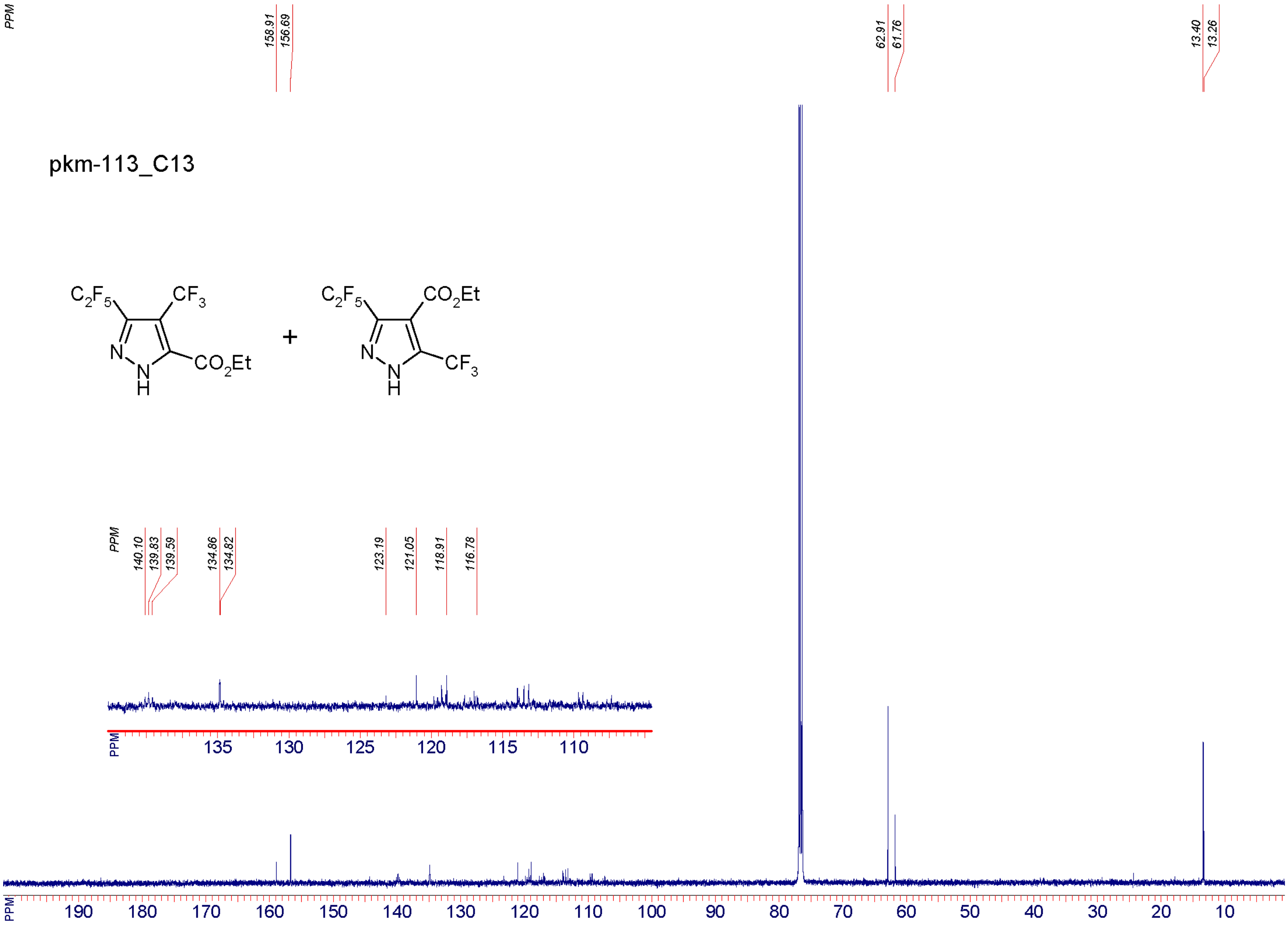

**File name: pkm-113_C13**	Operator: root	SF: 125.7126 MHz	NSC: 5120	PW: 0.00 usec, RG: 51200	SI: 65536
Date: 06-Dec-2013	Solvent: CDCl3	SW: 32680 Hz	TE: 0 K	AQ: 1.57 sec, RD: 0.00 sec	Parameter file, XWIN-NMR Version 3.5

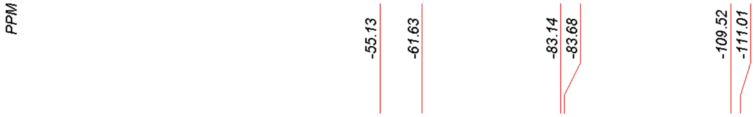

pokm113_F19.fid

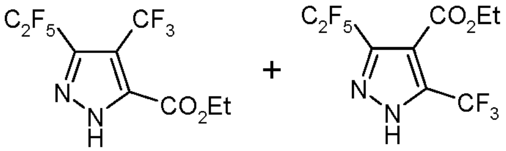

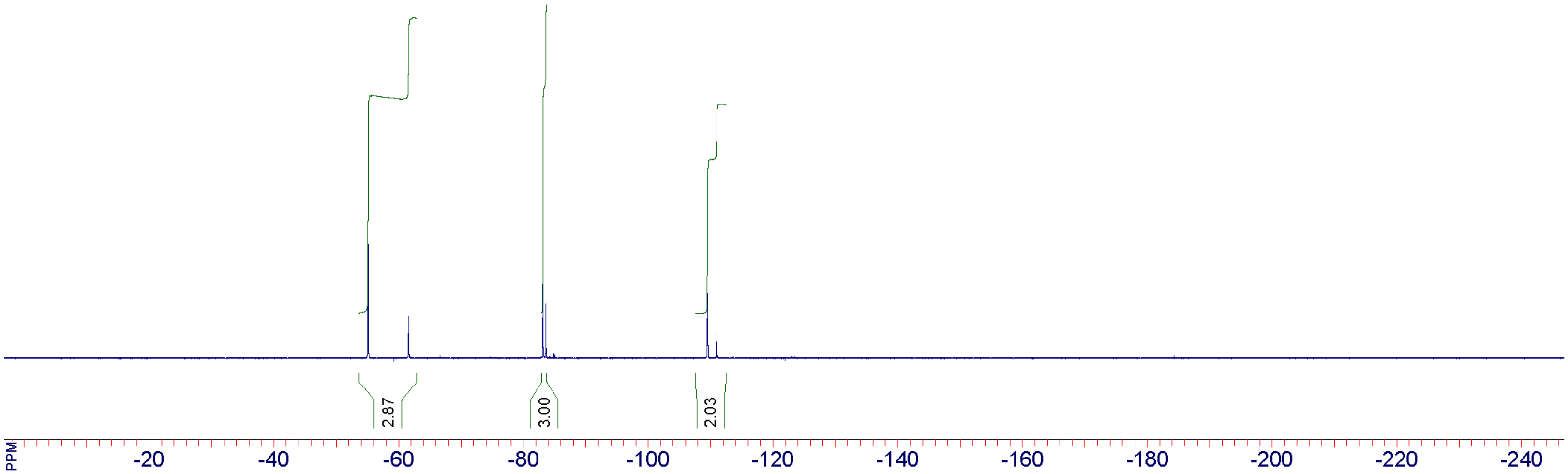

**File name: pokm113_F19.fid**	Operator:	SF: 376.3189 MHz	NSC: 0	PW: 3.00 usec, RG: 16	SI: 131072
Date: 10-Dec-2013	Solvent: cdcl3	SW: 94118 Hz	TE: 293 K	AQ: 0.68 sec, RD: 0.00 sec	⊓

Compound 19b

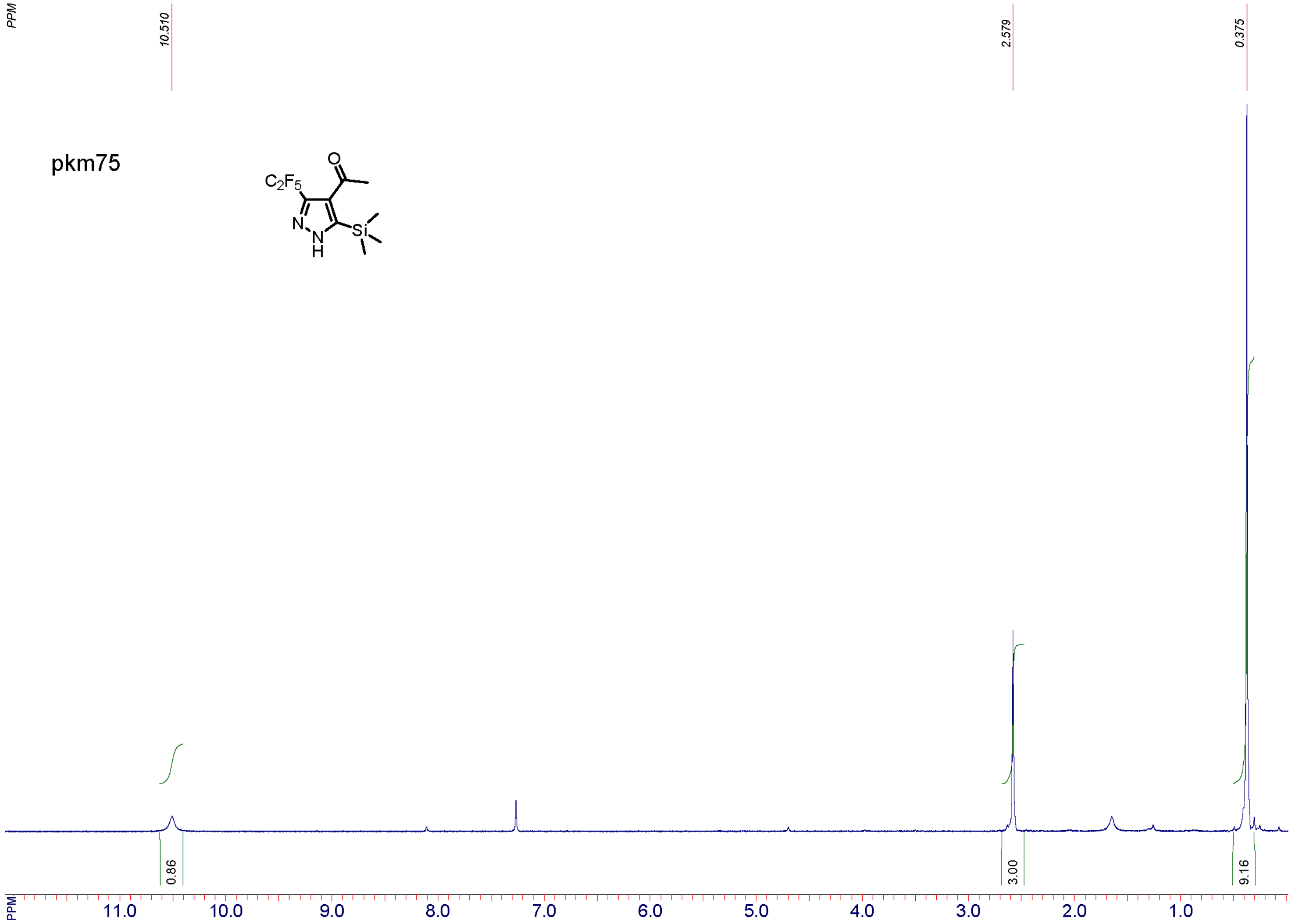

**File name: pkm75**	Operator: root	SF: 499.9500 MHz	NSC: 1	PW: 0.00 usec, RG: 16	SI: 32768
Date: 03-Oct-2013	Solvent: CDCl3	SW: 8993 Hz	TE: 0 K	AQ: 1.82 sec, RD: 0.00 sec	Parameter file, XWIN-NMR Version 3.5

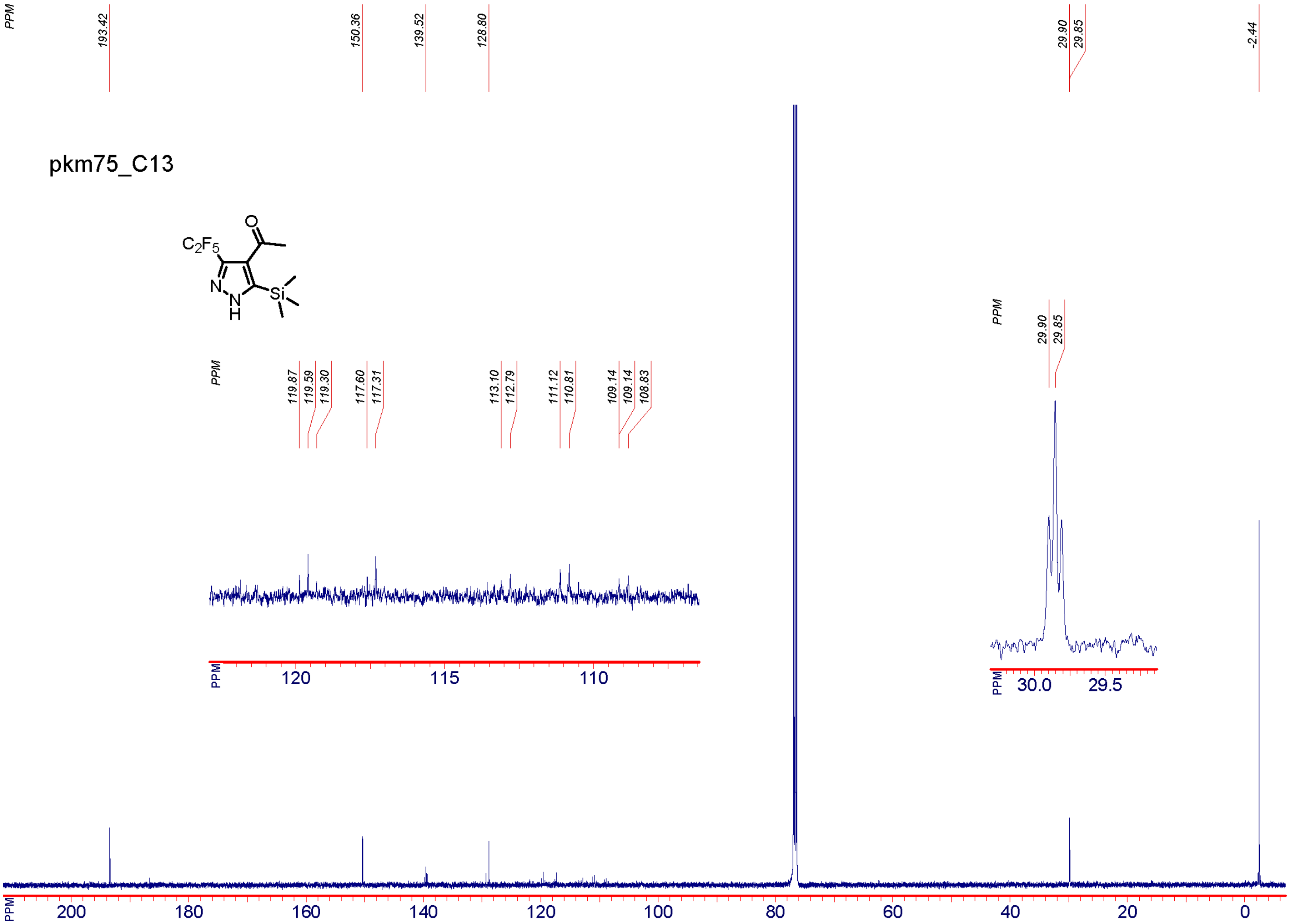

File name: pkm75_C13	Operator: root	SF: 125.7126 MHz	NSC: 7696	PW: 0.00 usec, RG: 51200	SI: 65536
Date: 03-Oct-2013	Solvent: CDCl3	SW: 32680 Hz	TE: 0 K	AQ: 1.57 sec, RD: 0.00 sec	Parameter file, XWIN-NMR Version 3.5

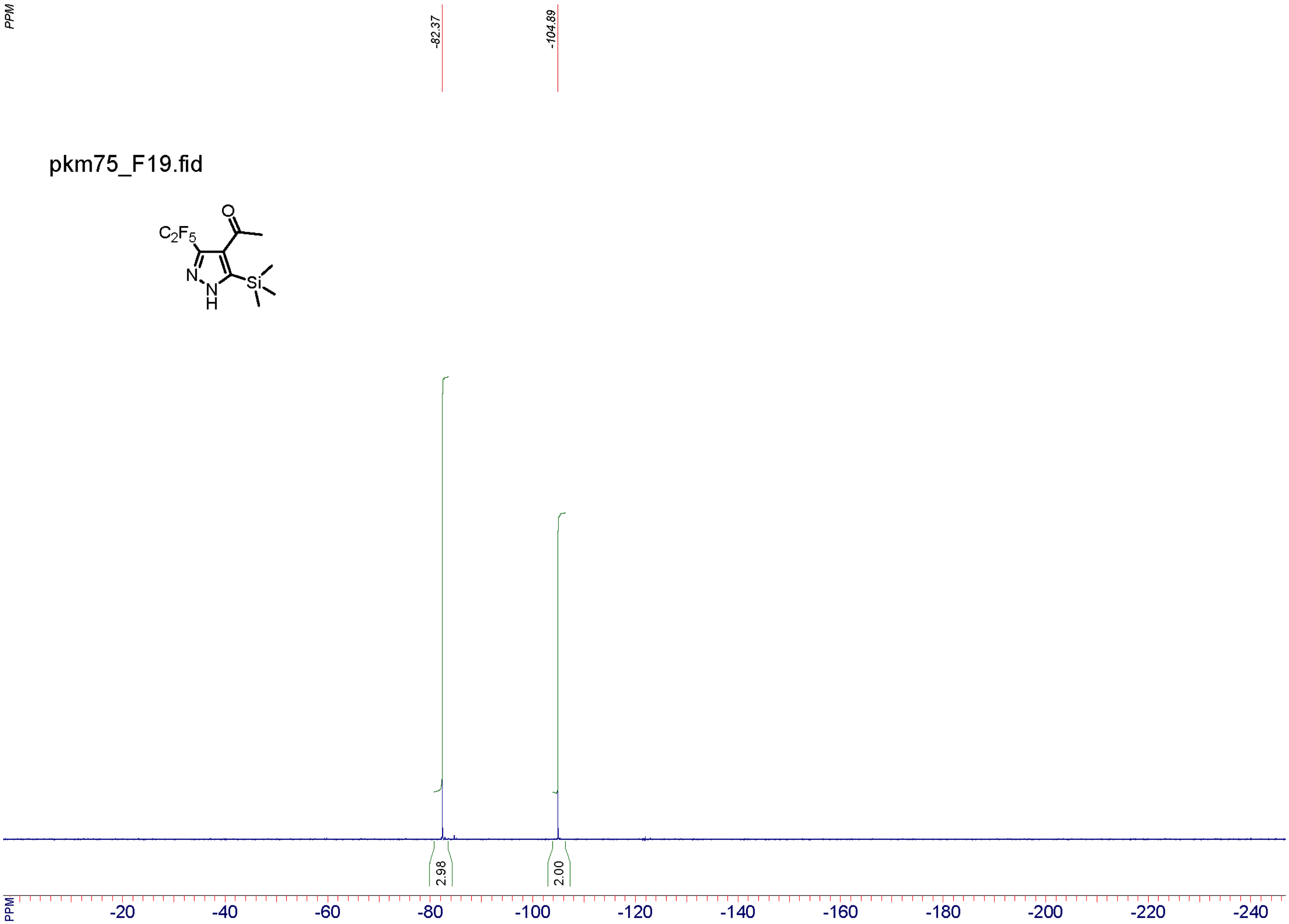

**File name: pkm75_F19.fid**	Operator:	SF: 376.3189 MHz	NSC: 0	PW: 3.00 usec, RG: 16	SI: 131072
Date: 08-Oct-2013	Solvent: cdcl3	SW: 94118 Hz	TE: 293 K	AQ: 0.68 sec, RD: 0.00 sec	⊓

Compound 20b

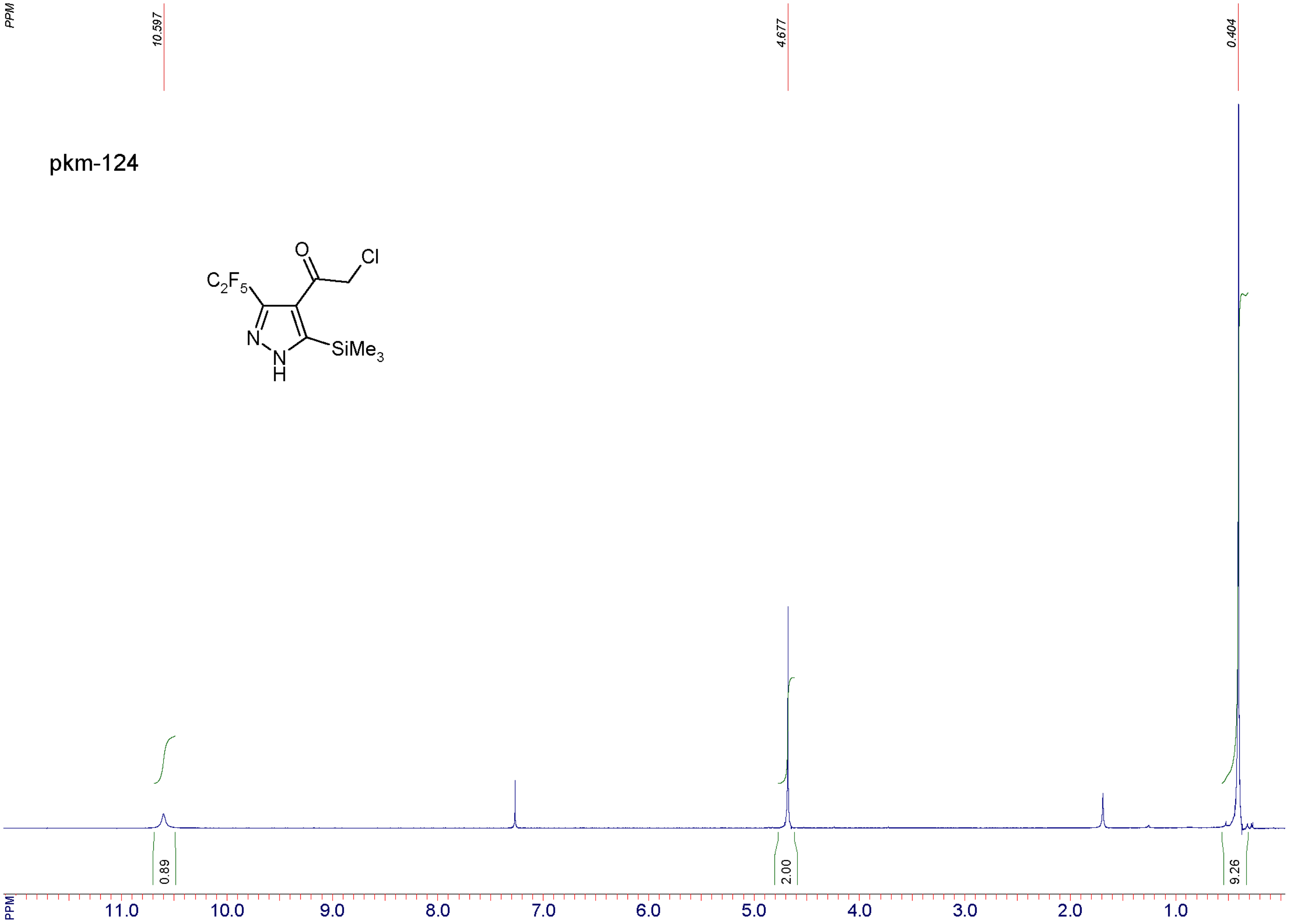

File name: pkm-124	Operator: root	SF: 499.9500 MHz	NSC: 1	PW: 0.00 usec, RG: 50	SI: 32768
Date: 12-Dec-2013	Solvent: CDCl3	SW: 8993 Hz	TE: 0 K	AQ: 1.82 sec, RD: 0.00 sec	Parameter file, XWIN-NMR Version 3.5

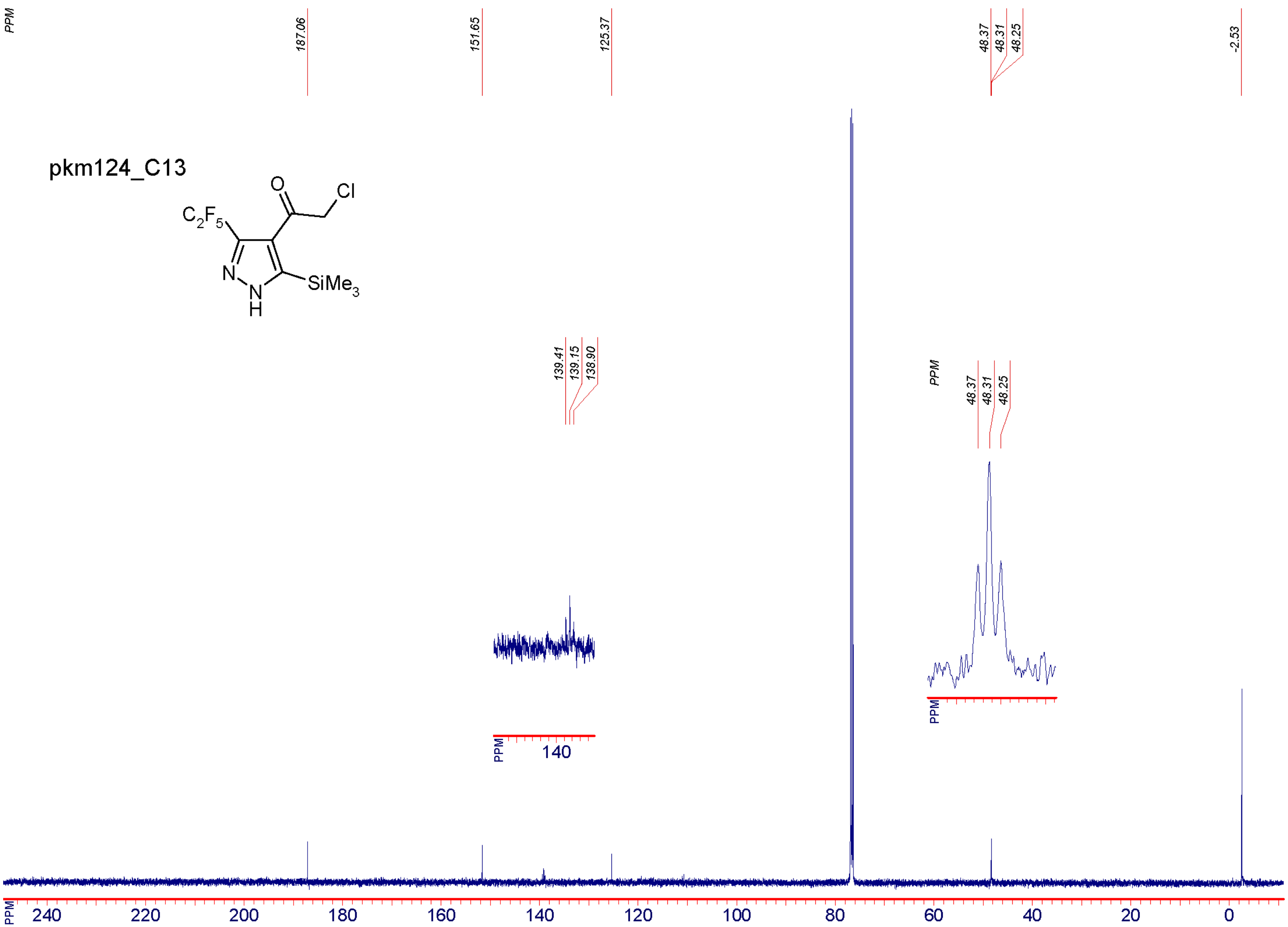

**File name: pkm124_C13**	Operator: root	SF: 125.7126 MHz	NSC: 1154	PW: 0.00 usec, RG: 51200	SI: 65536
Date: 13-Dec-2013	Solvent: dmso	SW: 32680 Hz	TE: 0 K	AQ: 1.57 sec, RD: 0.00 sec	Parameter file, XWIN-NMR Version 3.5

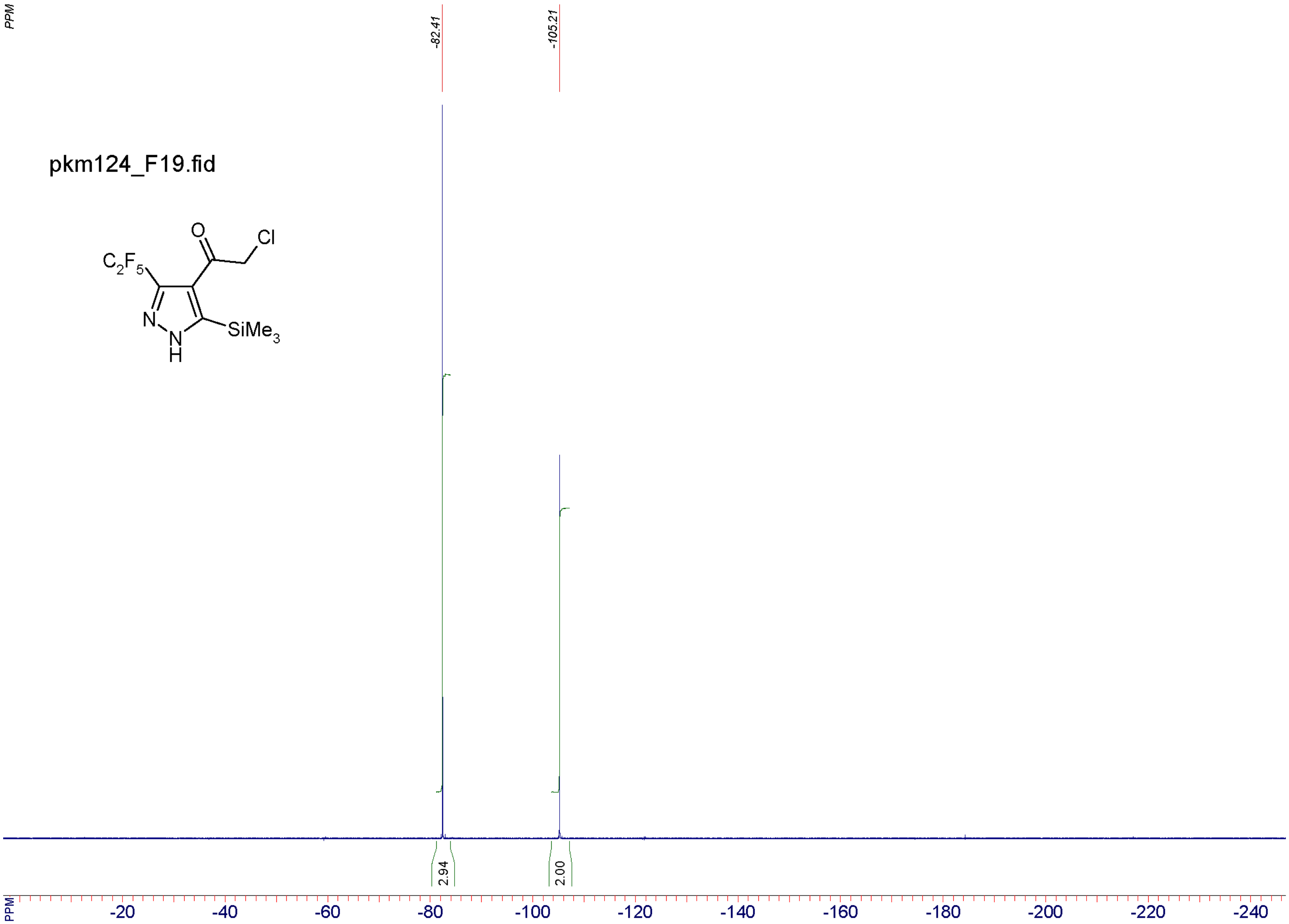

**File name: pkm124_F19.fid**	Operator:	SF: 376.3189 MHz	NSC: 0	PW: 3.00 usec, RG: 16	SI: 131072
Date: 17-Dec-2013	Solvent: cdcl3	SW: 94118 Hz	TE: 293 K	AQ: 0.68 sec, RD: 0.00 sec	⊓

Compound 21a/b

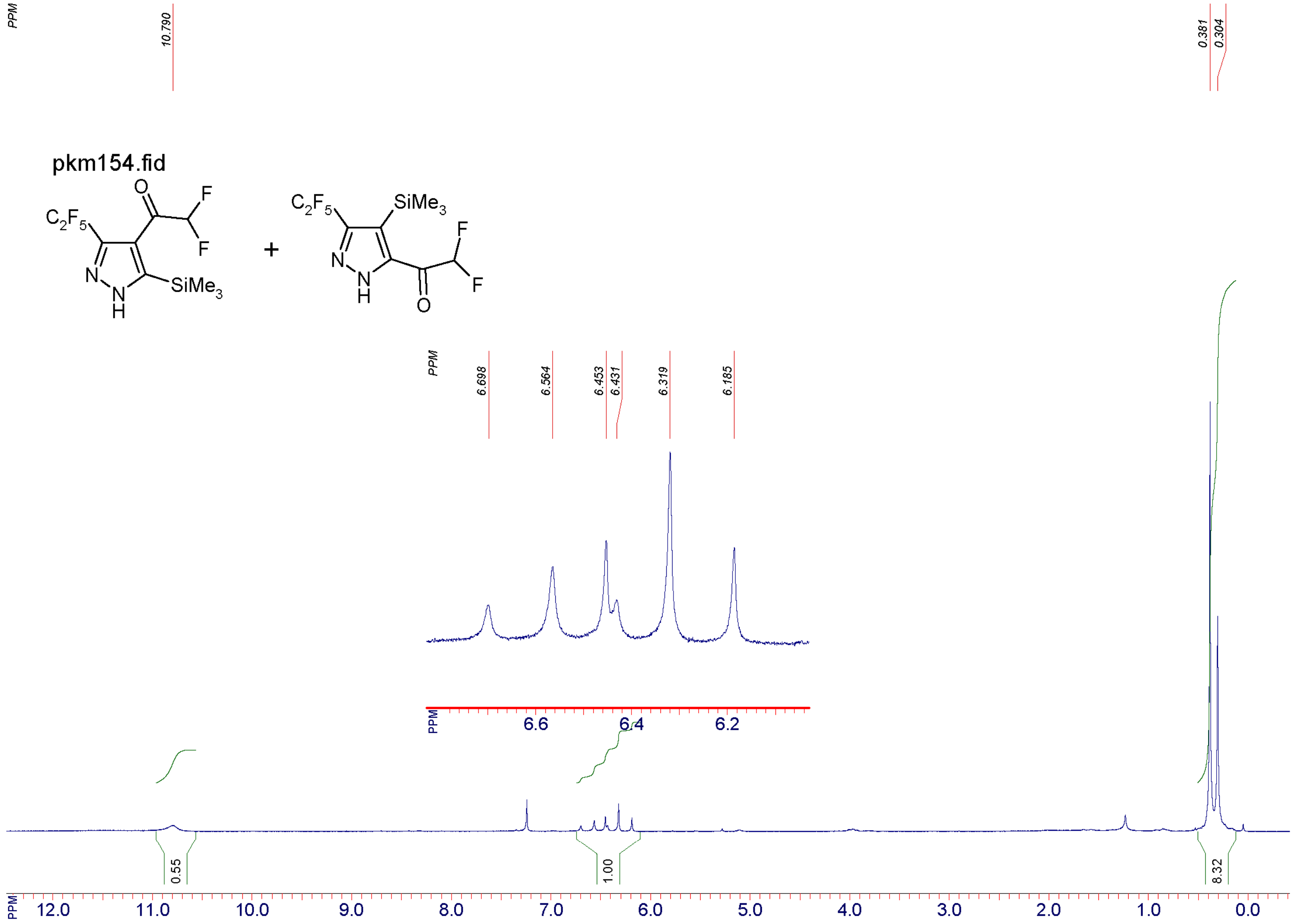

**File name: pkm154.fid**	Operator:	SF: 400.0002 MHz	NSC: 0	PW: 7.50 usec, RG: 26	SI: 32768
Date: 31-Jan-2014	Solvent: CDCl3	SW: 7261 Hz	TE: 293 K	AQ: 1.24 sec, RD: 0.00 sec	⊓

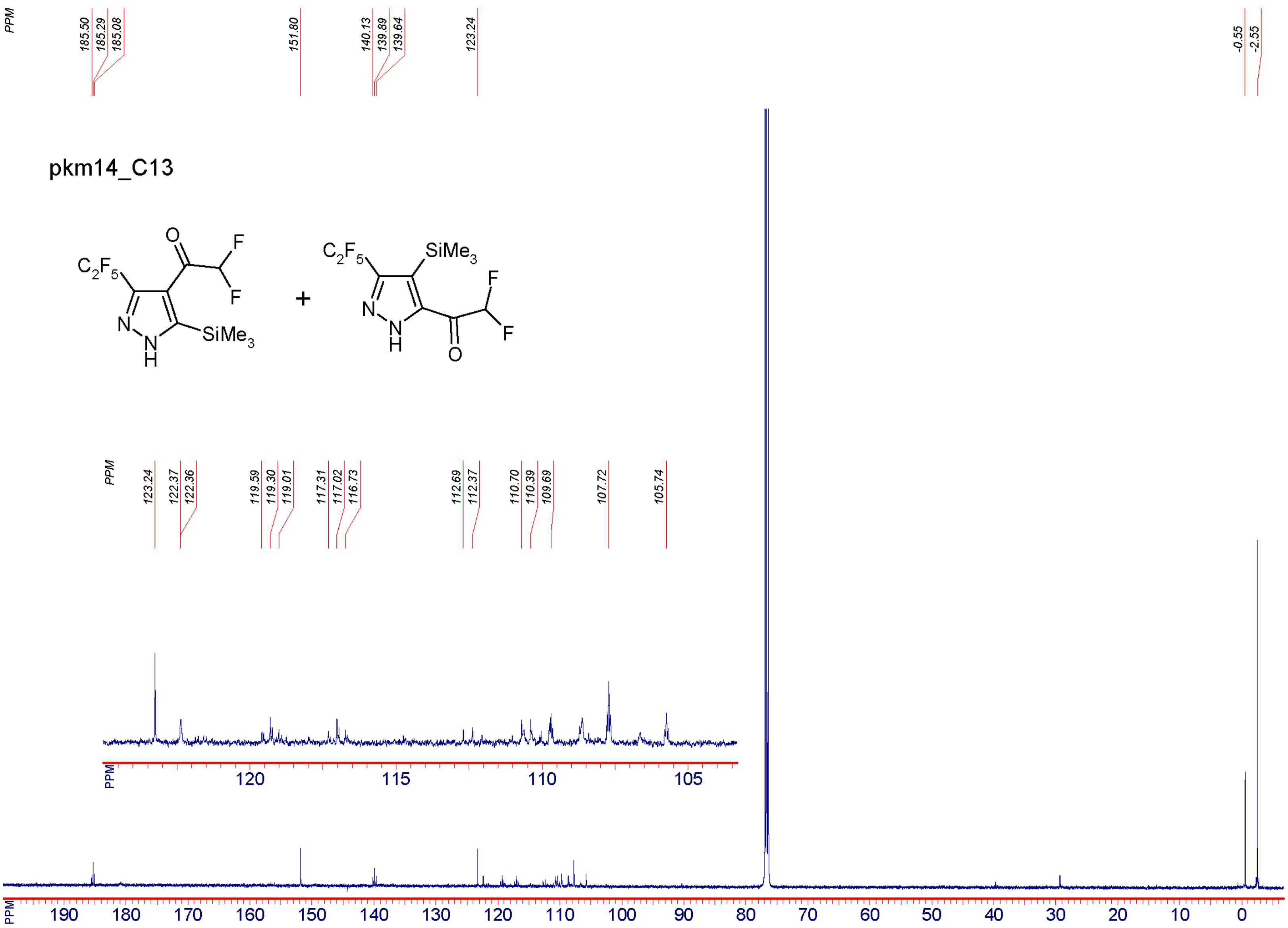

**File name: pkm14_C13**	Operator: root	SF: 125.7126 MHz	NSC: 14831	PW: 0.00 usec, RG: 51200	SI: 65536
Date: 03-Feb-2014	Solvent: CDCl3	SW: 32680 Hz	TE: 0 K	AQ: 1.57 sec, RD: 0.00 sec	Parameter file, XWIN-NMR Version 3.5

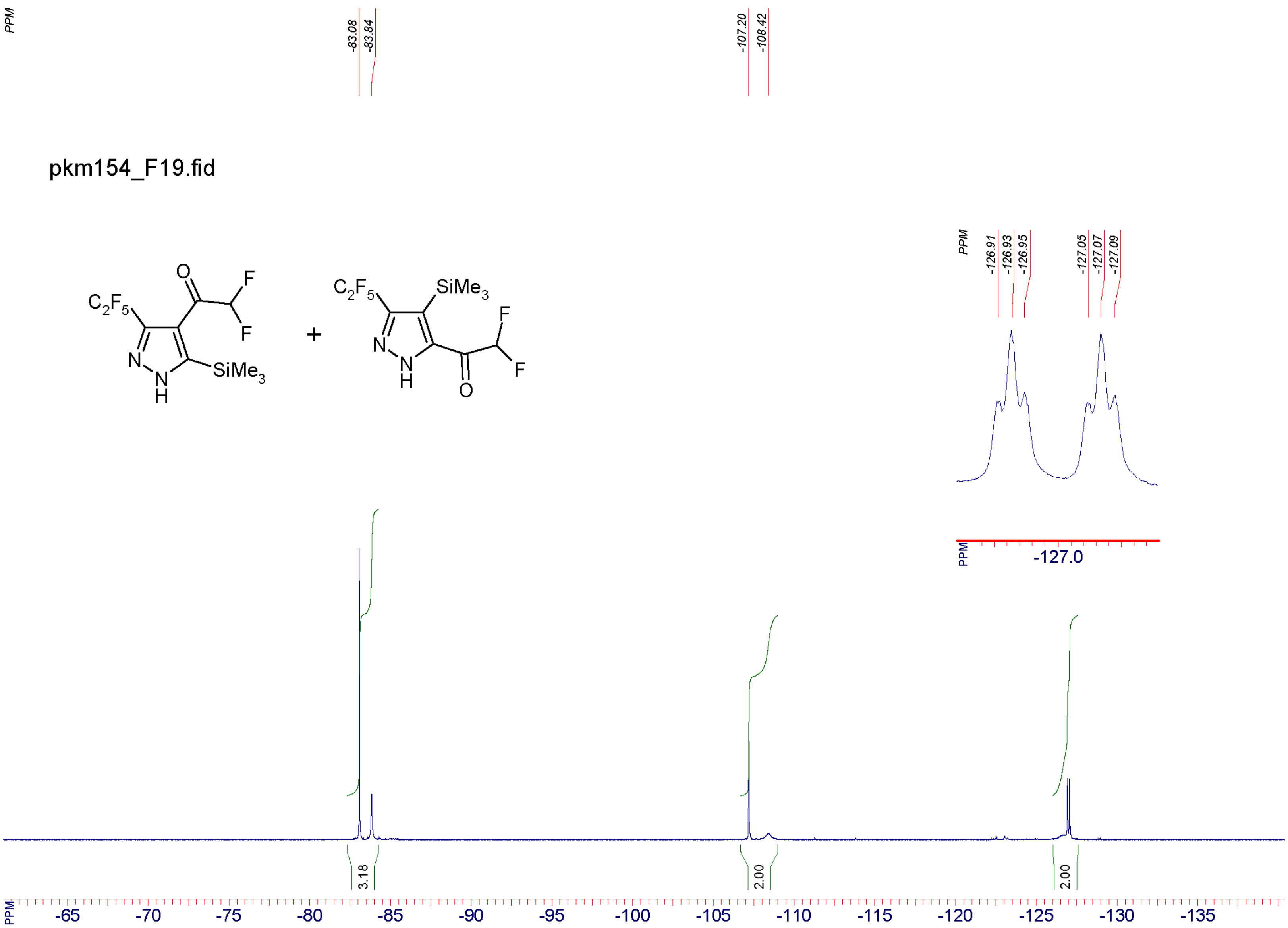

**File name: pkm154_F19.fid**	Operator:	SF: 376.3189 MHz	NSC: 0	PW: 3.00 usec, RG: 16	SI: 131072
Date: 04-Feb-2014	Solvent: cdcl3	SW: 94118 Hz	TE: 293 K	AQ: 0.68 sec, RD: 0.00 sec	

Compound 23

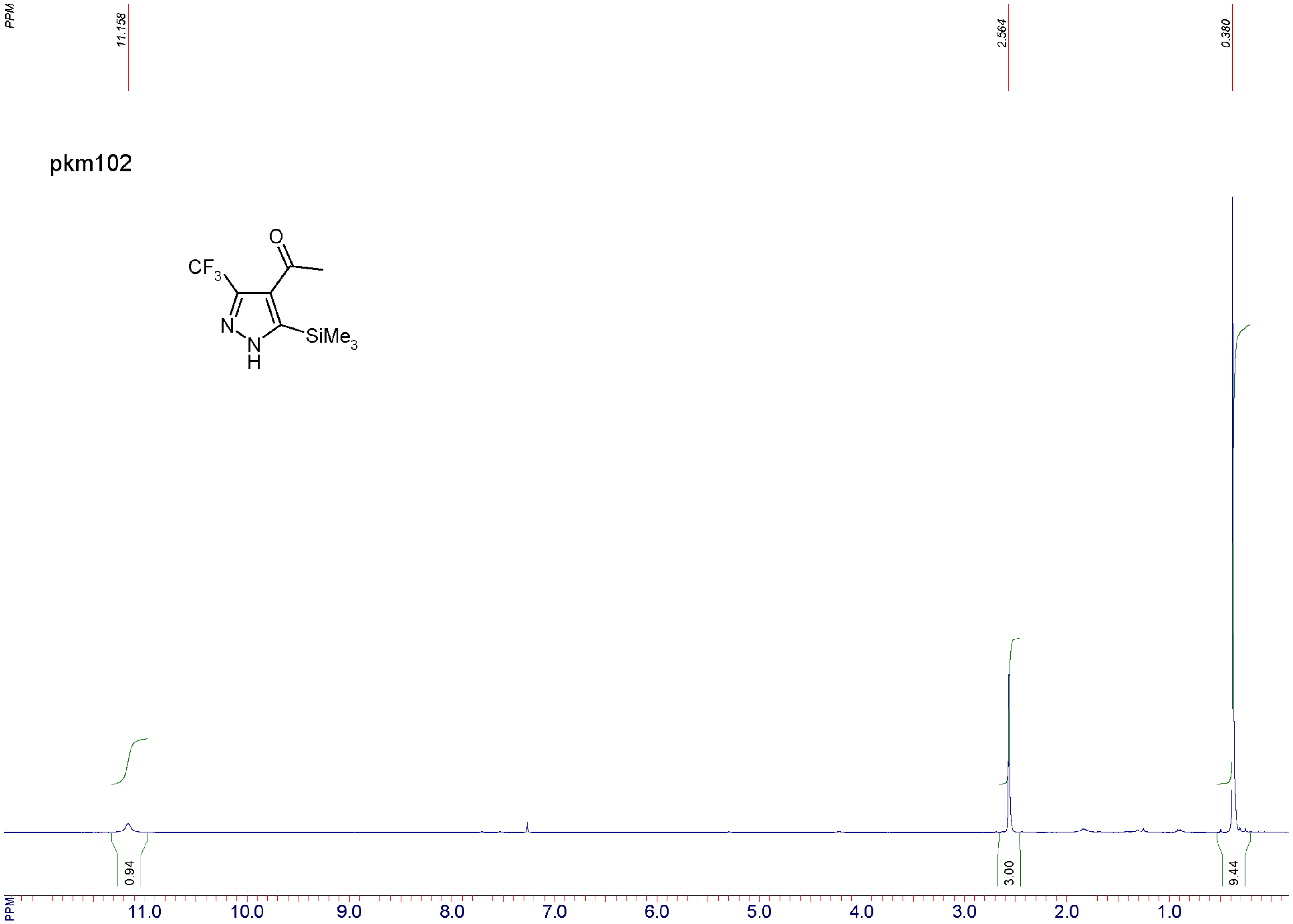

**File name: pkm102**	Operator: root	SF: 499.9500 MHz	NSC: 1	PW: 0.00 usec, RG: 32	SI: 32768
Date: 31-Oct-2013	Solvent: DMSO	SW: 8993 Hz	TE: 0 K	AQ: 1.82 sec, RD: 0.00 sec	Parameter file, XWIN-NMR Version 3.5

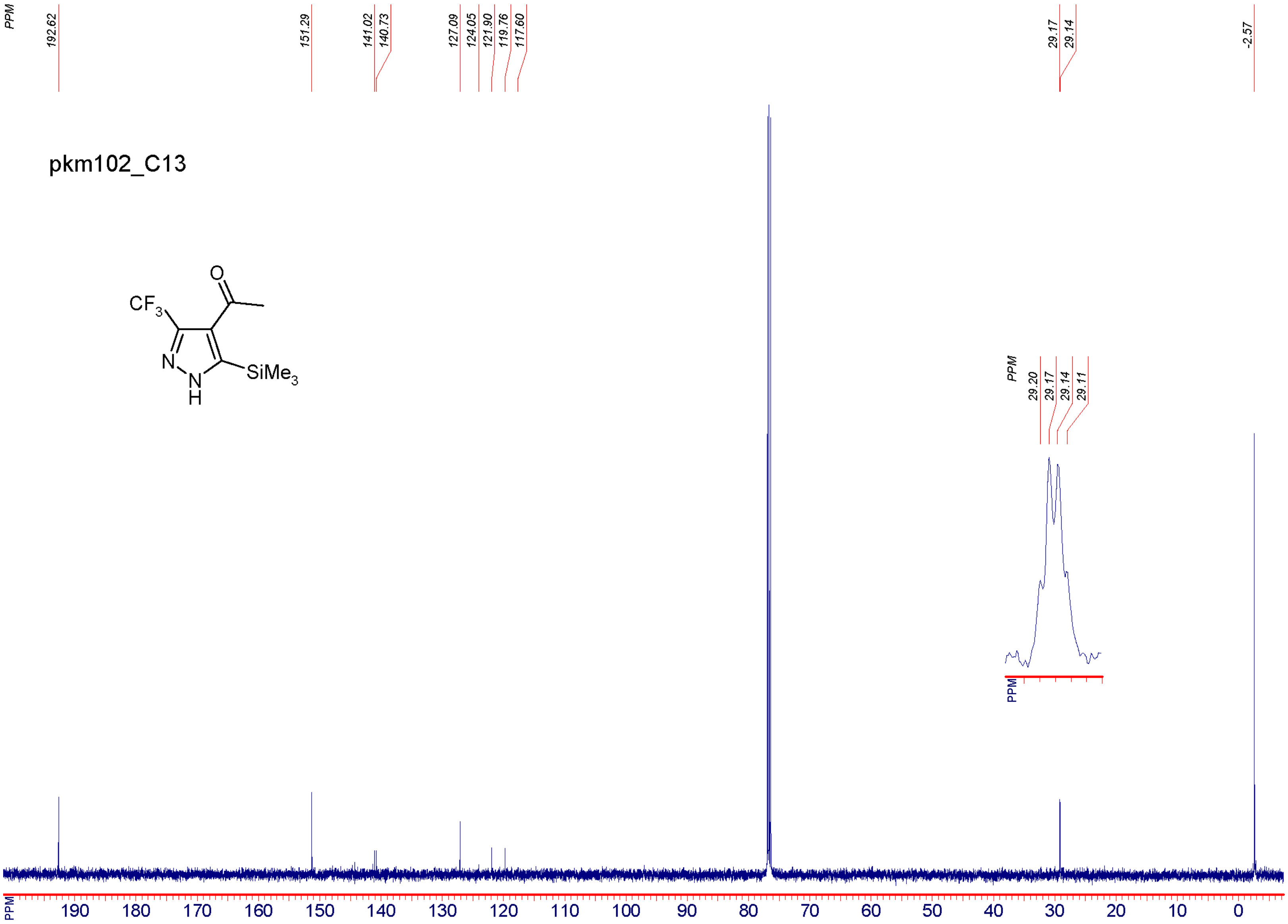

**File name: pkm102_C13**	Operator: root	SF: 125.7126 MHz	NSC: 431	PW: 0.00 usec, RG: 51200	SI: 65536
Date: 31-Oct-2013	Solvent: dmso	SW: 32680 Hz	TE: 0 K	AQ: 1.57 sec, RD: 0.00 sec	Parameter file, XWIN-NMR Version 3.5

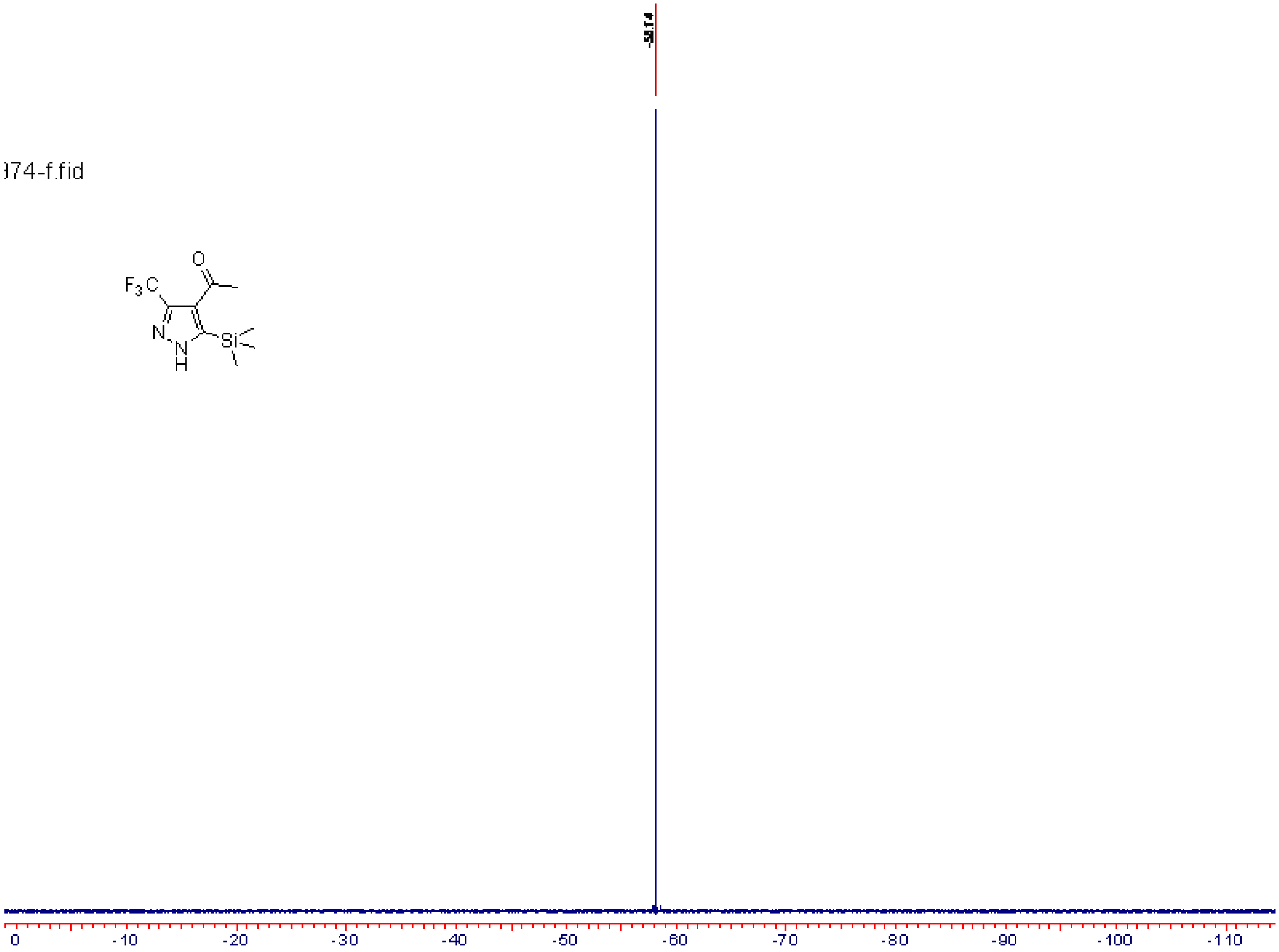

74-f.fid
O
F3C
N
N
H
Si
0
-10
-20
-30
-40
-50
-60
-70
-80
-90
-100
-110

Compound 24

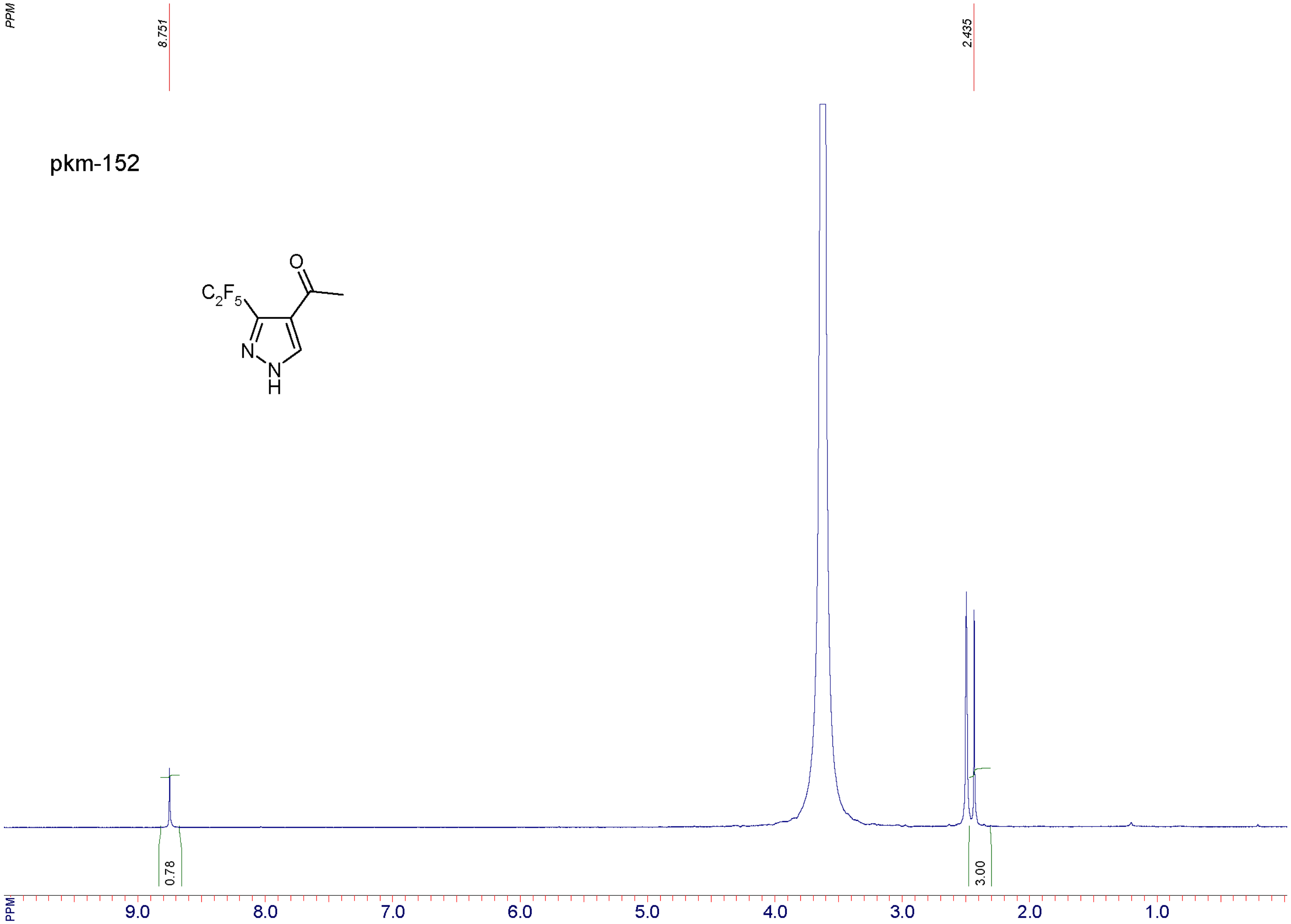

File name: pkm-152	Operator: root	SF: 499.9500 MHz	NSC: 1	PW: 0.00 usec, RG: 32	SI: 32768
Date: 28-Jan-2014	Solvent: DMSO	SW: 8993 Hz	TE: 0 K	AQ: 1.82 sec, RD: 0.00 sec	Parameter file, XWIN-NMR Version 3.5

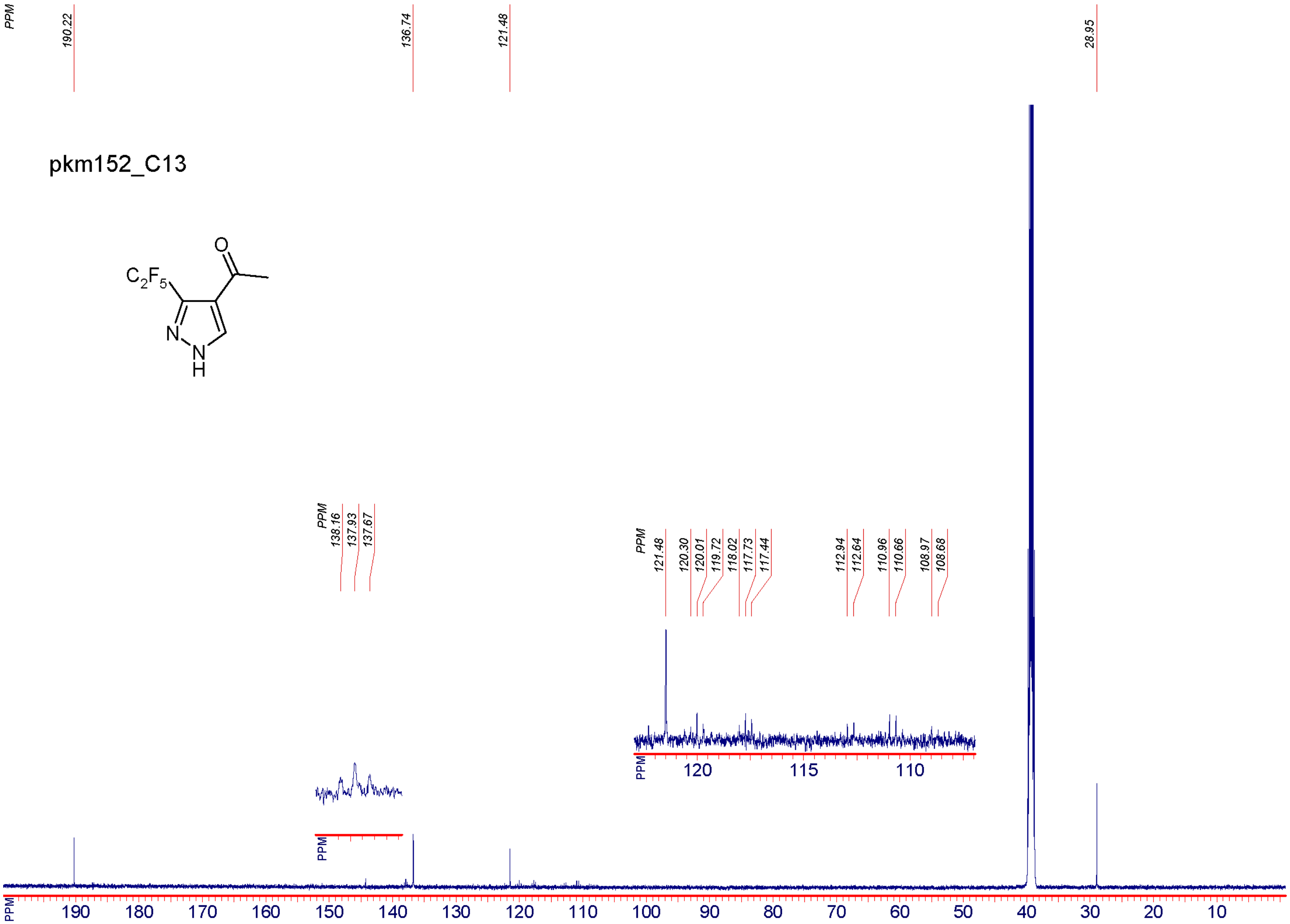

File name: pkm152_C13	Operator: root	SF: 125.7126 MHz	NSC: 10240	PW: 0.00 usec, RG: 51200	SI: 65536
Date: 31-Jan-2014	Solvent: dmso	SW: 32680 Hz	TE: 0 K	AQ: 1.57 sec, RD: 0.00 sec	Parameter file, XWIN-NMR Version 3.5

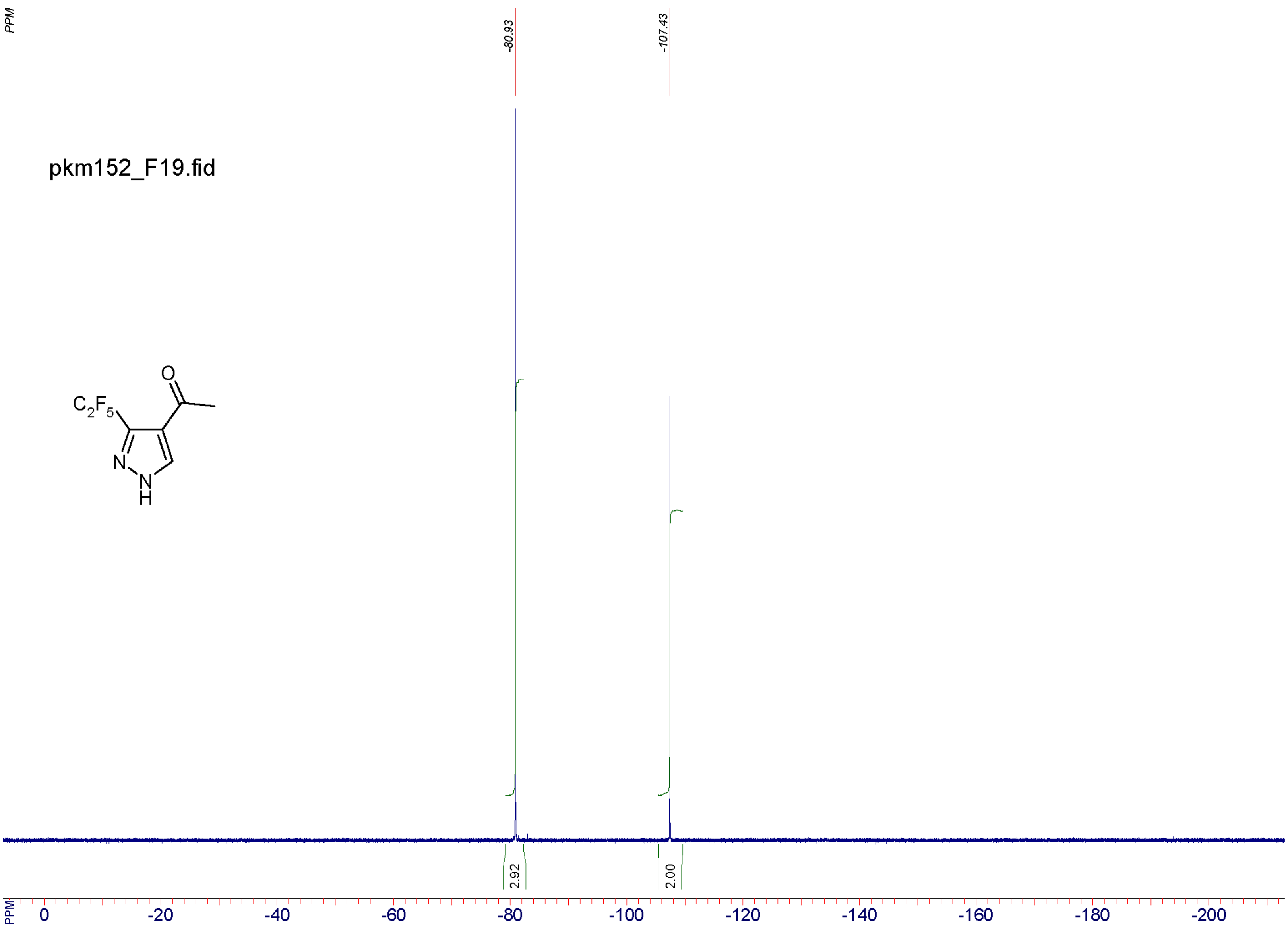

**File name: pkm152_F19.fid**	Operator:	SF: 376.3274 MHz	NSC: 0	PW: 3.00 usec, RG: 22	SI: 65536
Date: 04-Feb-2014	Solvent: dmso_d6	SW: 82816 Hz	TE: 293 K	AQ: 0.39 sec, RD: 0.00 sec	⊓

Compound 25

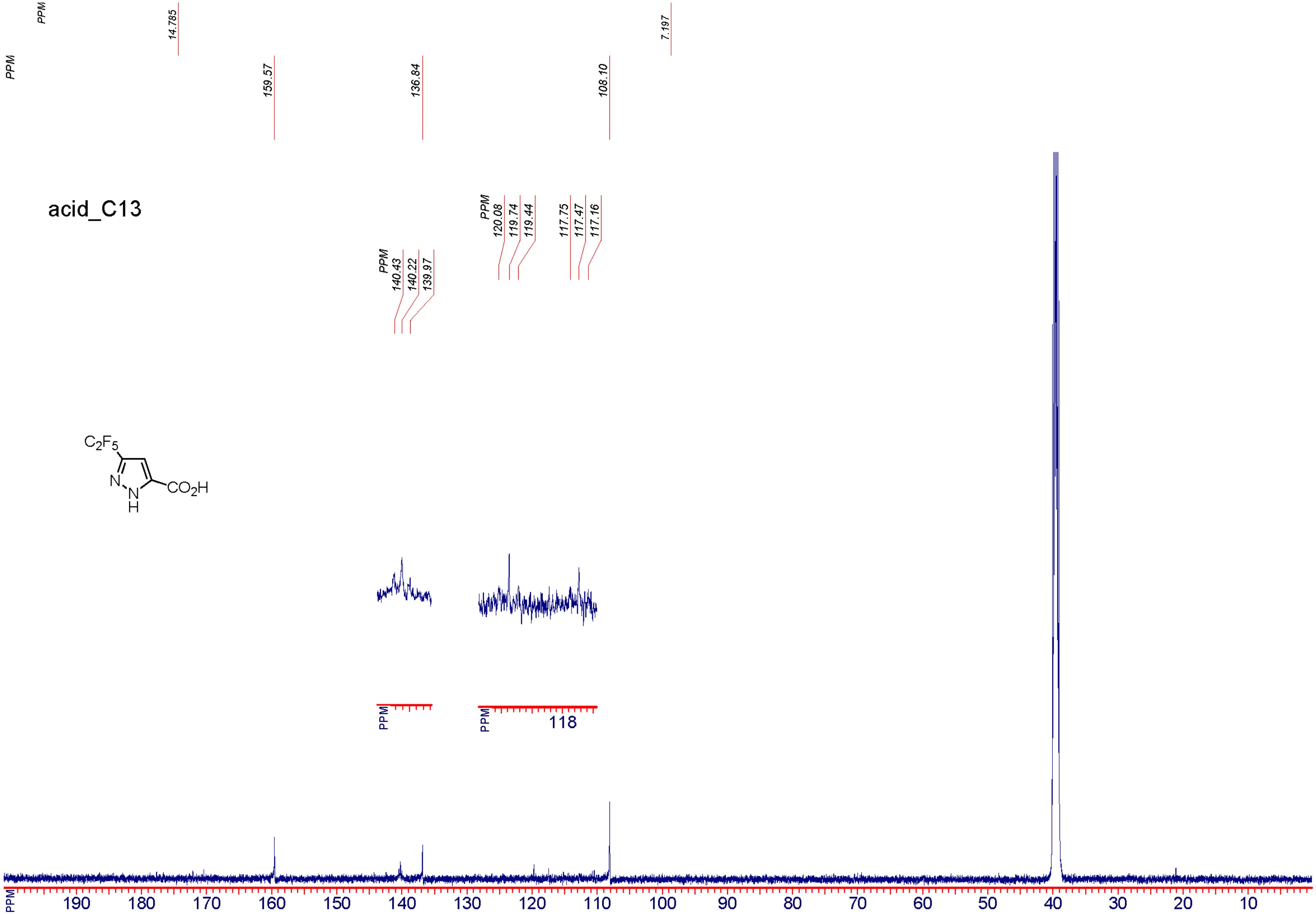

File name: acid_C13	Operator: root	SF: 125.7126 MHz	NSC: 5120	PW: 0.00 usec, RG: 51200	SI: 65536
Date: 18-Jul-2014	Solvent: dmso	SW: 32680 Hz	TE: 0 K	AQ: 1.57 sec, RD: 0.00 sec	Parameter file, XWIN-NMR Version 3.5

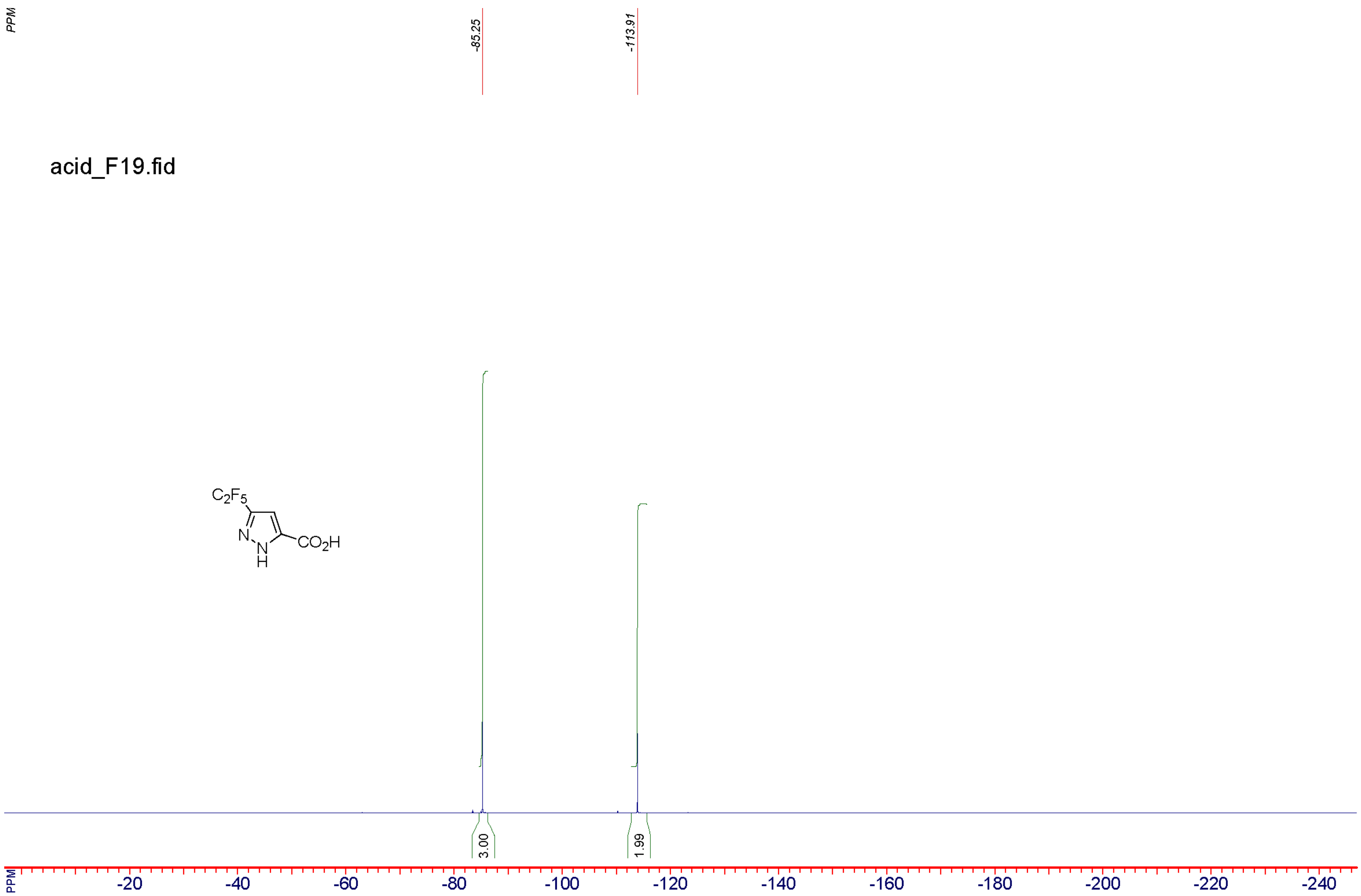

**File name: acid_F19.fid**	Operator:	SF: 376.3189 MHz	NSC: 0	PW: 3.00 usec, RG: 32	SI: 65536
Date: 20-May-2014	Solvent: cdcl3	SW: 94118 Hz	TE: 293 K	AQ: 0.34 sec, RD: 0.00 sec	⊓

Compound 26

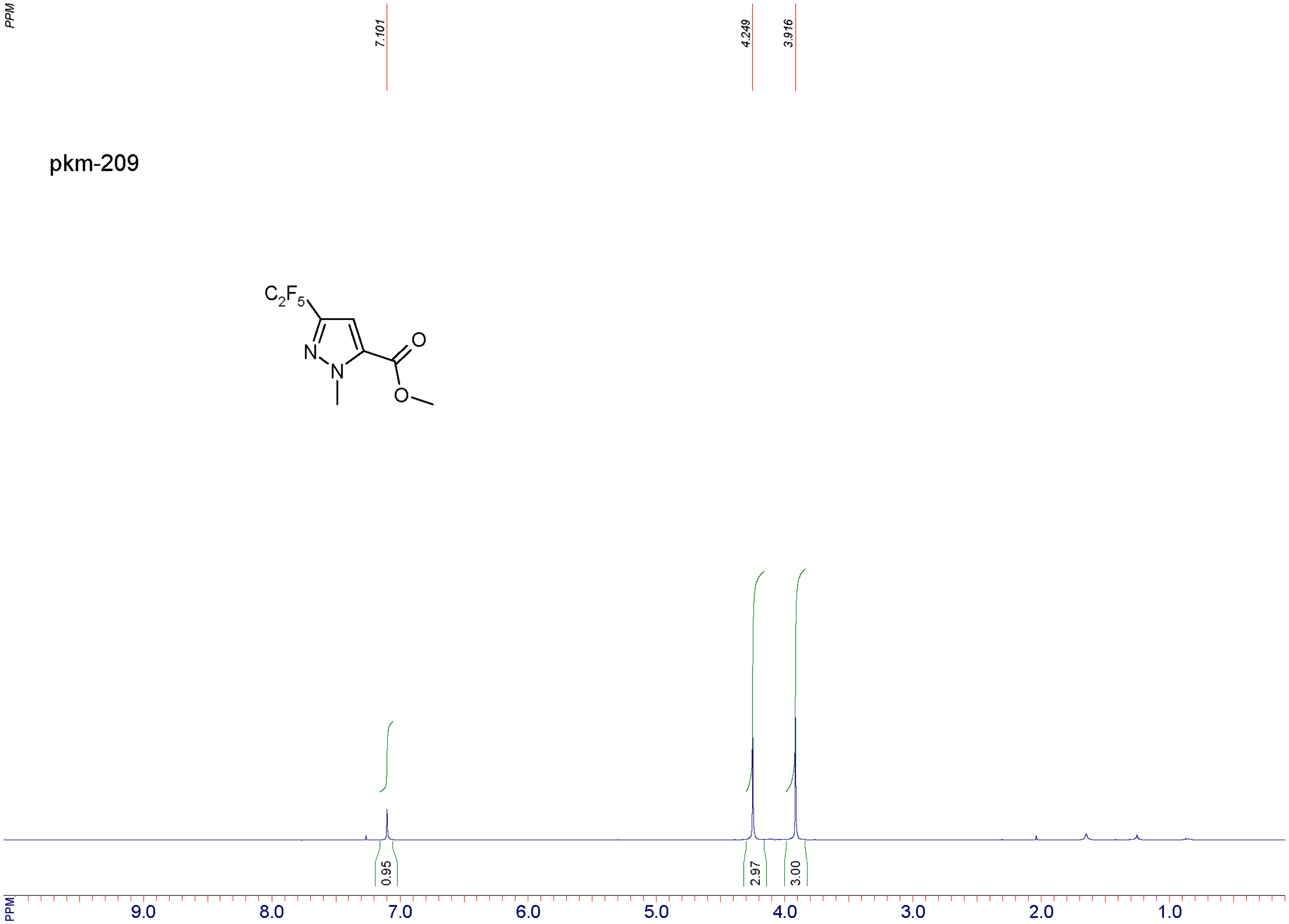

**File name: pkm-209**	Operator: root	SF: 499.9500 MHz	NSC: 1	PW: 0.00 usec, RG: 24	SI: 32768
Date: 16-May-2014	Solvent: CDCl3	SW: 8993 Hz	TE: 0 K	AQ: 1.82 sec, RD: 0.00 sec	Parameter file, XWIN-NMR Version 3.5

pkm209 C13

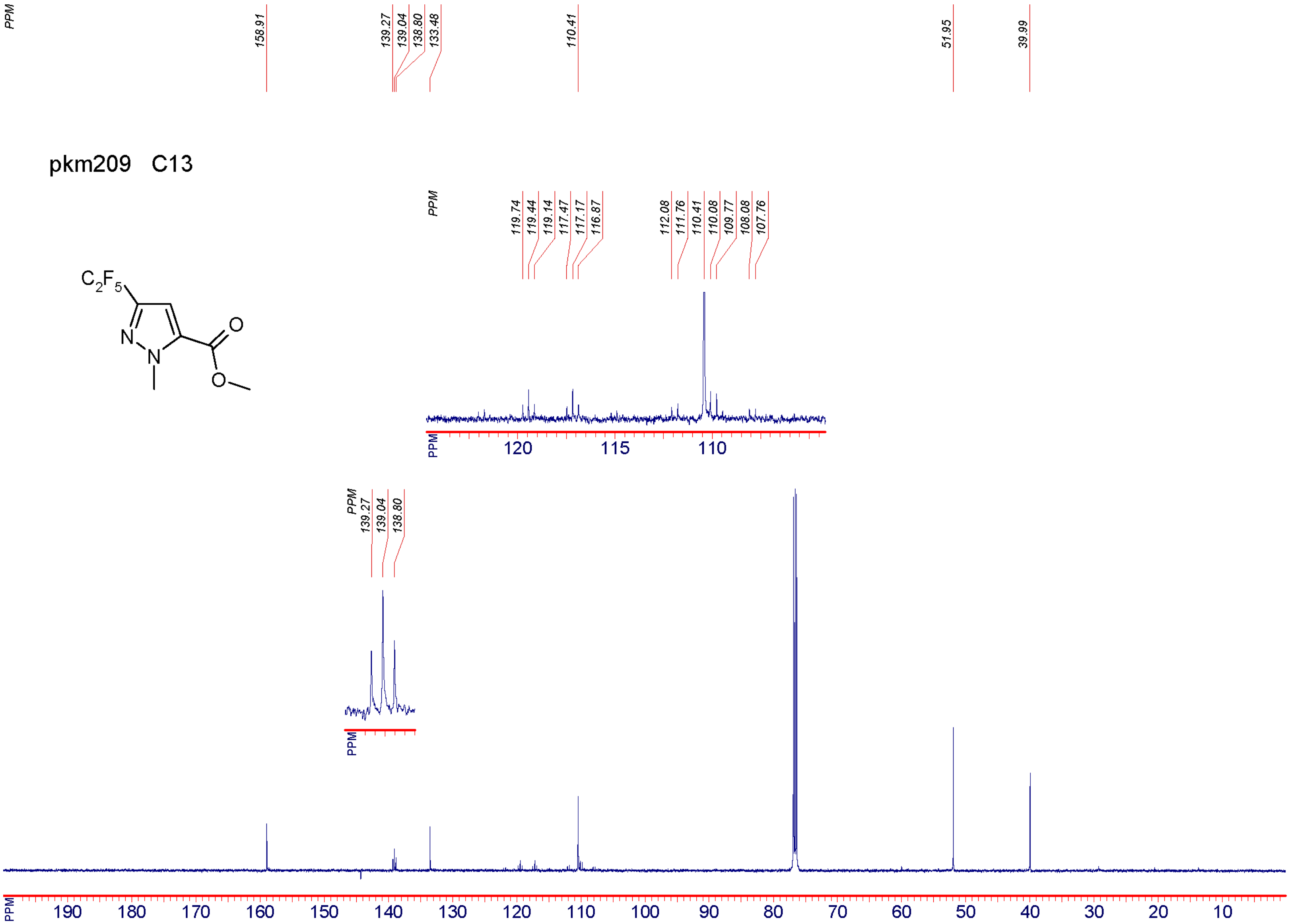

**File name: pkm209 C13**	Operator: root	SF: 125.7126 MHz	NSC: 1501	PW: 0.00 usec, RG: 51200	SI: 65536
Date: 18-May-2014	Solvent: CDCl3	SW: 32680 Hz	TE: 0 K	AQ: 1.57 sec, RD: 0.00 sec	Parameter file, XWIN-NMR Version 3.5

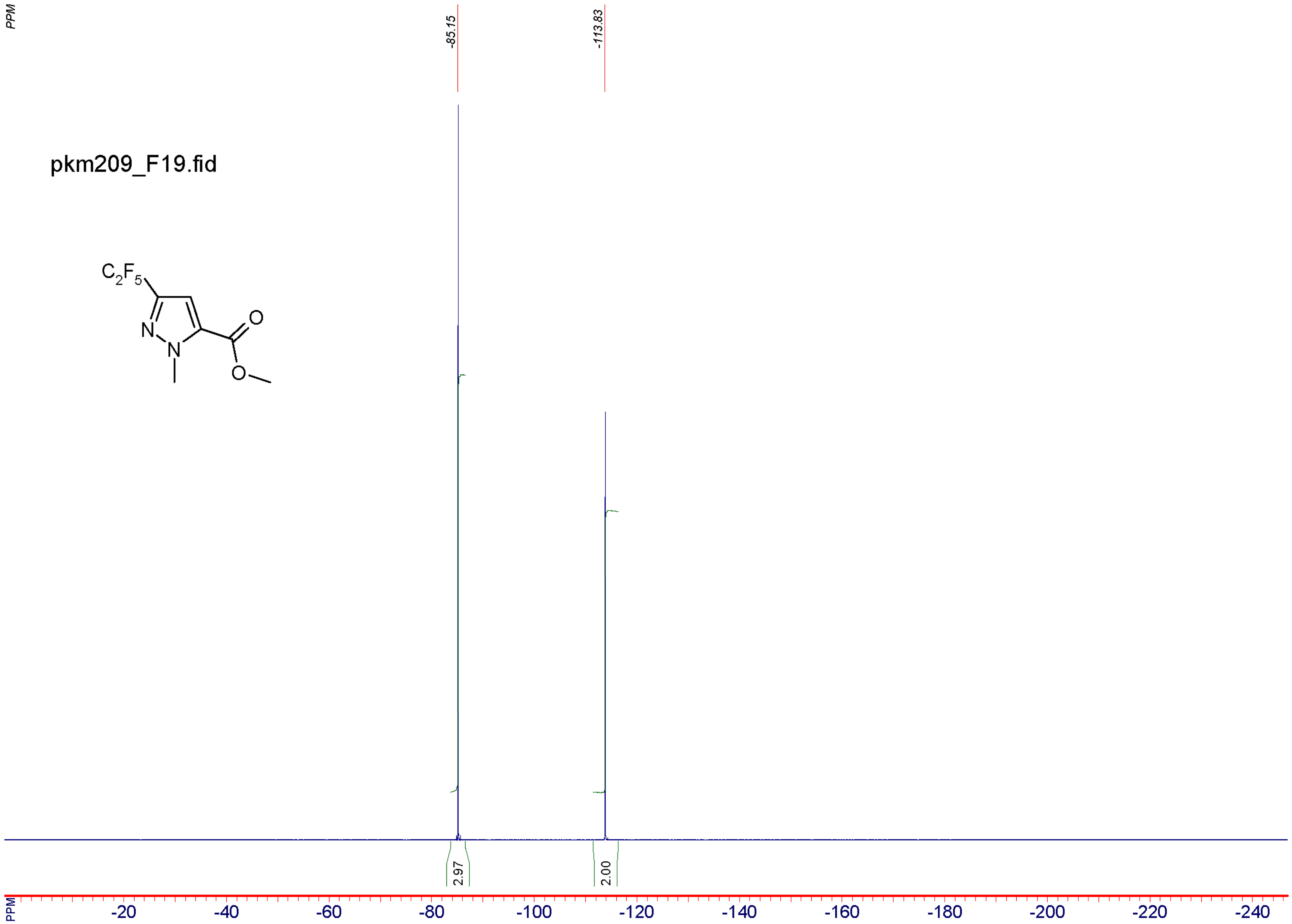

**File name: pkm209_F19.fid**	Operator:	SF: 376.3189 MHz	NSC: 0	PW: 3.00 usec, RG: 32	SI: 65536
Date: 20-May-2014	Solvent: cdcl3	SW: 94118 Hz	TE: 293 K	AQ: 0.34 sec, RD: 0.00 sec	⊓

Compound 27

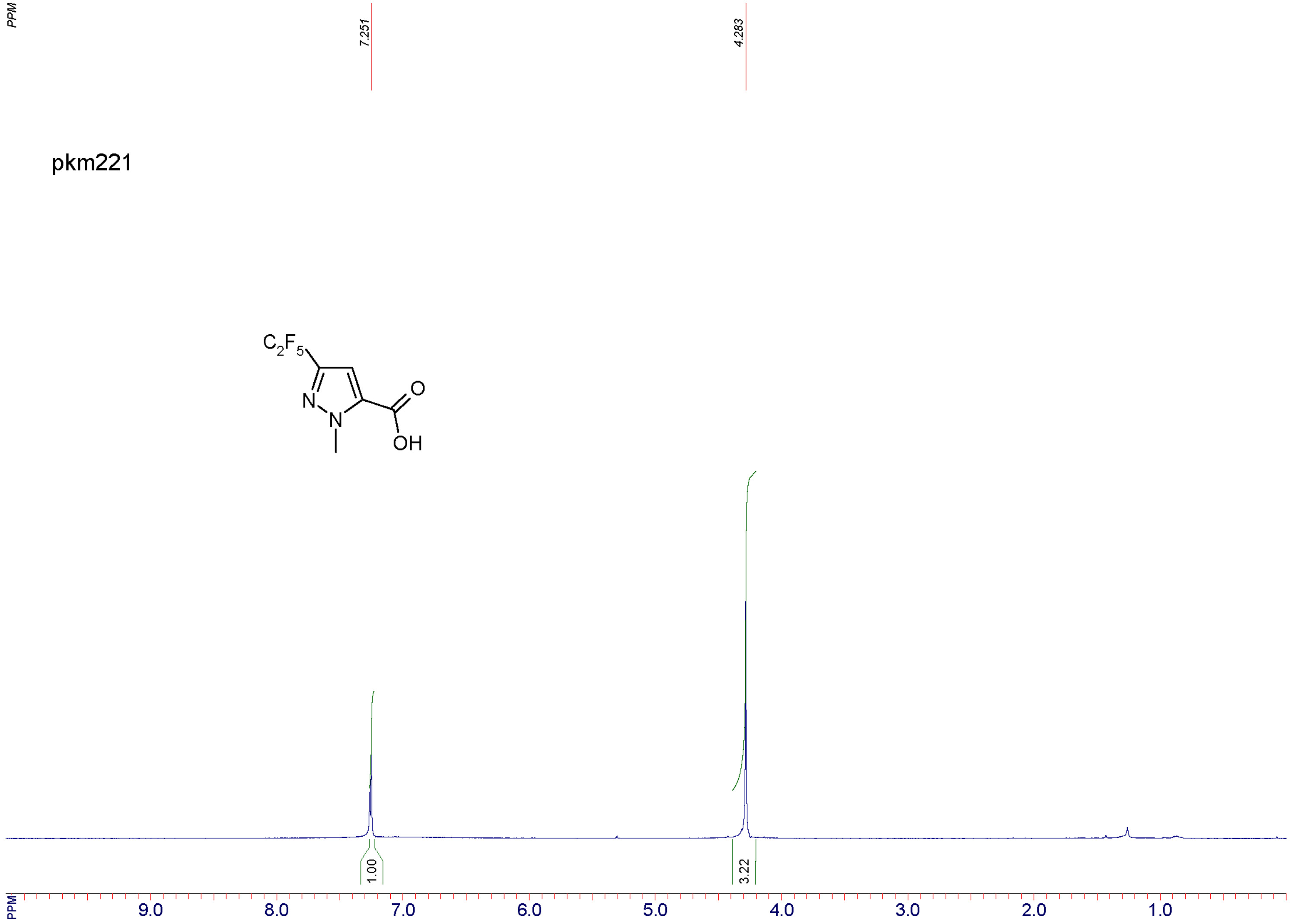

File name: pkm221	Operator: root	SF: 499.9500 MHz	NSC: 1	PW: 0.00 usec, RG: 24	SI: 32768
Date: 28-May-2014	Solvent: CDCl3	SW: 8993 Hz	TE: 0 K	AQ: 1.82 sec, RD: 0.00 sec	Parameter file, XWIN-NMR Version 3.5

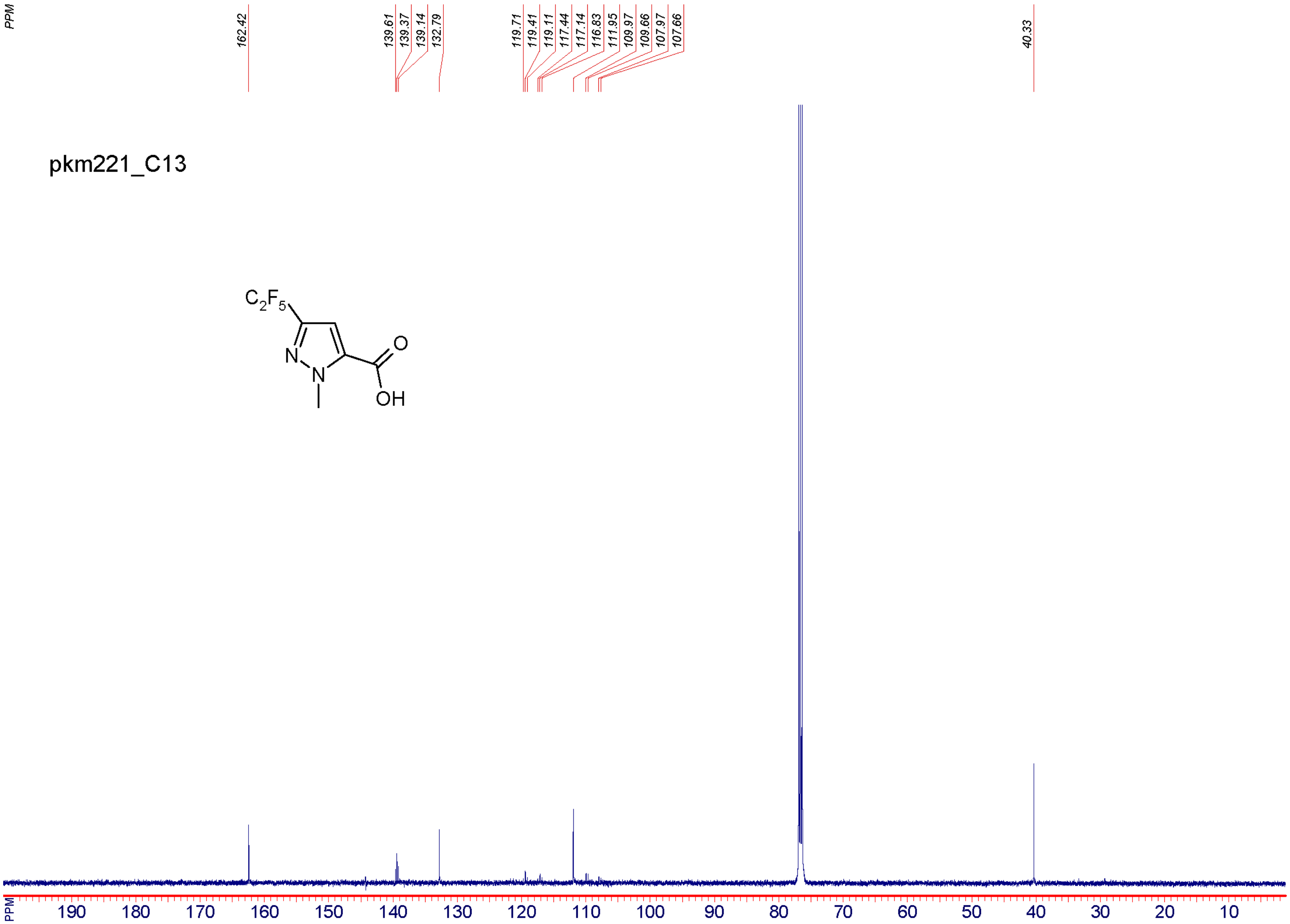

**File name: pkm221_C13**	Operator: root	SF: 125.7126 MHz	NSC: 3421	PW: 0.00 usec, RG: 51200	SI: 65536
Date: 31-May-2014	Solvent: dmso	SW: 32680 Hz	TE: 0 K	AQ: 1.57 sec, RD: 0.00 sec	Parameter file, XWIN-NMR Version 3.5

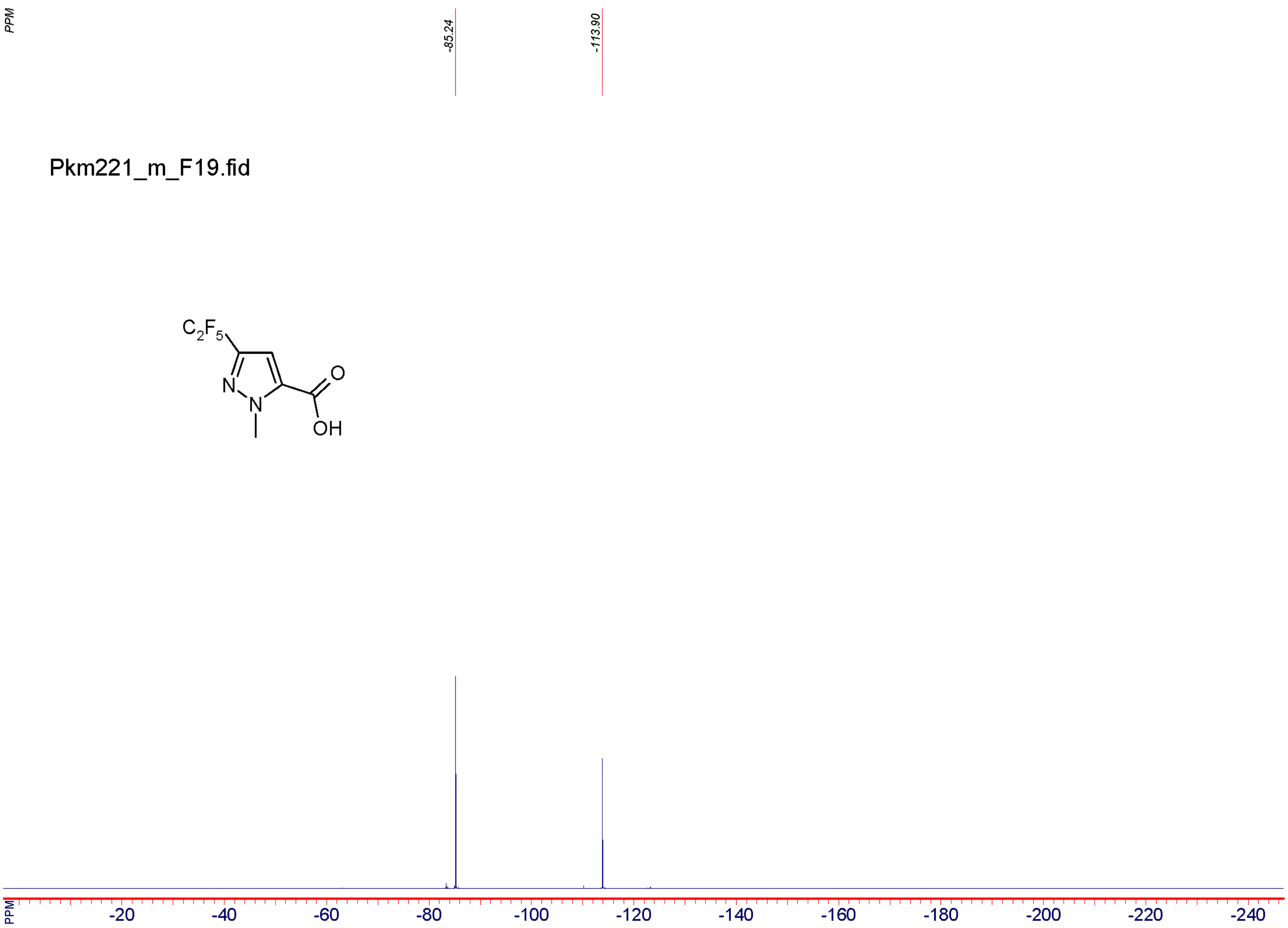

**File name: Pkm221_m_F19.fid**	Operator:	SF: 376.3189 MHz	NSC: 0	PW: 3.00 usec, RG: 32	SI: 65536
Date: 20-May-2014	Solvent: cdcl3	SW: 94118 Hz	TE: 293 K	AQ: 0.34 sec, RD: 0.00 sec	⊓
